# Small-molecule inhibitor cocktail promotes the proliferation of pre-existing liver progenitor cells

**DOI:** 10.1016/j.stemcr.2022.05.023

**Published:** 2022-06-30

**Authors:** Qingjie Fu, Shunsuke Ohnishi, Goki Suda, Naoya Sakamoto

**Affiliations:** 1 Department of Gastroenterology and Hepatology, Hokkaido University Graduate School of Medicine, Sapporo 060-8638, Japan; 2 Laboratory of Molecular and Cellular Medicine, Faculty of Pharmaceutical Sciences, Hokkaido University, Sapporo 060-0812, Japan

**Keywords:** liver progenitor cells, small molecules, cell differentiation, cell proliferation, liver development, regenerative medicine

## Abstract

A recent study showed that a cocktail of three small molecules, Y-27632, A83-01, and CHIR99021 (YAC), converts mature hepatocytes (MHs) into proliferative bipotent cells that can be induced into MHs and cholangiocytes in rats. However, when we reproduced these experiments, it was found that bipotent cells may be derived from resident liver progenitor cells (LPCs), whose proliferative activity was promoted by YAC. A simple and efficient sorting scheme was also developed in this study to harvest high-purity and high-yield LPCs. The inducible bipotency of purified LPCs was verified; in addition, they were found to spontaneously differentiate into hepatocytes and cholangiocytes due to changes in proliferative status even without induction. Moreover, during the differentiation process, some hepatocytes spontaneously reconverted to LPCs under certain conditions, such as the release of contact inhibition. These findings may improve our understanding of LPCs and provide a cell source for regenerative medicine.

## Highlights

• A small-molecule cocktail promotes rat liver progenitor proliferation *in vitro*
• Highly purified progenitors can be simply obtained by their physical properties
• Purified progenitors preferentially proliferate and then spontaneously differentiate
• Progenitor differentiation is closely related to varied proliferation signals

## Graphical abstract

**Figure:**
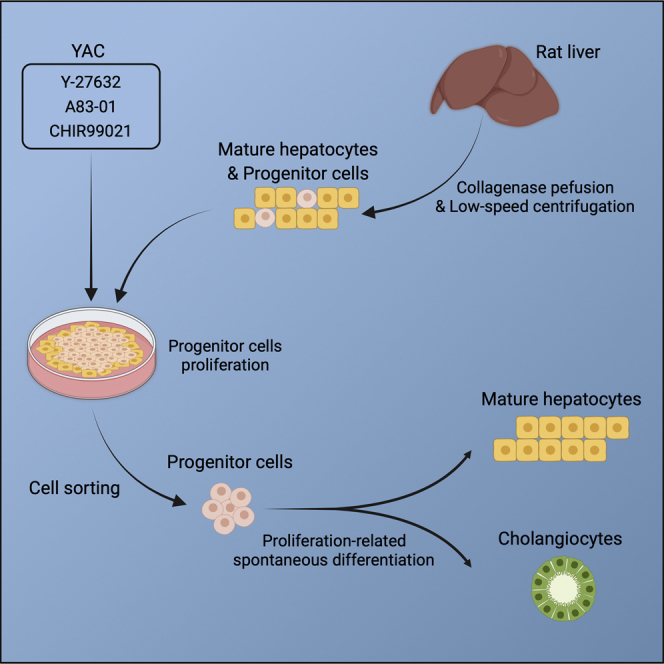

## Teaser

In this article, Ohnishi and colleagues showed that a small-molecule cocktail promotes the proliferation of rat liver progenitor cells. They also developed a simple and effective approach to obtain a large number of highly purified liver progenitor cells. At the same time, they revealed how progenitor cells spontaneously coordinate their proliferation and differentiation *in vitro*.

## Introduction

It is well known that the liver has a powerful regenerative capacity. Mature hepatocytes (MHs) and liver progenitor cells (LPCs), a type of proliferative cells that can differentiate into hepatocytes and cholangiocytes, are believed to be involved in liver regeneration (Espanol-Suner et al., 2012; Fausto and Campbell, 2003; Miyaoka et al., 2012). When the liver is injured and MH proliferation is inhibited, LPCs play a crucial role as building blocks for liver reconstruction (Fausto, 2004). The most cited theory is that LPCs, as a component of ductular reactions, originate in the canals of Hering (Theise et al., 1999); however, their origin is debatable and open to interpretation. MHs are also reported to convert into LPCs and reconstruct the liver (Tarlow et al., 2014). Notably, a combination of three small molecules, i.e., Y-27632 (Rho-associated kinase inhibitor), A83-01 (type 1 transforming growth factor β receptor inhibitor), and CHIR99021 (glycogen synthase kinase-3 inhibitor) (YAC), has been reported to revert rodent MHs to proliferative LPCs termed chemically induced liver progenitors (CLiPs) (Katsuda et al., 2017).

A sufficient understanding of LPCs will not only help comprehend how the liver functions, but will also be beneficial for therapeutic purposes. For patients with end-stage liver disease, liver transplantation is the only curative therapy (Dhawan et al., 2010); however, the shortage of donated organs limits this approach. Although MH transplantation has been recognized as an alternative treatment (Dhawan et al., 2010), great difficulties in expanding MHs *in vitro* restrict their clinical application (Bhatia et al., 2014; Guguen-Guillouzo and Guillouzo, 2010). Thus, transplanting LPCs seems to be a more reasonable option. The methods available currently for the purification of LPCs are mostly based on fluorescence-activated cell sorting (FACS) via cell labeling with specific antibodies (Liu et al., 2019b; Suzuki et al., 2008); however, for clinical application, the safety of antibody-conjugated cell transplantation is also of concern. Therefore, the generation of abundant and clinically available LPCs is a new challenge, and more feasible methods should be developed to attain this goal.

Here, we revisit how YAC works on LPCs and provide a simple and efficient strategy to obtain purified LPCs that may serve as a practical tool for studying liver regeneration and LPC transplantation. Also, we describe how LPC regulates differentiation in response to proliferation signals.

## Results

### Small-molecule inhibitor cocktail promotes the proliferation of resident LPCs

**Figure 1:**
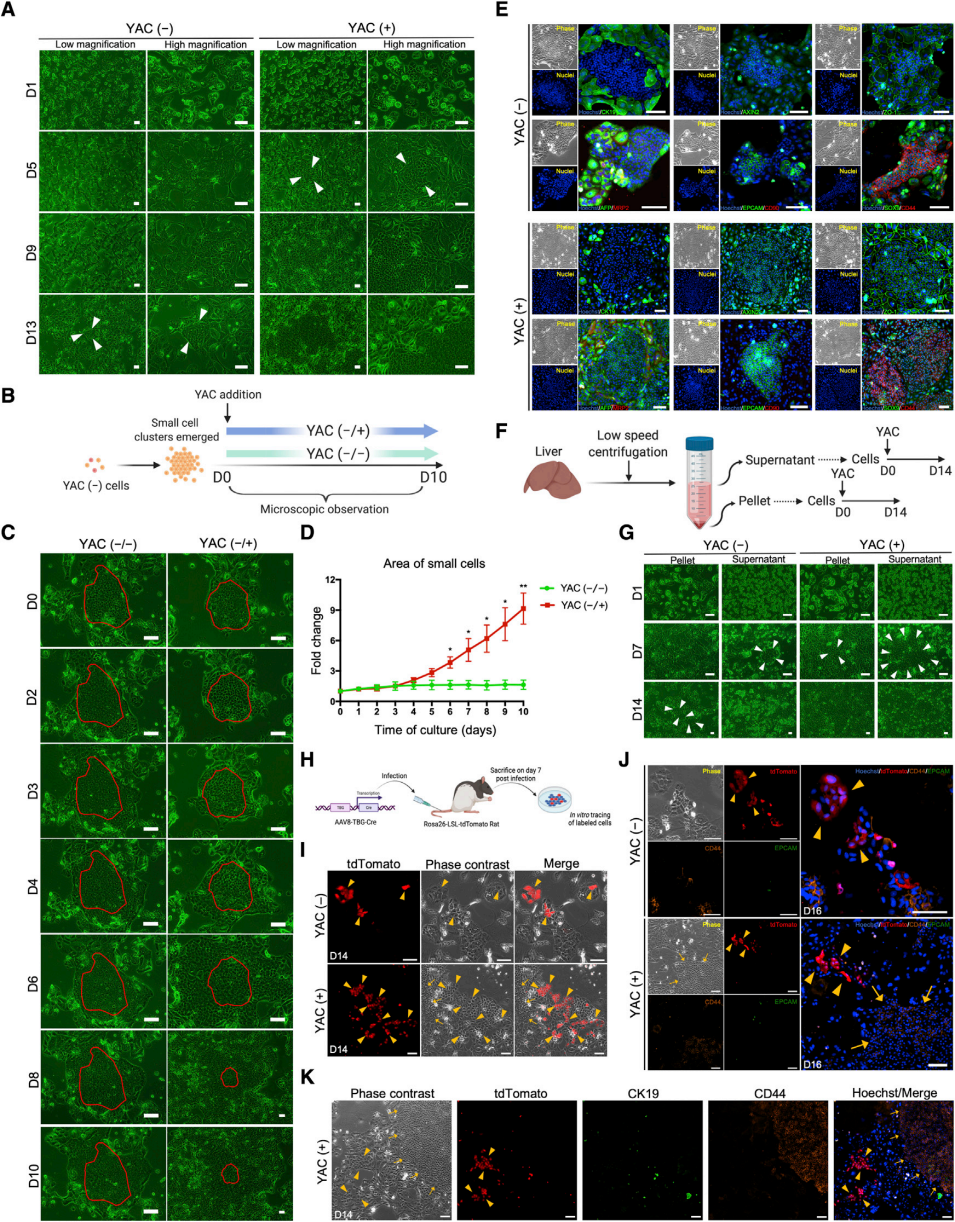
YAC enhances the proliferation of resident rat liver progenitor cells (LPCs; i.e., small cells) (A) Phase-contrast images of freshly isolated rat MHs cultured with or without YAC. MH, mature hepatocyte; YAC, the combination of Y-27632, A83-01, and CHIR99021; YAC (−), MH culture without YAC; YAC (+), MH culture with YAC. (B) Schematic representation of the supplementation of YAC (−) cells with YAC. According to the addition of YAC to YAC (−) cells, the culture conditions were termed YAC (−/−) and YAC (−/+). (C) Representative phase-contrast images of cells with or without additional YAC. The red closed loops denote the initial areas of small cells on day 0. (D) The area of small cells was measured at the indicated time points. The values are normalized to the initial area recorded on day 0. The data are expressed as the mean ± SD (n = 3 tracing areas), ^∗^p < 0.05; ^∗∗^p < 0.01. (E) Immunofluorescence staining of LPC markers CK19, AXIN2, AFP, EPCAM, CD90, SOX9, and CD44; the MH marker MRP2; and the tight junction marker ZO-1 in YAC (−) and YAC (+) cells. (F) Schematic representation showing the method used for isolating and culturing supernatant-derived cells and pellet-derived cells. (G) Phase-contrast images of supernatant-derived cells and pellet-derived cells cultured with or without YAC. (H) Schematic representation of the lineage tracing experiments of rat MHs. (I) Phase-contrast and fluorescence images of tdTomato^+^ MH-derived cells cultured with or without YAC on day 14 (D14). (J) Immunofluorescence staining of LPC markers CD44 and EPCAM in Rosa26-LSL-tdTomato rat cells cultured with or without YAC. (K) Immunofluorescence staining of cholangiocyte marker CK19 and LPC marker CD44 in Rosa26-LSL-tdTomato rat cells cultured with YAC. Scale bars, 100 μm. The arrowheads indicate the small cells that first appeared in corresponding culture conditions in (A) and (G). The arrowheads indicate the tdTomato^+^ cells and the arrows indicate typical small cells in (I), (J), and (K). See also Figure S1.

The combination of Y-27632, A83-01, and CHIR99021, referred to as YAC, has been suggested to convert MHs into culturable bipotent progenitor cells in a previous study (Katsuda et al., 2017). However, we found another possibility for the appearance of culturable cells when culturing rat MHs with YAC. As described in that study, a small hepatocyte culture medium (SHM) was used as the basal medium to culture the freshly isolated MHs (YAC (−) cells). Cells proliferated rapidly in the presence of YAC (YAC (+) cells), reaching a number of cells that was 2.51 ± 0.09 times greater than that of YAC (−) cells after 14 days of culture (Figures 1A and S1A). During cell culture, two types of cells that were morphologically distinct were observed. One of them was a small ovoid cell type that proliferated within a short period (Figure 1A). Although small-cell clusters were rare, they were observed in the subsequent culture of YAC (−) cells (Figure 1A). Small cells first emerged on day 7.00 ± 0.39 and day 11.38 ± 0.63 under YAC and YAC-free conditions, respectively (Figure S1B), which indicates that small cells proliferated faster under the effect of YAC, and a long-term culture demonstrated this difference significantly (Figure S1C). To verify whether YAC could promote small-cell proliferation selectively, we further cultured the non-YAC-induced small cells with/without YAC (Figure 1B). In the absence of YAC (YAC (−/−) cells), small cells sustained slow proliferation, whereas under YAC treatment (YAC (−/+) cells), small cells proliferated rapidly without accompanying large-cell growth (Figure 1C). During the 10-day culture, the area of small cells increased by 9.16 ± 0.77-fold under YAC stimulation, far exceeding the 1.64 ± 0.27-fold increase obtained after culture on SHM alone (Figure 1D). Interestingly, YAC did not play a role at the very beginning of the cell culture; rather, it began promoting small-cell proliferation between days 3 and 4 of culture (Figures 1C and 1D), in accordance with the results of a previous study (Katsuda et al., 2017).

Gene expression was analyzed by qRT-PCR in YAC-treated cells on day 14 and it revealed that the LPC marker expressions were upregulated compared with that of fresh MHs (Figure S1D), implying that LPCs were generated under YAC stimulation. To identify LPCs in the cell culture, we performed immunostaining using MH- and LPC-specific markers. The LPC markers EPCAM, CD44, and CD90 (occasionally) were expressed in small cells exclusively (Figures 1E and S1E). Conversely, the expression of another LPC marker CK19 (also a cholangiocyte marker) and the MH marker MRP2 was only observed in surrounding large cells (Figures 1E and S1E). Other widely used LPC markers, such as AFP, AXIN2, and SOX9, were expressed in both cell types (Figure 1E). Based on these results, although the expression pattern of the LPC-specific markers was not entirely consistent with the usual LPC profile, we considered that the small cells were LPCs and the surrounding large cells were mature cells. Furthermore, ZO-1 expression showed tight junctions between cells and revealed that small cells had a high nucleus-to-cytoplasm ratio (Figure 1E), which was in line with the description of LPCs provided in a previous article (Kohn-Gaone et al., 2016). In addition, the individual characteristics of YAC (+) cells did not change during the culture with YAC, compared with YAC (−) cells (Figure 1E), which indicated that YAC does not generate a brand-new cell type.

Since the absence of YAC did not affect the small-cell emergence, we hypothesized that small cells originated from pre-existing cells mingled in the MH fraction during liver cell isolation. In that process, MHs were predominant in the pellet after low-speed centrifugation, whereas other smaller cells were enriched in the supernatant (Chen et al., 2007). Thus, to verify our hypothesis, we cultured the cells obtained from the pellet and the supernatant, respectively (Figure 1F). Under YAC (−) conditions, visible small cells in cultured supernatant-derived cells appeared much earlier than those detected in cultured pellet-derived cells; moreover, in the presence of YAC, when small cells emerged in cultured pellet-derived cells, those derived from supernatant had already proliferated in large quantities (Figure 1G). These results met our expectations, namely, a greater number of pre-existing small cells was associated with the earlier appearance of small-cell clusters.

To explore the origin of small cells accurately, we performed genetic lineage tracing using AAV8-TBG-Cre and Rosa26-LSL-tdTomato rat (Figure 1H) (Igarashi et al., 2016). Although the labeling efficiency was low (Figures S1F and S1H), labeled cells were observed to divide on day 3 (Figure S1G) and were confirmed to be able to proliferate regardless of YAC stimulation (Figure 1I). However, the proliferative tdTomato^+^ cells were obviously different from the typical small cells in morphology (Figures 1I–1K) and they were hardly expressed CD 44 and EPCAM (Figure 1J), which were confirmed expressing in Rosa26-LSL-tdTomato rat-derived small cells (Figure S1K). Moreover, we sorted only tdTomato^+^ cells to analyze (Figure S1H), but these cells did not proliferate nor express LPC markers (Figures S1I and S1J) even culturing with YAC. A previous study also indicated that some cholangiocytes may be mislabeled due to the expression of TBG (Lee et al., 2020), but no CK19 expression was observed in the labeled cells as well (Figure 1K).

Altogether, these results showed that YAC promoted the proliferation of pre-existing LPCs. Considering the apparent difference in morphology between small cells and surrounding cells, we wondered if they could be separated based on this parameter for further analysis.

### A large number of highly purified LPCs can be obtained using FACS

**Figure 2:**
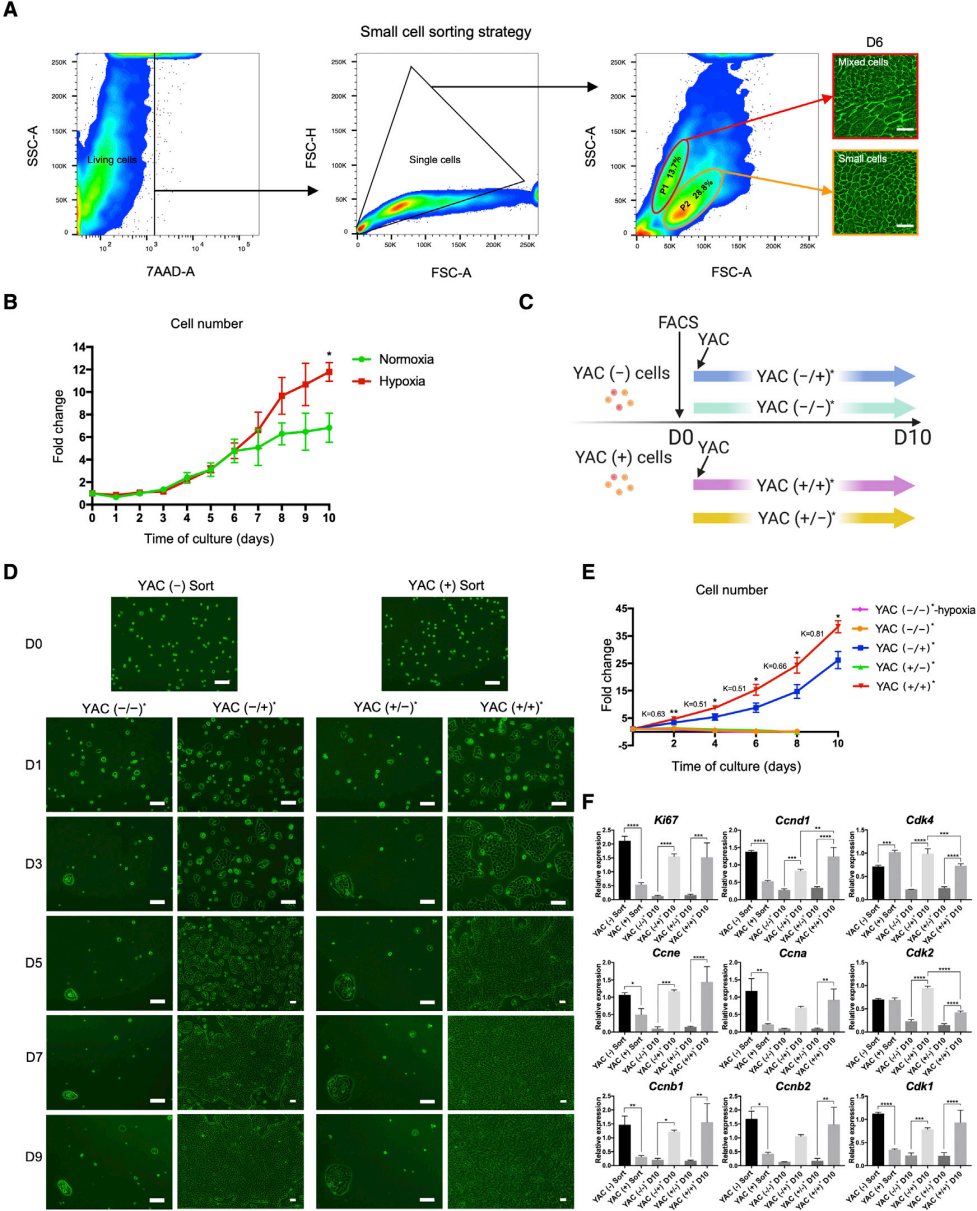
Highly purified small cells can be efficiently sorted by fluorescence-activated cell sorting (FACS) (A) Strategy used for isolating small cells from YAC (+) cells. Phase-contrast images show the morphology of sorted cells from the P1 and P2 fraction cultured with YAC for 6 days. FSC, forward scatter; SSC, side scatter; 7-AAD, 7-aminoactinomycin D. (B) The number of YAC (+) Sort cells cultured with YAC under normoxia (20% O_2_) and hypoxia (5% O_2_). Values are normalized to the initial number of cells recorded on day 0. (C) Schematic representation of the protocol used for supplementing sorted small cells with YAC. According to whether YAC was applied before and after FACS, the culture conditions were termed YAC (−/−)^∗^, YAC (−/+)^∗^, YAC (+/−)^∗^, and YAC (+/+)^∗^. (D) Phase-contrast images of sorted small cells cultured in the YAC (−/−)^∗^, YAC (−/+)^∗^, YAC (+/−)^∗^, and YAC (+/+)^∗^ conditions. (E) The number of sorted small cells under the corresponding conditions was counted at the indicated time points. Values are normalized to the initial number of cells recorded on D0. The index K is defined as the ratio of the YAC (−/+)^∗^ cell proliferation rate to that of YAC (+/+)^∗^ cells. The slope between the two time points is regarded as the ratio of cell proliferation. YAC (−/−)^∗^-Hypoxia, YAC (−/−)^∗^ cells cultured in the hypoxic condition (5% O_2_). (F) Cell-cycle-related gene expression in freshly sorted small cells and in those cells cultured under YAC (−/−)^∗^, YAC (−/+)^∗^, YAC (+/−)^∗^, and YAC (+/+)^∗^ conditions. The data are shown as the mean ± SD (n = 3 independent experiments), ^∗^p < 0.05; ^∗∗^p < 0.01; ^∗∗∗^p < 0.001; ^∗∗∗∗^p < 0.0001. Scale bars in (A), 50 μm; in (D), 100 μm. See also Figure S2.

We attempted to isolate small cells by FACS from YAC (+) cells that were cultured for 14 days and fully displayed the morphological characteristics. Although it was inconsistent with our expectation that cell clusters with distinct forward scatter would appear due to their different sizes, cells were still clearly divided into two groups (Figure 2A). We sorted these two cell fractions and further cultured with YAC. Cells sorted from P1 still contained two cell types, while cells sorted from P2 (YAC (+) Sort cells) exhibited the small-cell morphology exclusively and generally expressed LPC markers after culturing for 6 days (Figures 2A and S2A). Moreover, the expression of a set of LPC markers in cultured P2 cells was higher than that in cultured P1 cells at both mRNA level and protein level (Figures S2B and S2C). These results demonstrated that we could easily obtain highly purified small cells, i.e., LPCs, by FACS. The diameter of the purified small cells was 16.8 μm on average, which was much smaller than 24.5 μm in freshly isolated MHs (Figure S2D). To investigate whether YAC has effects other than promoting cell proliferation, we sorted small cells from YAC (−) cells (YAC (−) Sort cells) using the same strategy for comparison. Because the proportion of small cells obtained in the absence of YAC was too low, we extended the culture period and sorted cells on day 22 (Figure S2E). Regardless of YAC stimulation, the gene expression of several LPC markers, such as *Afp* and *Sox9*, was almost the same in either sorted cell (on D22); however, that of other genes, such as *Epcam*, *Cd44*, and *Foxj1*, was significantly different in the presence of YAC (Figure S1D). Besides, sorted cells’ *Alb* expression was constant at an extremely lower level compared with fresh hepatocytes (Figure S1D). Based on these results, we re-confirmed that YAC does not markedly alter the features of small cells. A prior study demonstrated that the growth of biphenotypic human hepatocytes could be further extended in a hypoxic condition of 5% O_2_ (Zhang et al., 2018); thus, we also cultured the sorted small cells using YAC in combination with hypoxia. Notably, hypoxia further enhanced small-cell proliferation from day 7 (Figure 2B), which might provide a more efficient procedure for obtaining abundant cells.

To further elucidate the principle of YAC-induced small-cell proliferation and investigate whether YAC is required to maintain their proliferation, we cultured the sorted small cells in various ways, considering the timing of YAC application (Figure 2C). When YAC (−) Sort cells continued to be cultured without YAC (YAC (−/−)^∗^ cells), they could hardly proliferate; furthermore, YAC (+) Sort cells also exhibited a deceleration in the proliferation rate after the withdrawal of YAC (YAC (+/−)^∗^ cells) (Figures 2D and 2E), suggesting that YAC is essential for maintaining the continuous proliferation of small cells. Regardless of the presence or absence of YAC in culture before sorting, sorted small cells showed a strong proliferative capacity under YAC treatment; among them, cells that were continuously stimulated by YAC (YAC (+/+)^∗^ cells) maintained a rapid growth throughout the experiment, and cells cultured with YAC later (YAC (−/+)^∗^ cells) showed a distinct proliferative trend from day 3 (Figures 2D and 2E). The index K was defined as the ratio of the YAC (−/+)^∗^ cell proliferation rate to that of the YAC (+/+)^∗^ cells. As the cultures continued, the K value gradually increased and reached a value close to 1 (Figure 2E), which indicates that the proliferation rate induced by YAC eventually tended to be consistent, despite the application of YAC stimulation at different time points. Moreover, culturing small cells under hypoxia alone (YAC (−/−)^∗^ Hypoxia cells) did not benefit proliferation to a greater extent compared with YAC (−/−)^∗^ cells (Figure 2E), implying that although the combination of hypoxia and YAC could further promote small-cell proliferation, hypoxia cannot replace the crucial pro-proliferative role of YAC. Proliferation-related gene expression was assessed by qRT-PCR, which showed that *Ki67* and most cell-cycle-associated gene expression in YAC (−) Sort cells was higher than in YAC (+) Sort cells (Figure 2F), indicating that small cells have an innate potential for self-renewal. In YAC (+) Sort cells, the downregulation of those genes was attributed to the contact inhibition caused by the YAC-induced rapid small-cell proliferation, which was confirmed by analyzing sorted cells at different levels of confluence (Figure S2F). Conversely, the *Cdk4* upregulation and stable *Cdk2* expression (Figure 2F), relating to the G1 and G1/S phases of the cell cycle, respectively (Figure S2G), suggests that YAC (+) Sort cells were still undergoing active substance synthesis and were ready for entering the cell-division stage. Corresponding to the cell proliferation profiles mentioned above, YAC significantly promoted the proliferation-related gene expression in sorted small cells, including *Cdk4* and *Cdk2*; conversely, the expression of those genes was kept lower because of the absence or withdrawal of YAC (Figure 2F). Furthermore, by adding YAC in YAC (−) Sort cells, we confirmed that YAC accelerated small-cell proliferation by shortening the G1 phase (Figure S2H).

We also sorted small cells from YAC-treated pellet- and supernatant-derived cells, with no noticeable difference in small-cell proportion (Figure S2I). The sorted small cells exhibited the same morphology and growth patterns (Figure S2J) with equivalent LPC marker gene expression (Figure S2K), proving that the small cells present in the pellet and supernatant were identical. It is worth mentioning that nonparenchymal cells (NPCs) occasionally appeared among cultured supernatant-derived sorted cells (Figure S2L), probably because they were also small-sized cells and could not be thoroughly eliminated even by FACS.

Using the method described above, we could easily and efficiently obtain a substantial amount of highly purified LPCs. To verify if these LPCs retained their function under YAC stimulation, next we induced the purified small cells to differentiate into MHs and cholangiocytes.

### YAC-treated LPCs retain the bipotentiality to differentiate into MHs and cholangiocytes

**Figure 3:**
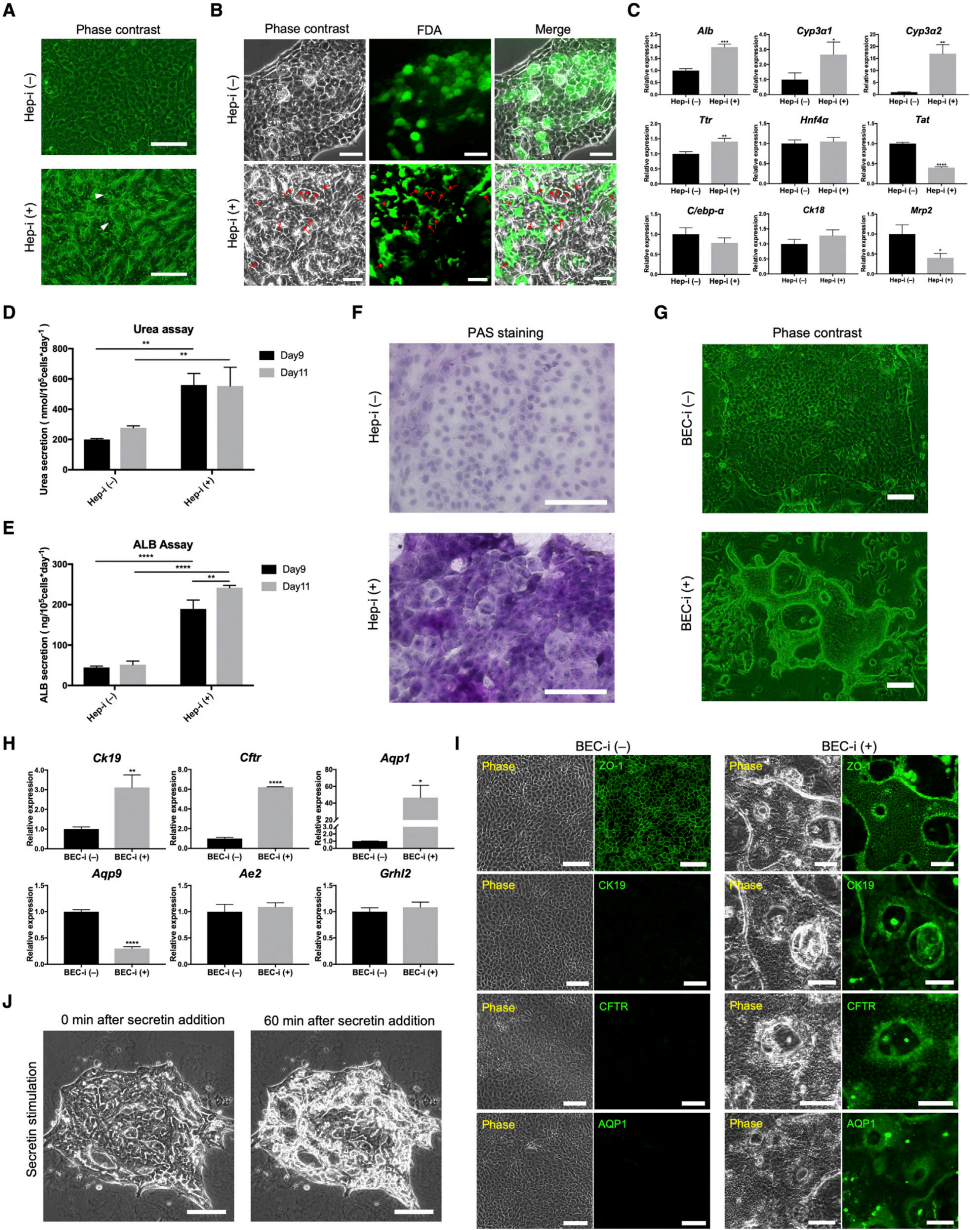
Small cells exhibit bipotentiality for being induced into both MHs and cholangiocytes (A) Phase-contrast images of the sorted small cells with or without hepatic induction. The arrowheads indicate the binucleate cells. Hep-i (−), sorted small cells cultured with YAC alone; Hep-i (+), sorted small cells cultured under hepatic induction. (B) Uptake and secretion of fluorescein diacetate (FDA) by sorted small cells with or without hepatic induction. The arrows indicate the accumulation of hydrolyzed FDA in the canaliculi-like structures. (C) MH-related genes expression in Hep-i (−) cells and Hep-i (+) cells. (D) Urea synthesis after completing conventional (D9) and extended (D11) hepatic induction. Values are normalized to the mean number of cells recorded on D9 or D11, and the corresponding culture period. (E) Albumin (ALB) secretion after completing normal (D9) and extended (D11) hepatic induction. Values are normalized to the mean number of cells recorded on D9 or D11, and the corresponding culture period. (F) The glycogen synthesis and storage capacity of Hep-i (+) cells were investigated by periodic-acid Schiff (PAS) staining. (G) Phase-contrast images of the sorted small cells with or without cholangiocytic induction. BEC-i (−), sorted small cells cultured without cholangiocytic induction; BEC-i (+), sorted small cells cultured under cholangiocytic induction. (H) Cholangiocyte-related gene expression in BEC-i (−) and BEC-i (+) cells. (I) Immunofluorescence staining of cholangiocyte markers CK19, CFTR, and AQP1, and the tight junction marker ZO-1 in BEC-i (−) and BEC-i (+) cells. (J) Phase-contrast images of BEC-i (+) cells before and 60 min after secretin stimulation. See also Video S1. The data are shown as the mean ± SD (n = 3 independent experiments), ^∗^p < 0.05; ^∗∗^p < 0.01; ^∗∗∗^p < 0.001; ^∗∗∗∗^p < 0.0001. Scale bars in (B), 50 μm; in (A), (F), (G), (I), and (J), 100 μm. See also Figure S3.

We used a modified protocol based on a previous study (Kamiya et al., 2002) to induce the hepatocytic differentiation of the YAC (+) Sort cells (Figure S3A). Small cells exposed to hepatic stimulation (Hep-i (+) cells) exhibited a typical MH morphology, such as a polygonal appearance, dual nucleus, and reduced nucleus-to-cytoplasm ratio (Figure 3A). Furthermore, we performed a fluorescein diacetate (FDA) hydrolysis assay and observed fluorescence in the canaliculi-like structures in Hep-i (+) cells, demonstrating induced cell secretory function (Figure 3B). In contrast, the fluorescence in uninduced cells (Hep-i (−) cells) remained inside the cells (Figure 3B). Hep-i (+) cells exhibited a higher expression of several genes related to hepatocytic function, such as *Alb*, *Cyp3a1*, *Cyp3a2*, and *Ttr*, compared with Hep-i (−) cells, whereas other genes, such as *Hnf4α*, *C/ebp-α*, *Ck18*, *Mrp2,* and *Tat*, did not vary significantly or were downregulated (Figure 3C). The high expression of ALB, CK18, HNF4A, and MRP2 showed that Hep-i (+) cells gained MH characteristics; however, Hep-i (−) cells showed almost the same levels as well (Figure S3B). That may be related to the spontaneous hepatic differentiation of Hep-i (−) cells (more hereof later). Moreover, Hep-i (+) cells were more active in urea synthesis and ALB secretion than Hep-i (−) cells, and prolonging the induction period further enhanced these capabilities (Figures 3D and 3E). In addition, this additional improvement in MH physiological function was verified by the upregulation of *Alb* and *Cyp3a2* (Figure S3C), and these gradations showed a continuous process of differentiation into MHs. Glycogen assay, as evaluated by periodic-acid Schiff (PAS) staining, also showed that Hep-i (+) cells synthesized and stored more glycogen (Figure 3F). Along with induction into hepatocytes, small cells lost the characteristics of LPCs, as confirmed by the LPC marker gene expression (Figure S3D).

Next, we induced YAC (+) Sort cells into cholangiocytes using a previously reported method (Katsuda et al., 2017; Figure S3E). Small cells that were simply co-cultured with mouse embryonic fibroblasts (BEC-i (−) cells) retained the oval morphology and proliferated as a cell monolayer, while the proliferation of cholangiocytic-induced cells (BEC-i (+) cells) was slowed down with forming a tubular structure (Figure 3G). BEC-i (+) cells expressed a higher level of cholangiocyte marker *Ck19* than BEC-i (−) cells, and *Cftr* and *Aqp1* expression was also increased in BEC-i (+) cells; however, several other cholangiocyte-associated genes, such as *Aqp9*, *Ae2*, and *Crhl2*, were downregulated or remained at the same level (Figure 3H). Consistent with these results, the expression of CK19, CFTR, AQP1, and ZO-1 was confirmed by immunostaining, with particularly high expression observed around the tubular structure (Figure 3I). Notably, ZO-1 expression clearly showed how the tubular structure was formed, similar to that of the intrahepatic bile duct (Rao and Samak, 2013), the lumen was lined with a monolayer of cholangiocyte-like cells with tight junctions sealing the paracellular spaces (Figure S3F). Secretin was used to evaluate the ability to transport water (Nishikawa et al., 2013); the luminal space of BEC-i (+) cells was enlarged under secretin stimulation (Figure 3J and Video S1), showing their secretory properties.

Video S1. The changes in luminal space between BEC-i (+) cells within 1 h of secretin stimulation, related to Figure 3

Based on these results, we demonstrated that YAC-treated small cells could still differentiate into MHs and cholangiocytes while gaining significant proliferation capacity.

### YAC-treated small cells can differentiate into hepatocytes and cholangiocytes spontaneously

**Figure 4:**
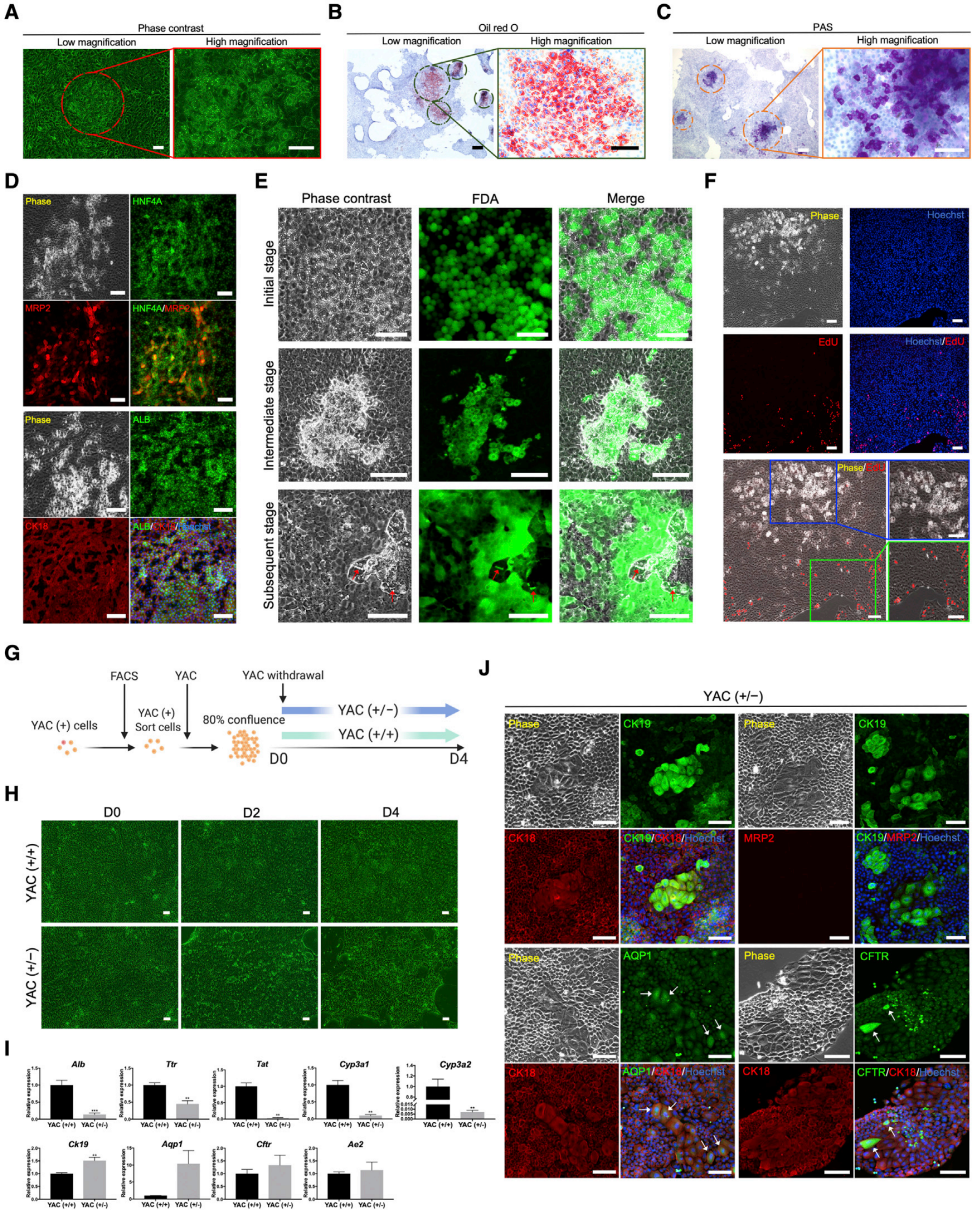
Small cells possess a capacity for spontaneous maturation associated with the cell proliferation status (A) Emergence of droplet-like components in cultured sorted small cells under YAC stimulation. (B) The identification of droplet-like components was performed using oil red O staining, indicating their identity as lipid droplets. The dark-green closed loops indicate the positions of cells containing lipid droplets (LDCs) in cell clusters. (C) The glycogen synthesis and storage capacity of LDCs were investigated by PAS staining. The orange closed loops denote the position of LDCs in cell clusters. (D) The expression of MH markers HNF4A, MRP2, ALB, and CK18 was confirmed in LDCs by immunofluorescence staining. (E) Uptake and secretion of FDA by spontaneously matured small cells at different stages. The arrows indicate the accumulation of hydrolyzed FDA in canaliculi-like structures in totally maturated small cells (subsequent stage). (F) The proliferation status of LDCs was verified by EdU assay. A magnified image of LDCs is shown in the blue box, and a magnified image of peripheral cells is shown in the green box. (G) Schematic representation of the method used for culturing proliferated small cells with YAC withdrawal. YAC (+/−), YAC withdrawal from massively proliferated sorted small cells; YAC (+/+), YAC retention in sorted small cells cultured with YAC. (H) Phase-contrast images of proliferated small cells cultured with or without YAC withdrawal. (I) MH- and cholangiocyte-associated gene expression in small cells cultured with or without YAC withdrawal. The data are expressed as the mean ± SD (n = 3 independent experiments), ^∗∗^p < 0.01, ^∗∗∗^p < 0.001. (J) Immunofluorescence staining of cholangiocyte markers CK18, CK19, AQP1, and CFTR, and the MH marker MRP2 in small cells cultured with YAC withdrawal. The arrows indicate cells expressing AQP1 or CFTR. Scale bars in (A) high magnification, 50 μm; in (A) low magnification, (B) high magnification, (C) high magnification, (D), (E), (F), (H), and (J), 100 μm; in (B) low magnification, (C) low magnification, 300 μm. See also Figure S4.

During a long-term culture of sorted small cells with YAC, we incidentally observed that droplet-like components appeared in the cytoplasm of several cells (Figures 4A and S4A), which were recognized as lipid droplets by oil red O staining (Figures 4B and S4B). It is well known that lipid storage is a feature of MHs; therefore, we inferred that those cells containing lipid droplets (LDCs) were MHs. PAS staining confirmed their capacity for glycogen synthesis (Figure 4C), and immunostaining indicated that they expressed higher levels of HNF4A, MRP2, ALB, and CK18 than normal small cells (Figure 4D), demonstrating that LDCs were indeed MHs. These results suggest that, even without induction, small cells can spontaneously differentiate into hepatocytes. Subsequently, we verified that this process was generally divided into three stages: in the initial stage, cells maintained a small-cell morphology with emergence of cytosolic lipid droplets; in the intermediate stage, cells presented the polygonal features of MHs without significant variation in cell size, which was accompanied by a slight decrease in lipid droplets; in the subsequent stage, cells became larger, exhibited visible MH morphological features, and showed secretory functions (Figures 4E and S4A). Moreover, an FDA assay demonstrated that cells involved in a maturation process possessed higher esterase activity (Figure 4E), revealing the enhanced protein-synthesis capacity of MHs. After culturing small cells further, we found that additional LDCs emerged (Figure S4C), suggesting that automatic entry into hepatocytic maturation is a common event in appropriate conditions. Interestingly, LDCs always first emerged in the center of cell clusters (Figures 4A−4C and S4B), where cells may not proliferate because of contact inhibition. Thus, we supposed that this spontaneous conversion was related to their proliferation situation. As expected, maturating cells stopped proliferating, or it should be explained that cells that had stopped proliferating initiated a process of spontaneous hepatocytic differentiation; in contrast, peripheral cells in clusters retained a robust proliferative capacity, as confirmed by EdU assay (Figure 4F).

We demonstrated that YAC played an essential role in maintaining small-cell proliferation, as well as in their differentiation after cells stopped proliferating. Subsequently, we wondered if small cells would enter the spontaneous hepatocytic maturation process if YAC was withdrawn when cells reached confluence. We discontinued YAC when small cells approached 80% confluence (Figure 4G). Unexpectedly, masses of cells died after YAC withdrawal (YAC (+/−) cells), and MH-shaped cells were barely observed (Figure 4H), suggesting that the persistence of proliferation-stimulation signals was also necessary for cell survival, and the loss of contact inhibition or/and the lack of YAC suspended the maturation of small cells. Furthermore, several cells with larger size and lower nucleus-to-cytoplasm ratio emerged (Figure S4D), indicating that some phenotypic conversion occurred in small cells. qRT-PCR demonstrated that small cells began to differentiate into cholangiocytes rather than hepatocytes after YAC withdrawal (Figure 4I), and enhanced CK19 expression was confirmed in emerging large cells by immunostaining (Figure 4J). Moreover, expression of CK18 but not MRP2 supported their transition into cholangiocytes, and the detection of AQP1 and CFTR proved their secretory function (Figure 4J). Besides, some small cells with decreased nucleus-to-cytoplasm ratio were also observed (Figure S4E). Similar to those large cells, they also expressed cholangiocyte markers rather than MH markers (Figure S4F), which likely demonstrated the continuity of conversion from small cells to cholangiocytes.

Without additional induction, small cells exhibited spontaneous bilineage-differentiation capacity associated with the status of cell proliferation, which provides a useful experimental model to study differentiation mechanisms under conditions resembling physiological status.

### Differentiated hepatocytes can re-dedifferentiate into LPCs spontaneously

**Figure 5:**
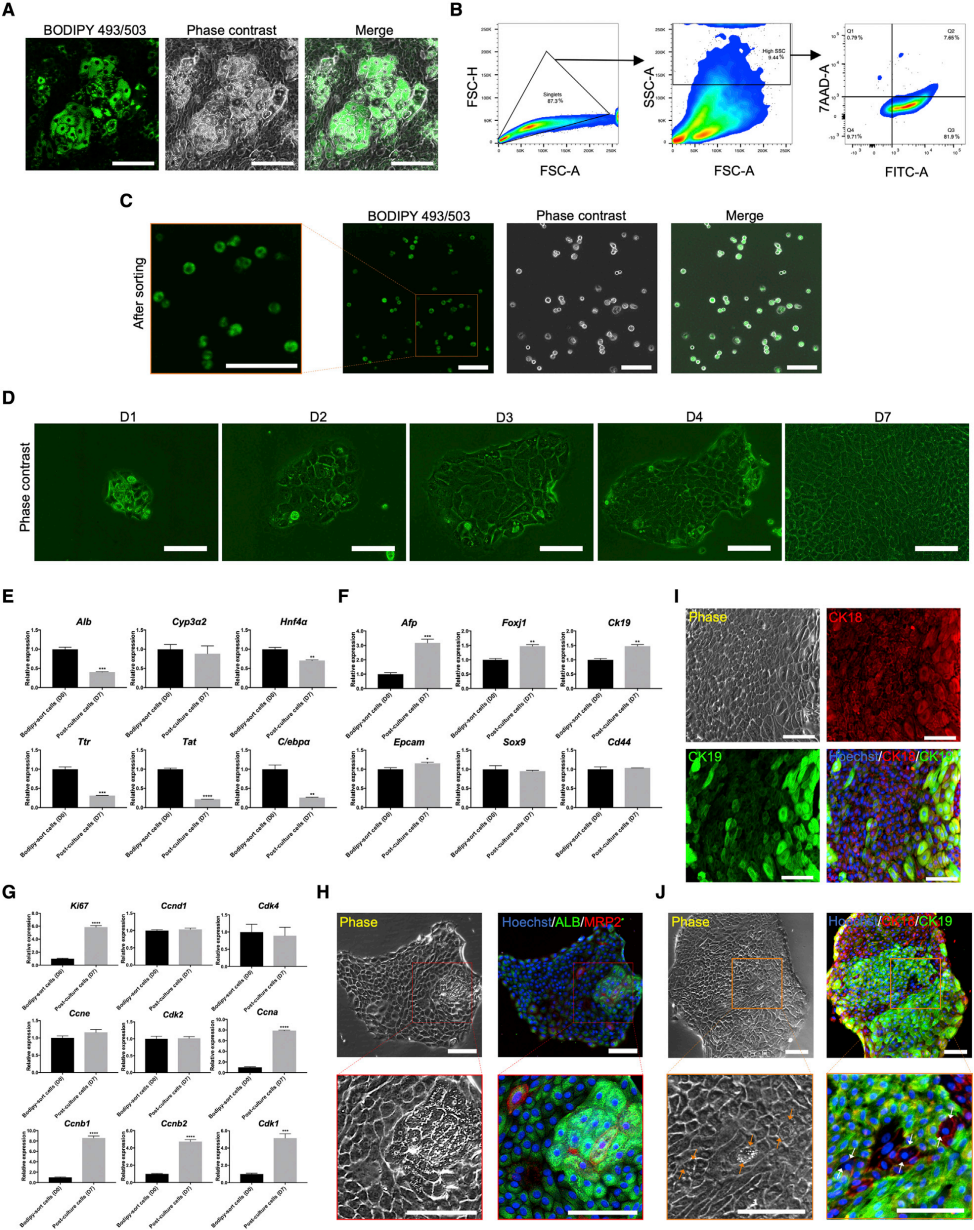
Spontaneously mature hepatocytes can re-differentiate into fully functional small cells (A) Lipid droplets were labeled by BODIPY 493/503. (B) Method used for isolating LDCs (in Q3). FITC, fluorescein isothiocyanate (used to detect BODIPY 493/503). (C) Verification of sorted LDCs by re-confirming the fluorescence signal. (D) Phase-contrast images of cultured sorted cells showing that LDCs re-entered proliferation with the disappearance of lipid droplets. (E) MH-related gene expression in freshly sorted LDCs and those cultured for 7 days. (F) LPC-specific gene expression in freshly sorted LDCs and those cultured for 7 days. (G) Cell-cycle-associated gene expression in freshly sorted LDCs and those cultured for 7 days. (H) Immunofluorescence staining of MH markers ALB and MRP2 in cultured sorted cells. (I) Immunofluorescence staining of cholangiocyte markers CK18 and CK19 in sorted cells that were induced into spontaneous cholangiocytic maturation. (J) Immunofluorescence staining of sorted cells that were induced into spontaneous cholangiocytic maturation, showing no CK19 expression in pre-existing LDCs after induction. The arrows denote LDCs. The data are expressed as the mean ± SD (n = 3 independent experiments), ^∗^p < 0.05; ^∗∗^p < 0.01; ^∗∗∗^p < 0.001; ^∗∗∗∗^p < 0.0001. Scale bars, 100 μm. See also Figure S5.

After passage, LDCs regained their remarkable proliferative activity, accompanied by the disappearance of lipid droplets (Figure S5A). Subsequently, proliferating cells spontaneously re-differentiated into MHs during culture, together with recurrence of lipid droplets and enhanced synthetic activity (Figure S5B). These phenomena suggested the interconvertibility between small cells and LDCs. To explore this conversion relationship more precisely, we attempted to sort LDCs using BODIPY 493/503. Lipid droplets incorporated BODIPY 493/503 (Figure 5A), and FACS was performed when a sufficient number of LDCs appeared. Due to abundant intracellular lipid droplets, a high side scatter area was gated as a candidate, and cells with high fluorescence were sorted subsequently (Figures 5B and S5C). After sorting, the identity of LDCs was re-confirmed by the presence of fluorescence in cell plasma (Figure 5C), and lipid droplets could be easily observed in sorted cells after 1 day in culture (Figure 5D). Consistent with the above results, sorted LDCs resumed significant proliferation accompanied by disappearance of lipid droplets during culture with YAC (Figure 5D). Compared with freshly sorted LDCs, MH-related genes were downregulated and LPC-associated genes were upregulated in cells cultured for 7 days (Figures 5E and 5F). Furthermore, expression of LPC markers CD44 and EPCAM in LDC-derived cells was increased with losing lipid droplets (Figure S5D). These results indicated that LDCs de-differentiated from differentiated hepatocytes into LPCs. Moreover, a cell-cycle-related gene analysis showed that G2/M phase-related genes, such as *Ccna*, *Ccnb1*, *Ccnb2*, and *Cdk1*, were upregulated after culture (Figure 5G), suggesting that LDCs retained proliferative potential but could not divide because of contact inhibition.

To verify whether LDC-derived small cells still reserved the potential for bipotent differentiation, we induced them to differentiate spontaneously using the methods mentioned above. After a long-term culture, LDCs recurred in the small-cell cluster (Figure S5E), accompanied by upregulated expression of MH markers ALB and MRP2 (Figure 5H). Besides, we removed YAC when small cells had proliferated to a considerable number. Some hypertrophic cells emerged subsequently (Figure S5F), which expressed the cholangiocyte markers CK18 and CK19 (Figure 5I). Consequently, we believe that LDCs could dedifferentiate into functional LPCs. Interestingly, some emerged LDCs did not change their morphological characteristics significantly after withdrawing YAC (Figure S5G) with expressing CK18, but no CK19 (Figures 5J and S5H), indicating that these LDCs were not affected by YAC withdrawal.

Noticeably, we confirmed that LDCs in the initial stage of hepatocytic differentiation could convert into small cells; however, whether this transformation process is restricted to certain stages is unclear.

## Discussion

The findings in our study propose new views on the origin of YAC-induced proliferative LPCs (small cells), and taking advantage of their proliferative properties under YAC stimulation, we purified LPCs and studied their differentiation patterns.

Currently, centrifugation remains the standard method for isolating fresh hepatocytes from rats (Zhang et al., 2016). Despite applying density gradient centrifugation, a non-negligible number of NPCs remain among the purified hepatocytes (Smedsrod and Pertoft, 1985). MHs, the source of CLiPs, were also obtained through centrifugation (Katsuda et al., 2017); therefore, it is probable that LPCs, which are similar in size to NPCs, contaminated the hepatocyte fraction and proliferated under YAC stimulation. Moreover, cells isolated from the supernatant, in which few MHs exist (Chen et al., 2007), were more likely to form small-cell colonies, suggesting that small cells originate from cells other than MHs. SHM is a selective medium for hepatocyte-progenitor cells (Chen et al., 2007), and we prefer that small cells are resident LPCs selected by SHM, which respond to YAC stimulation for further proliferation.

In recent years, small-molecule compounds have been proven to regulate different aspects of cell metabolism. YAC application, in combination or alone, has often been shown to regulate the cell proliferation status, such as maintaining embryonic stem cell self-renewal (Tsutsui et al., 2011), re-activating cardiomyocyte proliferation (Fan et al., 2018), and sustaining hepatoblast multiplication (Lv et al., 2015). Particularly, YAC was reported to be crucial for the long-term culture of hepatocyte-derived proliferative duct-like cells, a type of LPCs (Wu et al., 2017). We believe that YAC plays a similar role in small cells, facilitating the expansion of resident cells by inhibiting ROCK, TGFβR1, and GSK3, which are closely related to cell proliferation. Remarkably, at least two reports mentioned that modified YAC compound also reverses human MHs to proliferative LPCs (Katsuda et al., 2019; Kim et al., 2019); however, we suggest that the precise mechanism underlying the actions of YAC should be evaluated.

MH hypertrophy and hyperplasia are considered the main contributors to liver reconstruction under normal physiological conditions (Miyaoka et al., 2012). However, LPCs that are rarely observed in the normal liver play a crucial role in maintaining hepatocyte homeostasis during chronic liver injury (Espanol-Suner et al., 2012; Tarlow et al., 2014). The origin of LPCs remains highly controversial, and the potential candidate cells include dormant precursor cells (Theise et al., 1999), adult cholangiocytes (Espanol-Suner et al., 2012), hepatocytes (Tarlow et al., 2014), and metaplastic hepatic stellate cells (Kordes et al., 2007, 2014). It is also suggested that the liver has a flexible system of regeneration involving multiple cells, rather than a single type of LPC (Kuwahara et al., 2008). Although various molecular markers, such as AFP, CD133, EPCAM, and CK19, have been identified to describe LPCs, they may express distinct markers based on different origins or growth stages (Miyajima et al., 2014), and this uncertainty brought great difficulties to the identification of primary LPCs. For example, lineage tracing pointed out that SOX9^+^ cholangiocytes are LPC candidates and can convert into hepatocytes (Furuyama et al., 2011), whereas the latest study on this subject repudiated this conclusion after eliminating the interference of a handful of SOX9^+^ hepatocytes (He et al., 2017). Given the cell morphology and the profile of marker expression (CD44^+^/EPCAM^+^/AFP^+^), we believe that the origin of the YAC-induced proliferative small cells is hepatocytic progenitors (Mitaka, 2010); nonetheless, the absence of CK19 expression in small cells was not consistent with the results of the previous report (Mitaka, 2010). This difference in markers may also be explained by the above-mentioned theory that cells are at a variety of differentiation stages (Miyajima et al., 2014). The isolating and purifying method we reported may be an alternative solution for obtaining LPCs, as it avoids deviations caused by marker uncertainty.

**Figure 6:**
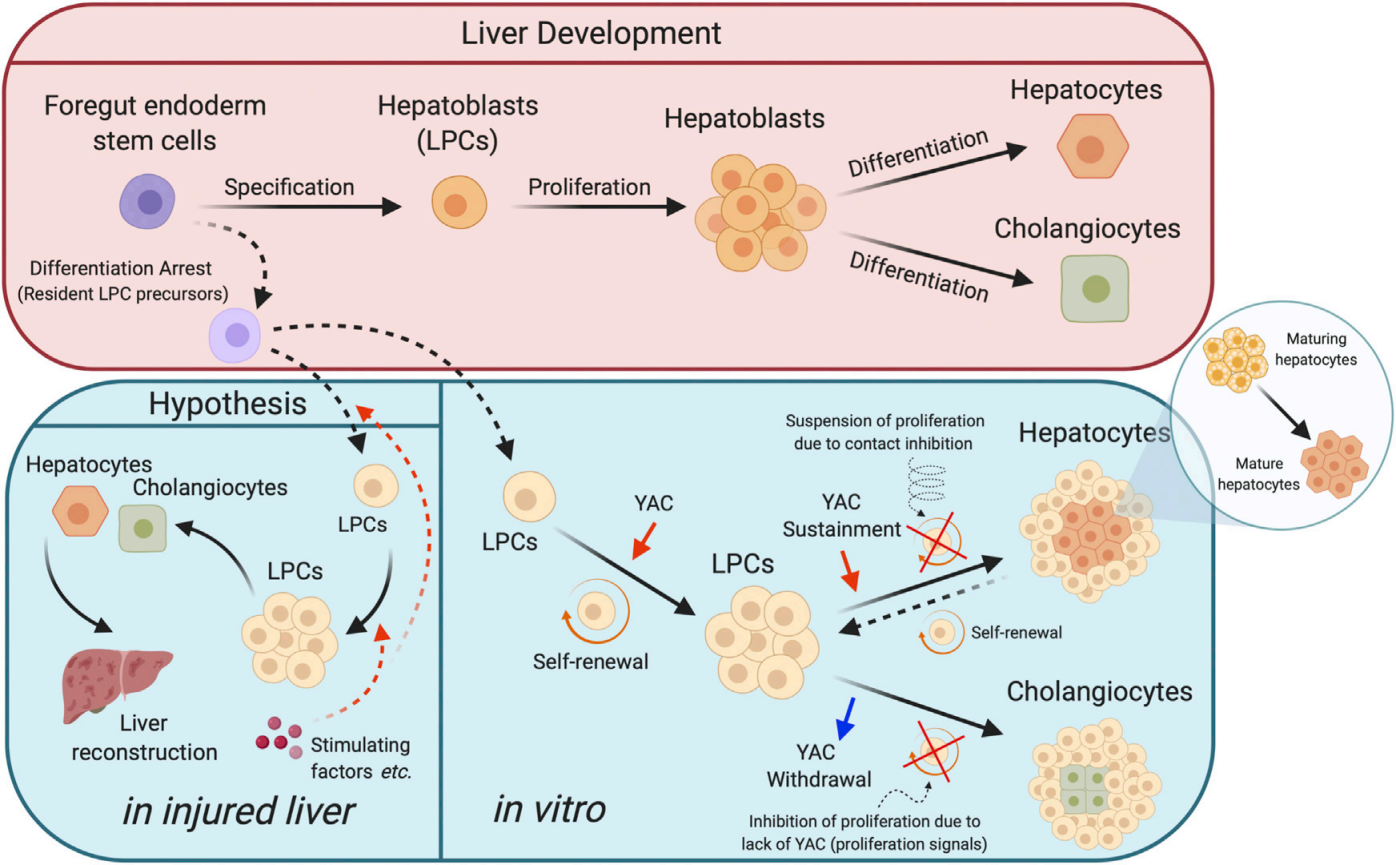
Schematic model of the developmental process of the liver, the transformation characteristics of liver progenitor cells (LPCs) *in vitro,* and the hypothesis of the protective mechanisms of LPCs against liver injury Although it is not clear why LPCs cannot be directly observed in the normal liver, the current evidence proves their existence, perhaps in the form of their precursors. In *in vitro* culture, by responding to changes in proliferation signals (the presence or absence of YAC, the percentage of cell confluence, etc.), LPCs can regulate and transform spontaneously between proliferation and differentiation statuses. Based on these data, we hypothesize that when liver injury occurs, LPCs are very likely to undergo regulation of their proliferation and differentiation with the same mechanism (the *in vitro* results we reported here) to reconstruct the liver. Created using BioRender.com.

Similar to the report that hepatocytic progenitors matured by interacting with NPCs (Mitaka et al., 1999), we found that small cells can mature spontaneously. Cell-cycle proteins are reported playing a role in enforcing pluripotency, and the knockdown of specific cyclins or CDKs results in the loss of the pluripotent state and triggers the differentiation of embryonic stem cells (Liu et al., 2019a). Based on the same logic, the decreased cyclins and CDK expression caused by growth arrest probably facilitates the spontaneous maturation of small cells. After release from contact inhibition, a rebound in the cell-cycle-related protein expression may cause the dedifferentiation of certain cells into proliferative cells during the initial stage of differentiation. To our surprise, despite the observation that YAC withdrawal also created a circumstance of suspended cell proliferation, it resulted in proliferated cells differentiating into cholangiocytes, rather than MHs. Such evidence indicates that small-cell differentiation is closely related to their proliferation status; however, this association cannot be solely attributed to whether the cells can continue to proliferate. Based on the results of our *in vitro* experiments, we hypothesized that small cells are resident hepatoblast precursors and play the following role in liver regeneration: when liver injury occurs, they first proliferate in large quantities and then gradually mature into functional hepatocytes (Miyajima et al., 2014) (Figure 6).

The capacity of small cells to proliferate abundantly *in vitro*, maintain bipotent differentiation capacity, and mature spontaneously indicates that they are a great tool for studying liver regeneration. The applicability of small cells to humans should be examined, and, if feasible, it is most likely to provide a new approach for the treatment of chronic liver disease, especially cirrhosis.

### Limitations of the study

Although SHM is considered as an LPC-selective medium, the possibility that emerging LPCs are cells reprogrammed by SHM cannot be excluded, and SHM contains complex components, whether the pro-proliferation effect of YAC requires the cooperation of some of these substances is unknown.

## Experimental procedures

### Animals

Adult male Sprague-Dawley rats (Japan SLC) with a body weight of 400 to 450 g were used for all experiments except lineage tracing (the Rosa26-LSL-tdTomato rat). The Animal Care and Use Committees of Hokkaido University approved the experimental protocol and animal care.

### Isolation of MHs and LPCs

Whole liver cells were obtained from rats using a method we reported previously (Fu et al., 2018), and the centrifugation strategy used for isolating MHs and LPCs was in accordance with other procedures (Chen et al., 2007; Seglen, 1976). In brief, after full enzymatic digestion, the cell suspension was first centrifugated for 10 min at 600 × *g*, and then the pellet was resuspended and centrifugated for 1 min at 60 × *g* twice, followed by mixing cells and Percoll, and centrifuging for 10 min at 60 × *g* to isolate MHs (see the supplemental experimental procedures). For isolating LPCs, the supernatant from the above-mentioned 60 × *g* centrifugation was collected and centrifuged at 50 × *g* for 5 min, then the pellet was resuspended and centrifugated for 5 min at 150 × *g* twice, followed by a final centrifugation at 50 × *g* for 5 min (see the supplemental experimental procedures).

### Cell culture strategy

After isolating MHs and LPCs, we cultured cells in basal medium or under YAC stimulation to evaluate their effects on promoting the proliferation of small cells. When a sufficient amount of small cells appeared in YAC-treated MHs, FACS was performed to purify small cells. To verify the bipotency of small cells, purified cells were induced to hepatocytes and cholangiocytes (Kamiya et al., 2002; Katsuda et al., 2017). Small cells also showed the ability to differentiate spontaneously after proliferation, and we controlled the direction of spontaneous differentiation by adding or withdrawing YAC. The concrete steps of the above-mentioned experimental procedures and related analysis are described in the supplemental experimental procedures, and Tables S1 and S2.

## Author contributions

Conceptualization, Q.F. and S.O.; Methodology, Q.F., G.S., and S.O.; Validation, Q.F.; Formal analysis, Q.F.; Investigation, Q.F.; Resources, Q.F.; Data Curation, Q.F.; Writing-Original Draft, Q.F.; Writing-Review & Editing, S.O.; Visualization, Q.F.; Supervision, N.S.; Project administration, S.O.; Funding acquisition, N.S.

## Conflict of interests

The authors declare no competing interests.

## Supplemental information

Document S1. Figures S1–S5, Tables S1, S2, and Supplemental experimental procedures

Document S2. Article plus supplemental information
