# Supplementary material for: Small-molecule inhibitor cocktail promotes the proliferation of pre-existing liver progenitor cells
Source: Stem Cell Reports. 2022 Jun 30;17(7):1589–603. doi: 10.1016/j.stemcr.2022.05.023 (PMC9287679; doi:10.1016/j.stemcr.2022.05.023)
Supplement: Document S1. Figures S1–S5, Tables S1, S2, and Supplemental experimental procedures [file mmc1.pdf]

Stem Cell Reports, Volume *17*

Supplemental Information

Small-molecule inhibitor cocktail promotes the proliferation of pre-existing liver progenitor cells

Qingjie Fu, Shunsuke Ohnishi, Goki Suda, and Naoya Sakamoto

Figure S1

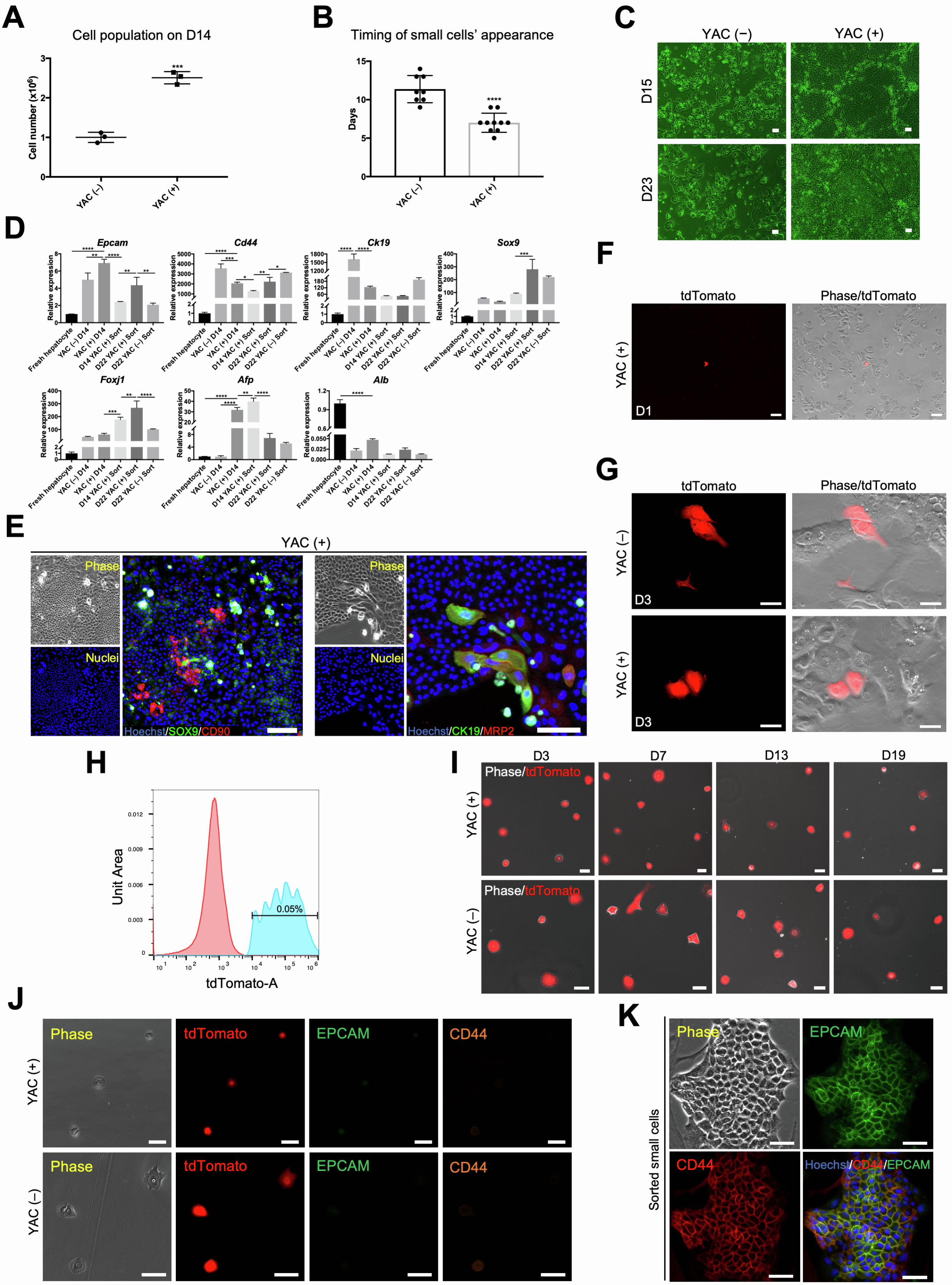
A
Cell population on D14
Cell number (x10^6)
YAC (–)
YAC (+)
B
Timing of small cells' appearance
Days
C
YAC (–)
YAC (+)
D15
D23
D
Epcam
Cd44
Ck19
Sox9
Foxj1
Afp
Alb
Relative expression
Fresh hepatocyte
YAC (–) D14
YAC (+) D14
D14 YAC (+) Sort
D22 YAC (+) Sort
D22 YAC (–) Sort
E
YAC (+)
Phase
Nuclei
Hoechst/SOX9/CD90
Hoechst/CK19/MRP2
F
tdTomato
Phase/tdTomato
D1
G
D3
H
Unit Area
0.05%
tdTomato-A
I
D3
D7
D13
D19
Phase/tdTomato
J
Phase
tdTomato
EPCAM
CD44
K
Sorted small cells
Hoechst/CD44/EPCAM

Figure S1. Characterization of YAC-treated cells (related to Figure 1)

(A) The number of cells on D14 of culture. The data are expressed as the mean ± SD (n = 3 independent experiments), $^{***}P < 0.001$. YAC (–), cells cultured without YAC; YAC (+), cells cultured under YAC stimulation. (B) Time points of the first appearance of small cells. The data are expressed as the mean ± SD (YAC (–), n=8 independent experiments, YAC (+), n=10 independent experiments), $^{****}P <$ 0.0001. (C) Phase-contrast images of cells treated with or without YAC in long-term culture. Scale bars, 100 μm. (D) Gene expression of LPC markers *Epcam*, *Cd44*, *CK19*, *Sox9*, *Foxj1*, and *Afp*, and of the MH marker *Alb* in fresh hepatocytes, YAC (–) cells, YAC (+) cells, YAC (–) Sort cells, and YAC (+) Sort cells. The data are expressed as the mean ± SD (n = 3 independent experiments), $^{*}P < 0.05$, $^{**}P < 0.01$, $^{***}P < 0.001$, $^{****}P < 0.0001$. LPC, liver progenitor cell; MH, mature hepatocyte; YAC (–) Sort cells, small cells sorted from YAC (–) cells; YAC (+) Sort cells, small cells sorted from YAC (+) cells. (E) Expression of LPC markers CD90 and SOX9 in small cells, and of the cholangiocyte marker (also an LPC marker) CK19 and the MH marker MRP2 in large cells. Scale bars, 100 μm. (F) Phase-contrast and fluorescence images of tdTomato^{+} MHs on D1. Scale bars, 100 μm.

(G) Phase-contrast and fluorescence images of tdTomato$^+$ MH-derived proliferative cells cultured with or without YAC on D3. Scale bars, 50 μm. (H) Gating strategy for tdTomato$^+$ MHs and ratio of tdTomato$^+$ MHs when performing FACS. (I) Images of sorted tdTomato$^+$ MHs cultured with or without YAC over time. Scale bars, 100 μm. (J) Expression of LPC markers CD44 and EPCAM in sorted tdTomato$^+$ MHs cultured with or without YAC on D21. Scale bars, 100 μm. (K) Expression of LPC markers CD44 and EPCAM in small cells sorted from Rosa26-LSL-tdTomato rat MHs cultured with YAC. Scale bars, 100 μm. The detailed sorting strategy is introduced in Figure 2A.

Figure S2

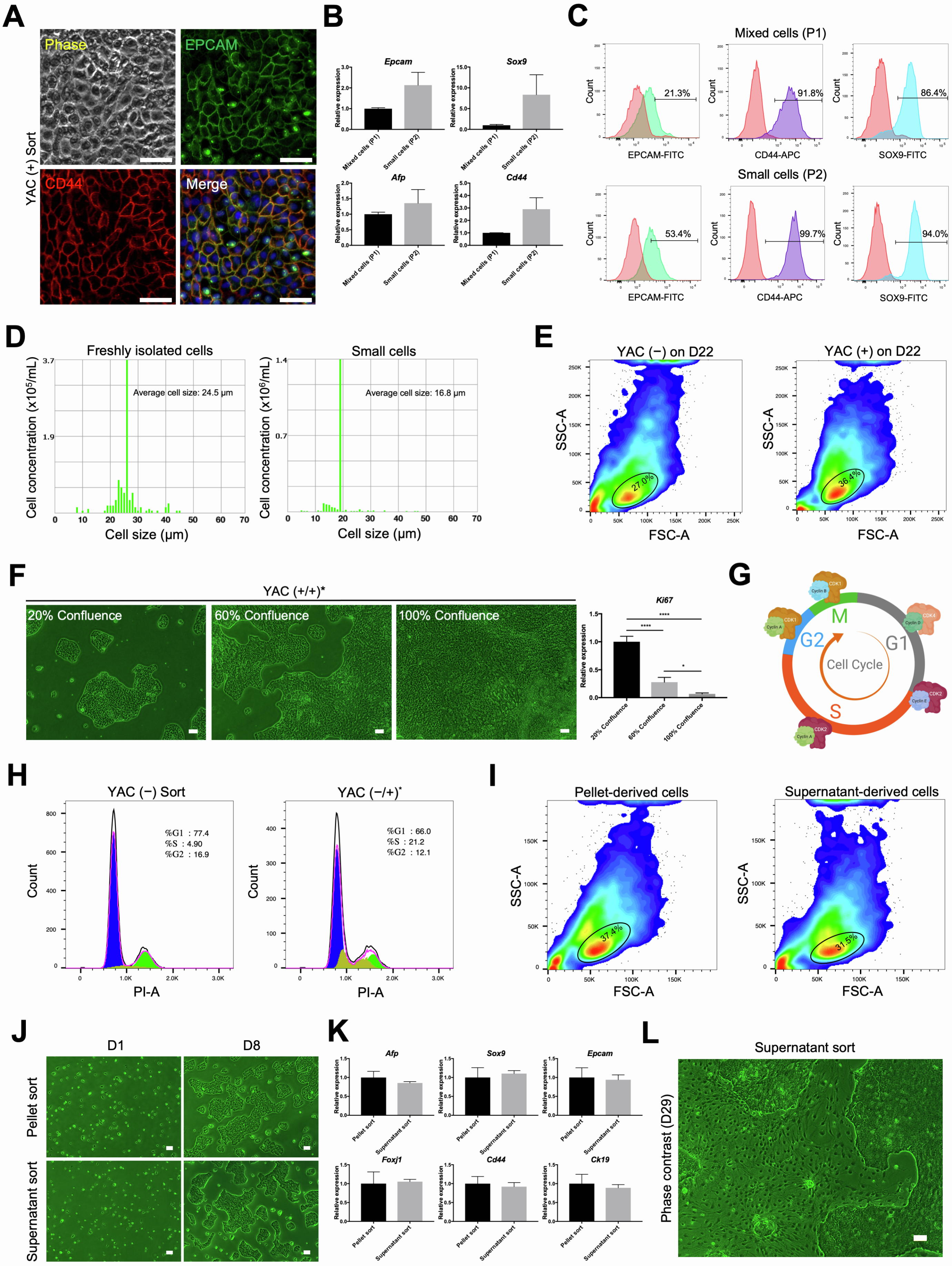
A
Phase
EPCAM
CD44
Merge
YAC (+) Sort
B
Epcam
Sox9
Afp
Cd44
Relative expression
Mixed cells (P1)
Small cells (P2)
C
Mixed cells (P1)
Small cells (P2)
Count
21.3%
91.8%
86.4%
53.4%
99.7%
94.0%
EPCAM-FITC
CD44-APC
SOX9-FITC
D
Freshly isolated cells
Small cells
Average cell size: 24.5 μm
Average cell size: 16.8 μm
Cell concentration (x10⁵/mL)
Cell concentration (x10⁶/mL)
Cell size (μm)
E
YAC (−) on D22
YAC (+) on D22
SSC-A
FSC-A
27.0%
36.4%
F
YAC (+/+)*
20% Confluence
60% Confluence
100% Confluence
Ki67
Relative expression
G
M
G1
G2
S
Cell Cycle
CDK1
Cyclin B
Cyclin A
CDK4
Cyclin D
CDK2
Cyclin E
H
YAC (−) Sort
YAC (−/+)*
%G1 : 77.4
%S : 4.90
%G2 : 16.9
%G1 : 66.0
%S : 21.2
%G2 : 12.1
Count
PI-A
I
Pellet-derived cells
Supernatant-derived cells
37.4%
31.5%
SSC-A
FSC-A
J
D1
D8
Pellet sort
Supernatant sort
K
Afp
Sox9
Epcam
Foxj1
Cd44
Ck19
Relative expression
Pellet sort
Supernatant sort
L
Supernatant sort
Phase contrast (D29)

Figure S2. Characterization of YAC-induced proliferative small cells (related to Figure 2)

(A) Expression of LPC markers EPCAM and CD44 in YAC (+) Sort cells. Scale bars, 100 μm. (B) Expression of the LPC-related genes *Epcam*, *Sox9*, *Afp*, and *Cd44* in sorted small cells (P2 cells) and in a cell mixture (P1 cells). The data are expressed as the mean ± SD (n = 3 independent experiments). See also Figure 2A. (C) Expression of LPC markers EPCAM, CD44 and SOX9 in sorted P1 cells and P2 cells. (D) Size of fresh MHs and sorted small cells. Cell counting and the measurement of cell size were performed using a Luna Automated Cell Counter. (E) Phase-contrast images of cultured YAC (+/+)* cells at different confluence levels and the corresponding gene expression of *Ki67.* The data are expressed as the mean ± SD (n = 3), $^{*}P < 0.05$, $^{****}P < 0.0001$. YAC (+/+)* cells, YAC (+) Sort cells cultured with YAC. (F) Phase-contrast images of cultured YAC (+/+)* cells at different confluence levels and the corresponding gene expression of *Ki67.* The data are expressed as the mean ± SD (n = 3 independent experiments), $^{*}P < 0.05$, $^{****}P < 0.0001$. YAC (+/+)* cells, YAC (+) Sort cells cultured with YAC. (G) Schematic representation of the cell cycle, showing the corresponding cyclins and cyclin-dependent kinases (CDKs) at each stage. (H) Cell cycle analysis of YAC (–)

Sort cells and YAC (–/+)* cells. YAC (–/+)* cells were treated with YAC until 30% confluence. PI, propidium iodide; YAC (–/+)* cells, YAC (–) Sort cells cultured with YAC. (I) Ratio of small cells among pellet-derived cells and supernatant-derived cells cultured with YAC for 14 days. (J) Phase-contrast images showing the proliferation of small cells sorted from YAC-treated pellet-derived cells and supernatant-derived cells under YAC stimulation. Scale bars, 100 μm. (K) Expression of LPC-related genes *Afp, Sox9, Epcam, Foxj1, Cd44* and *Ck19* in pellet-derived small cells and supernatant-derived small cells. The data are expressed as the mean ± SD (*n* = 3 independent experiments). (L) The phase-contrast image of cultured small cells sorted from YAC-treated supernatant-derived cells on D29, showing the appearance of other types of cells. Scale bar, 100 μm.

Figure S3

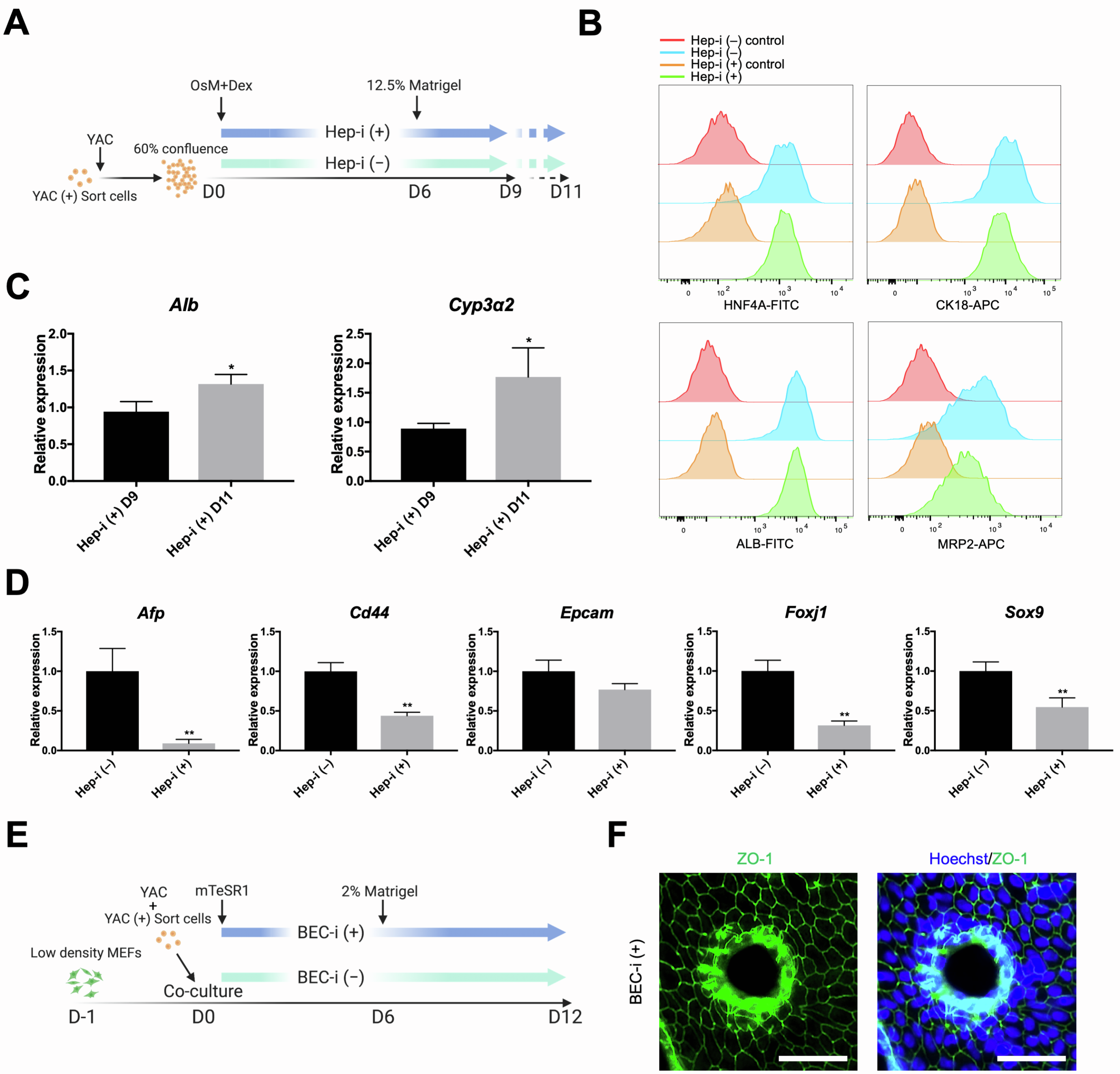

A
OsM+Dex
12.5% Matrigel
YAC
60% confluence
Hep-i (+)
Hep-i (−)
YAC (+) Sort cells
D0
D6
D9
D11
B
Hep-i (−) control
Hep-i (−)
Hep-i (+) control
Hep-i (+)
HNF4A-FITC
CK18-APC
ALB-FITC
MRP2-APC
C
Alb
Cyp3α2
Relative expression
Hep-i (+) D9
Hep-i (+) D11
D
Afp
Cd44
Epcam
Foxj1
Sox9
Relative expression
Hep-i (−)
Hep-i (+)
E
YAC
+
YAC (+) Sort cells
mTeSR1
2% Matrigel
Low density MEFs
BEC-i (+)
BEC-i (−)
Co-culture
D-1
D0
D6
D12
F
ZO-1
Hoechst/ZO-1
BEC-i (+)

Figure S3. Characterization of induced hepatocytes and cholangiocytes derived from small cells (related to Figure 3)

(A) Schematic representation of hepatocytic induction. Hep-i (–), YAC (+) Sort cells cultured with YAC alone; Hep-i (+), YAC (+) Sort cells cultured under hepatic induction. (B) Expression of MH markers ALB, CK18, HNF4A, and MRP2 in Hep-i (–) cells and Hep-i (+) cells, as determined by flow cytometry. (C) Gene expression of the MH functional markers *Alb* and *Cyp3α2* in Hep-i (+) cells on D9 and D11. The data are expressed as the mean ± SD (n = 3 independent experiments), $^{*}P < 0.05$. (D) Gene expression of LPC markers *Afp*, *Cd44*, *Epcam*, *Foxj1*, and *Sox9* in Hep-i (–) and Hep-i (+) cells on D9. The data are expressed as the mean ± SD (n = 3 independent experiments), $^{**}P < 0.01$. (E) Schematic representation of cholangiocytic induction. MEF, mouse embryonic fibroblast; BEC-i (–), YAC (+) Sort cells cultured without cholangiocytic induction; BEC-i (+), YAC (+) Sort cells cultured under cholangiocytic induction. (F) Expression of the tight junction marker ZO-1 in BEC-i (+) cells, showing a typical bile duct structure. Scale bars, 50 μm.

Figure S4

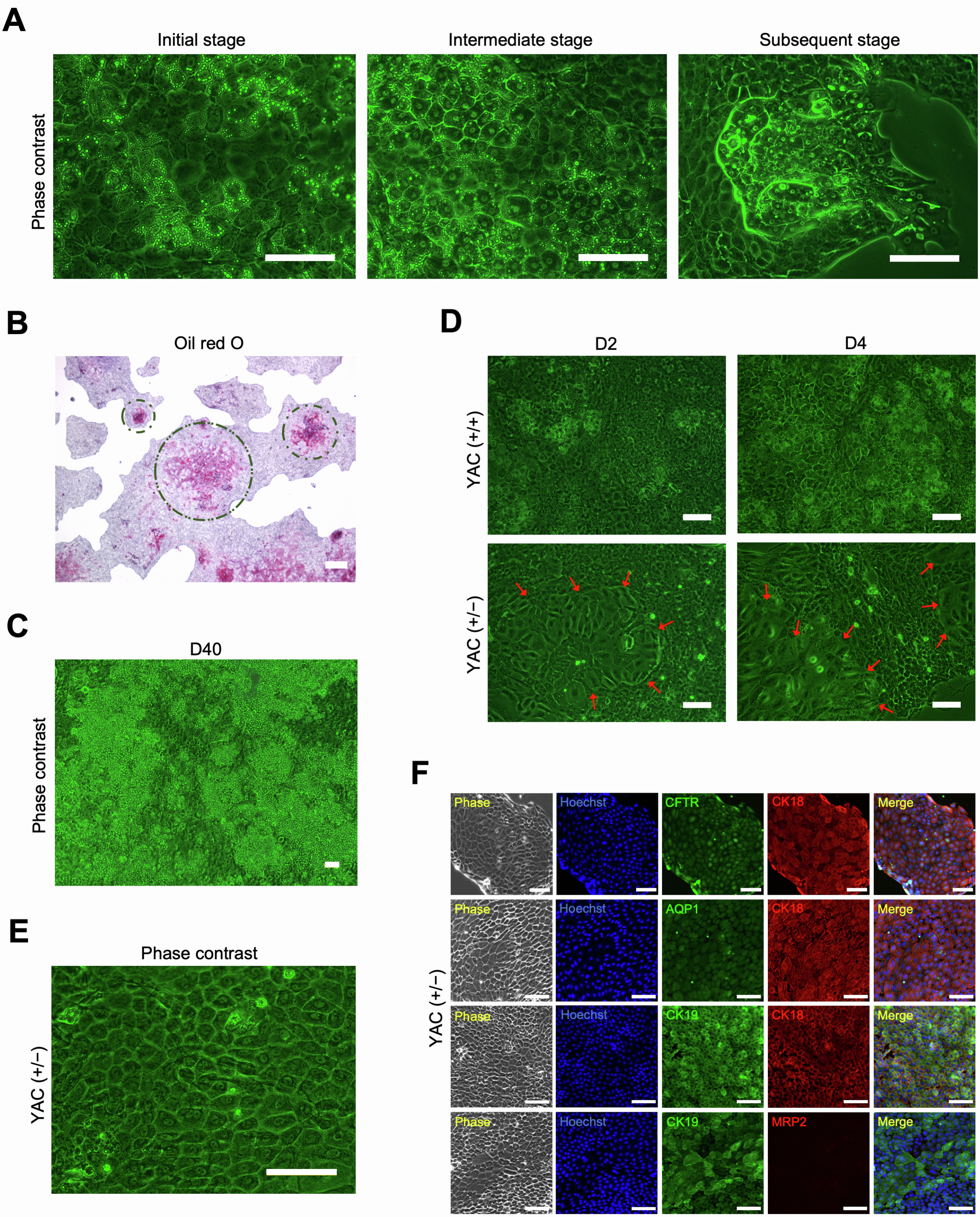

A
Initial stage
Intermediate stage
Subsequent stage
Phase contrast
B
Oil red O
C
D40
Phase contrast
D
D2
D4
YAC (+/+)
YAC (+/−)
E
Phase contrast
YAC (+/−)
F
YAC (+/−)
Phase
Hoechst
CFTR
CK18
Merge
AQP1
CK19
MRP2

Figure S4. Bidirectional spontaneous differentiation of small cells (related to Figure 4)

(A) Representative morphology of cells in the process of spontaneous differentiation to MHs. Scale bars, 100 μm. (B) Identification of lipid droplets in YAC-treated sorted small cells by oil red O staining. The dark-green closed loops indicate the position of LDCs in cell clusters. Scale bar, 300 μm. LDCs, cells containing lipid droplets. (C) The phase-contrast image showing the emergence of abundant lipid-droplet-containing cells in long-term culture. Scale bar, 100 μm. (D) Phase-contrast images of small cells treated with or without YAC withdrawal. The arrows indicate morphological changes in small cell. Scale bars, 100 μm. YAC (+/−), withdrawal of YAC from proliferated sorted small cells; YAC (+/+), maintenance of YAC in cultures of sorted small cells. (E) The phase-contrast image of cells with a low nucleus-to-cytoplasm ratio that appeared after YAC withdrawal. Scale bar, 100 μm. (F) Immunofluorescence staining of cells with a low nucleus-to-cytoplasm ratio for the cholangiocyte markers CK18, CK19, AQP1, and CFTR, and the MH marker MRP2. Scale bars, 100 μm.

Figure S5

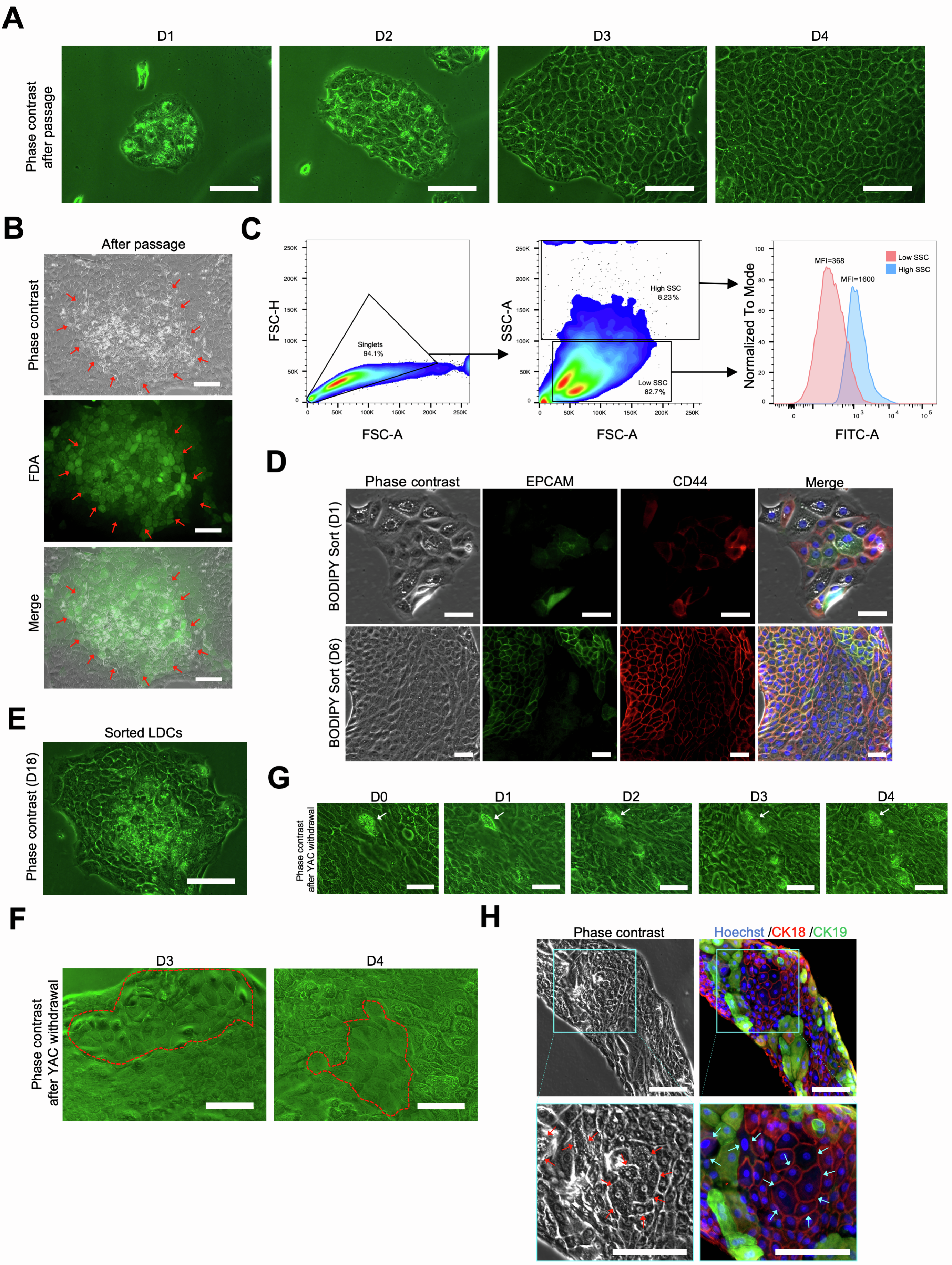

A
D1
D2
D3
D4
Phase contrast after passage
B
After passage
Phase contrast
FDA
Merge
C
FSC-H
FSC-A
Singlets 94.1%
SSC-A
High SSC 8.23%
Low SSC 82.7%
Normalized To Mode
FITC-A
MFI=368
MFI=1600
Low SSC
High SSC
D
Phase contrast
EPCAM
CD44
Merge
BODIPY Sort (D1)
BODIPY Sort (D6)
E
Sorted LDCs
Phase contrast (D18)
G
D0
D1
D2
D3
D4
Phase contrast after YAC withdrawal
F
D3
D4
Phase contrast after YAC withdrawal
H
Phase contrast
Hoechst /CK18 /CK19

Figure S5. Re-dedifferentiation of spontaneous mature hepatocytes into liver progenitor cells (related to Figure 5)

(A) Phase-contrast images of passaged LDCs exhibiting disappearance of lipid droplets after cells resumed proliferating. Scale bars, 100 μm. (B) Renewed spontaneous hepatocytic differentiation of small cells after passage, with enhanced synthetic capacity verified using an FDA assay. The arrows denote differentiated LDCs. Scale bars, 100 μm. FDA, fluorescein diacetate. (C) Gating strategy for LDC sorting. FITC, fluorescein isothiocyanate (used for the detection of BODIPY 493/503); MFI, mean fluorescence intensity. (D) Immunofluorescence staining of LPC markers EPCAM and CD44 in sorted LDCs that were cultured for 1 day and 6 days. Scale bars, 50 μm. (E) The phase-contrast image of sorted LDCs cultured for 18 days, showing the reappearance of lipid droplets. Scale bar, 100 μm. (F) Phase-contrast images of sorted LDCs that underwent YAC withdrawal. The closed loops indicate the position of morphologically altered cells. Scale bars, 100 μm. (G) Phase-contrast images of sorted cells that underwent YAC withdrawal, exhibiting the persistent presence of LDCs even after the elimination of YAC. The arrows denote LDCs. Scale bars, 100 μm. (H) Immunofluorescence

staining of cholangiocyte markers CK18 and CK19 in sorted cells that underwent YAC withdrawal. The arrows denote LDCs. Scale bars, 100 μm.

Table S1. Sequences of the primers used for qRT-PCR (related to Figures 2, 3, 4, 5, S1 and S2)

Gene	Forward primers (5′→3′)	Reverse primers (5′→3′)
Ae2	GACTCCTTTCCCTGTGTGGA	GAAGCATCCGCTCTTTCTTG
Afp	GCTGAACCCAGAGTACTGCAC	GACACGTCGTAGATGAACGTG
Alb	TGTCCCCAAAGAGTTTAAAGCTG	TCTTTATCTGCTTCTCCTTGTCTGG
Aqp1	CTGTGCGTTCTGGCTACCAC	GCACAGCAGAGCCAAATGAC
Aqp9	CTCAGTCCCAGGCTCTTCAC	TAAGACCTCCCAGGAAAGCA
C/ebp-α	GCCAAGAAGTCGGTGGATAA	CGGTCA TTGTCACTGGTCAA
Ccna	AACGATGAGCACGTCCCTACTGT	CAAGGATGGCCCGCATACTGTTA
Ccnb1	CCCTACCAAAACCTGTGGAC	CATCGGAGAAAGCCTGACAC
Ccnb2	TGGAGAGTGAAATACTGGAAGTCA	TGAGAAGCACACGATGGAAG
Ccnd1	TGCTTGGGAAGTTGTGTTGG	AATGCCATCACGGTCCCTAC
Ccne	GAAAATCAGACCGCCCAGAG	CGCTGCAGAAAGTGCTCATC
Cd44	GGCTTATCATCTTGGCATCC	CTGTTCCA TTGCCACTGTTG
Cdk1	GTACGGCAATCCGGGAAATC	GAGATACAGCCTGGAGTCCT
Cdk2	CATCAAGCTGGCTGACTTTGGA	GTGGAGTAGTACTTGCAGCCCAGAA

Cdk4	GGAGGCCTTTGAACATCCCA	ACTGGCGCATCAGATCCTTA
Cftr	GGTCATAGAGCAGGGCAATG	TGCACTTCTTCCTCCGTCTC
Ck18	TTCTGGGTTGACTGTGGAAGT	TGGTACTCTCCTCAATCTGCTG
Ck19	TTCCGGACCAAGTTTGAGAC	CCTCGTGGTTCTTCTTCAGG
Cyp3α1	TGGTAATAGACTTGAGAGAG	GGGCAGATATACATAAGGA
Cyp3α2	AGTAGTGACGATTCCAACATAT	TCAGAGGTATCTGTGTTTCCT
Epcam	TCGTGGTGGTGTTAGCAGTC	TCTGTGTATCTCACCCATCTCC
Foxj1	AGAACTGGGAACTGGGGACT	CTTCGGCTCTCGGAGTACAG
Grhl2	GTTCGATGCTCTGATGCTGA	GCAGCCCGTACTTCTCAGAC
Hnf4α	CTGCAGGCTCAAGAAGTGC	GGGAGGTGA TCTGCTGAGAC
Ki67	ATTTCAGTTCCGCCAATCC	GGCTTCCGTCTTCATACCTAAA
Mrp2	TGATCGGTTTCGTGAAGAGCT	ACGCACATTCCCAACACAAA
Sox9	TCCTAACGCCATCTTCAAGG	ACGTCTGTTTTGGGAGTGGT
Tat	ACCTCTGCTATGGGGCACT	ACTCCACGTCGTTCTCGAA T
Ttr	TCGTACTGGAAGGCTCTTGG	CCAGAGTCA TTGGCTGTGAA
β-actin	ATCTGGCACCACACCTTC	AGCCAGGTCCAGACGCA

Table S2. Key Resources Table (related to experimental procedures and supplemental experimental procedures)

REAGENT and RESOURCE	SOURCE	IDENTIFIER
**Antibodies**		
Mouse monoclonal anti-CD44	Cell Signaling	Cat#5640;<br>RRID:AB_10547133
Mouse monoclonal anti-CD90	BioLegend	Cat#202501;<br>RRID:AB_314013
Mouse monoclonal anti-CK18	Abcam	Cat#ab668;<br>RRID:AB_305647
Mouse monoclonal anti-MRP2	Abcam	Cat#ab3373;<br>RRID:AB_303751
Rabbit monoclonal anti-AFP	Abcam	Cat#ab213328;<br>RRID:AB_2884974
Rabbit monoclonal anti-EPCAM	Abcam	Cat#ab213500;<br>RRID:AB_2884975
Rabbit monoclonal anti-SOX9	Abcam	Cat#ab185230;<br>RRID:AB_2715497
Rabbit polyclonal anti-ALB	GeneTex	Cat#GTX102419;<br>RRID:AB_1949626
Rabbit polyclonal anti-AQP1	Sigma-Aldrich	Cat#SAB5200109;<br>RRID:AB_2884973
Rabbit polyclonal anti-AXIN2	Abcam	Cat#ab32197;<br>RRID:AB_2290204
Rabbit polyclonal anti-CFTR	Sigma-Aldrich	Cat#SAB4501942;<br>RRID:AB_10745324

Rabbit polyclonal anti-CK19	Novus	Cat#NB100-687;<br>RRID:AB_2265512
Rabbit polyclonal anti-HNF4A	Bioss	Cat#bs-3828R;<br>RRID:AB_10856932
Rabbit polyclonal anti-ZO-1	Thermo Fisher Scientific	Cat#61-7300;<br>RRID:AB_2533938
Goat anti-rabbit Alexa Fluor 488	Cell Signaling	Cat#4412S;<br>RRID:AB_1904025
Goat anti-mouse Alexa Fluor 647	Abcam	Cat#ab150115;<br>RRID:AB_2687948
Goat anti-mouse APC	BioLegend	Cat#405308;<br>RRID:AB_315011
**Bacterial and virus strains**		
AAV.TBG.PI.Cre.rBG	Addgene	Addgene viral prep # 107787-AAV8;<br>RRID:Addgene_107787
**Chemicals, Peptides, and Recombinant Proteins**		
4% paraformaldehyde (PFA)	Wako	Cat#163-20145
7-aminoactinomycin D (7-AAD)	BD Biosciences	Cat#559925;<br>RRID:AB_2869266
A83-01	Wako	Cat#035-24113
BODIPY 493/503	Invitrogen	Cat#D3922
Bovine serum albumin (BSA)	Wako	Cat#015-23871
CHIR99021	Axon Medchem	Cat#1386

Collagenase II	Worthington Biochemical	Cat#LS004176
Deoxyribonuclease I (DNase I)	Worthington Biochemical	Cat#LS002139
Dexamethasone (Dex)	Wako	Cat#041-18861
DMEM/F12	Gibco	Cat#11320-033
Epidermal growth factor (EGF)	Sigma-Aldrich	Cat#SRP3238
Ethylenediaminetetraacetic acid (EDTA)	Invitrogen	Cat#15575-038
Fluorescein diacetate (FDA)	Sigma-Aldrich	Cat#F7378
FxCycle^{TM} PI/RNase Staining Solution	Invitrogen	Cat#F10797
HEPES	Gibco BRL	Cat#11344-033
Hoechst 33342	Thermo Fisher Scientific	Cat#62249
Insulin-transferrin-serine (ITS)-X	Gibco	Cat#51500-056
Isopropanol	Wako	Cat#164-08335
l-ascorbic acid 2-phosphate	Wako	Cat#013-12061
Live cell imaging solution	Invitrogen	Cat#A14291DJ
l -proline	Sigma-Aldrich	Cat#P5607
Matrigel Matrix	Corning	Cat#356237
MEF culture medium	Cosmo Bio	Cat#MEF-M
Methanol	Wako	Cat#131-01826
mTeSR^{TM}1 Complete Kit	STEMCELL Technologies	Cat#85850
Nicotinamide	Sigma-Aldrich	Cat#N3376
Oil red O	Sigma-Aldrich	Cat#O0625

Oncostatin M (OsM)	Sigma-Aldrich	Cat#SRP3250
Penicillin-streptomycin	Wako	Cat#168-23191
Percoll	GE Healthcare	Cat#17-0891-02
Phenol red-free MEMα	Wako	Cat#134-17225
Secretin	Tocris Bioscience	Cat#1919
Trypsin/EDTA	Wako	Cat#208-17251
Y-27632	Wako	Cat#036-24023
**Critical Commercial Assays**		
Cell Counting Kit-8 (CCK-8)	Dojindo Laboratories	Cat#347-07621
Click-iT® Plus EdU Imaging Kit	Invitrogen	Cat#C10640
PAS Staining Kit	Sigma-Aldrich	Cat#395B-1KT
Platinum SYBR Green qPCR Mix	Invitrogen	Cat#11744-500
PrimeScript RT Reagent Kit with gDNA Eraser	Takara Bio	Cat#RR047A
Rat Albumin ELISA Kit	Bethyl	Cat#E111-125
RNeasy Mini Kit	Qiagen	Cat#74106
Urea Assay Kit	Abcam	Cat#ab83362
**Experimental Models: Cell Lines**		
Mouse: Embryonic fibroblasts (MEF)	Cosmo Bio	Cat#MEF-01C
**Experimental Models: Organisms/Strains**		
Sprague-Dawley *Rat*	Japan SLC	Slc:SD
Rosa26-LSL-tdTomato *Rat*	NBRP-Rat	NBRP-Rat NO.0734

**Oligonucleotides**		
Primers used for qRT–PCR, see Table S1	This paper	N/A
**Software and Algorithms**		
ImageJ	https://imagej.nih.gov/ij/	RRID:SCR_003070
FlowJo V10	FLOWJO, LLC	RRID:SCR_008520
Graph Pad Prism V7.0	GraphPad Software	RRID:SCR_002798

Supplemental Experimental Procedures

Isolation of MHs

Whole cells of the rat liver were obtained using a method reported previously by us [1], whereas the centrifugation strategy used for isolating MHs was in accordance with another procedure [2]. In brief, the liver was perfused with 60 mL of Ca^{2+}/Mg^{2+}-free Hank's Balanced Salt Solution (HBSS (–); Gibco) containing 1 mM ethylenediaminetetraacetic acid (EDTA; Invitrogen) at 18 mL/min via the portal vein after anesthesia, followed by perfusion with 200 mL of HBSS (–) supplemented with 100 U/mL of collagenase II (Worthington Biochemical) at 7.5 mL/min. Subsequently, we peeled the capsule of the well-digested liver with tweezers and performed further digestion with 120 mL of HBSS (–) containing 65 U/mL of collagenase II and 400 U/mL of deoxyribonuclease I (DNase I; Worthington Biochemical) at 37°C with shaking on a stirring plate at 70 rpm for 20 min. The obtained cell suspension was filled up to 160 mL with HBSS (–) and filtered twice, using first a 100-μm and then a 70-μm cell strainer (Falcon). Next, we split the filtrate four ways into 50-mL tubes and performed centrifugation for 10 min at 600×*g* at 4°C. The pellets were gathered into two tubes, then rinsed with 40 mL of

HBSS (–) containing 120 U/mL of DNase I in each tube and centrifugated for 1 min at 60×*g* at 4°C. This step was repeated once. Cells were collected into one tube and resuspended in 20 mL of HBSS (–) containing 120 U/mL of DNase I, then mixed with 100% Percoll (GE Healthcare; Prepared in advance with Percoll stock solution and 1.5 M NaCl solution at a ratio of 9:1) to a final concentration of 48.9% (v/v). Cells in the pellet obtained after centrifugation for 10 min at 60×*g* at 4°C were regarded as purified MHs and were used in follow-up experiments after being centrifuged twice at 60×*g* for 2 min and filtered once with a 40-μm cell strainer (Falcon). A Luna automated cell counter (Logos Biosystems) was used to perform the cell count and measure cell size.

Isolation of LPCs

The supernatant obtained after the first centrifugation at 60×*g* during the isolation of MHs was used to obtain LPCs [3]. First, the supernatant was centrifugated at 50×*g* for 5 min at 4°C, followed by the dissociation of the pellet by adding 40 mL of HBSS (–) containing 120 U/mL of DNase I in each tube and an additional centrifugation under the same conditions. Cells were collected and rinsed with 40 mL of HBSS (–) containing 120 U/mL of DNase I in each tube, followed by

centrifugation for 5 min at 150×*g* at 4°C. This step was repeated once, and then the cells were gathered into one tube and rinsed with 40 mL of HBSS (–) containing 120 U/mL of DNase I. After centrifugation at 50×*g* for 5 min at 4°C, we filtered cells with a 40-μm cell strainer and obtained crude LPCs mixed with other NPCs.

Cell Culture Models

Typically, cells were cultured in a humidified atmosphere of 95% air and 5% CO_2 at 37°C, while a hypoxic environment was set at 37°C, 5% O_2 and 5% CO_2. SHM was used as the basal medium for cell culture [3], *viz*, DMEM/F12 (Gibco) containing 2.4 g/L $NaHCO_3$ and L-glutamine, which was supplemented with 5 mM HEPES (Gibco BRL), 30 μg/mL of L-proline (Sigma-Aldrich), 0.05% bovine serum albumin (BSA; Wako), 10 ng/mL of epidermal growth factor (Sigma-Aldrich), 1% insulin-transferrin-serine (ITS)-X (Gibco), 10^{-7} M dexamethasone (Dex; Wako), 10 mM nicotinamide (Sigma-Aldrich), 1 mM L-ascorbic acid 2-phosphate (Wako), and 1% penicillin–streptomycin solution (Wako). YAC was formulated as follows [4]: 10 μM Y-27632 (Wako), 0.5 μM A83-01 (Wako), and 3 μM CHIR99021 (Axon Medchem).

To verify the real role of YAC, we set up a variety of culture models by adjusting the timing of the addition and withdrawal of YAC. Unless otherwise stated, the

corresponding medium was replaced every other day. A Luna Automated Cell Counter was used to perform cell counting. During each cell culture process, image acquisition was carried out using an IX70 Inverted Tissue Culture Microscope (Olympus) and a DS-L3 Digital Camera Controller (Nikon). Image processing and relevant calculations were achieved using the ImageJ software (https://imagej.nih.gov/ij/).

The primary MHs were seeded on collagen type 1-coated plates (Iwaki) at 1×10^4 cells/cm^2 and cultured in SHM with or without YAC, accompanied by a change of the medium 1 day after seeding and every 2 days thereafter.

To verify if YAC promoted small-cell proliferation, YAC (–) cells were cultured until small-cell clusters emerged; then, the medium was replaced with YAC-containing medium (YAC (–/+) cells), which was recorded as D0. Cell culture lasted for 10 days, and YAC (–) cells cultured without YAC in the same period (YAC (–/–) cells) were used as controls. For comparison, multiple identical cell clusters were continuously observed.

Cells sorted from YAC (–) cells (YAC (–) Sort cells) and YAC (+) cells (YAC (+) Sort cells) on D22 were used to further investigate the effects of YAC on small cells.

The day of sorting was recorded as D0, and the YAC (–) Sort cells were cultured in the presence of YAC (YAC (–/+)[*] cells) or continued to be cultured using SHM alone (YAC (–/–)[*] cells) for 10 days. Similarly, YAC (+) Sort cells were cultured in the same manner, namely, in the form of YAC (+/+)[*] cells and YAC (+/–)[*] cells.

To examine the origin of small cells, we treated MHs (pellet-derived cells) and LPCs (supernatant-derived cells) under YAC stimulation during a 14-day culture. Homologous cells cultured with SHM throughout the corresponding period were set as controls.

For the purpose of assessing whether YAC had any effect on already proliferated small cells, YAC (+) Sort cells were cultured in the presence of YAC (YAC (+/+) cells) until 80% confluence, followed by the withdrawal of YAC (YAC (+/–) cells) for 4 days. Cells that were always cultured in medium containing YAC were used as a control.

FACS for Small Cells and Cells Containing Lipid Droplets (LDCs)

The MHs or crude LPCs cultured with YAC could be sorted on D14, whereas the sorting of cells cultured without YAC stimulation was performed on D22. The cells were collected using 0.5% trypsin/EDTA (Wako) and resuspended in phenol-red-

free MEMα (Wako) supplemented with 1.5% fetal bovine serum (FBS; Sigma-Aldrich) after centrifugation for 5 min at 400 × *g*. The suspension was filtered using a 40-μm cell strainer and adjusted to a final concentration of 1 × 10^7 cells/mL. 7-Aminoactinomycin D solution (7-AAD, 2 μL/10^6 cells; BD Biosciences) was added to the suspension immediately before sorting. A 15 mL polypropylene tube containing 10 mL of SHM was used to collect the sorted cells.

Small cells were cultured with YAC until plenty of LDCs appeared. First, cells were incubated with 2 μg/mL of BODIPY 493/503 (Invitrogen) for 30 min at 37°C. The cells were then collected using 0.5% trypsin/EDTA and resuspended in phenol-red-free MEMα supplemented with 1.5% FBS after centrifugation for 5 min at 400 × *g*. The suspension was filtered using a 40-μm cell strainer, and 7-AAD (2 μL/10^6 cells) was added to the suspension immediately before sorting. A 15 mL polypropylene tube containing 10 mL of SHM supplemented with YAC was used to collect the sorted LDCs.

A FACS Aria III Cell Sorter (BD Biosciences) was used to perform FACS, and the data were analyzed using FlowJo software (FLOWJO, LLC). The collected cells were centrifuged for 5 min at 400×*g* at 4°C, followed by seeding on collagen

type 1-coated plates at 1.5×10^4 cells/cm^2.

***In vitro* lineage tracing of rat MHs**

AAV8-TBG-Cre was injected to a 16-week-old male Rosa26-LSL-tdTomato rat from the tail vein at 1.1×10^{12}GC. After 7 days, we sacrificed the rat and isolated MHs. On the other hand, we sorted tdTomato$^+$ MHs using an SH800 Cell Sorter (Sony Biotechnology) with 561nm laser. Unsorted MHs and sorted tdTomato$^+$ MHs were cultured with or without YAC using the method described above.

Hepatocytic Induction of Small Cells

Small cells sorted from YAC-treated cells were induced into MHs. The sorted cells were cultured with YAC until 50% cell confluence. Subsequently, for hepatocytic induction (Hep-i (+) cells), the culture medium was supplemented with 20 ng/mL oncostatin M (OsM; Sigma-Aldrich) and 10^{-6} M Dex [5] during a 6-day culture, with a change into fresh medium performed every other day. On D6, the cultured small cells were overlaid with the mixture of Matrigel (Corning) and the hepatic induction medium at a 1:7 ratio. The cell culture was continued for another 3 days, or 5 days with one replacement of the Matrigel mixture, to end the induction. Before various assays, the covered Matrigel had to be removed via gentle aspiration. Sorted small

cells that were cultured with YAC alone throughout the corresponding culture period served as the negative control (Hep-i (–) cells).

Cholangiocytic Induction of Small Cells

The cholangiocytic induction referred to a previous procedure [4]. Mitomycin C-treated mouse embryonic fibroblasts (MEFs; Cosmo Bio) were inoculated on collagen type 1-coated 6-well plates at 1×10^5 cells/well and cultured with MEF culture medium (Cosmo Bio). On the following day, cultured YAC (+) Sort small cells were harvested using trypsin/EDTA and cultured on pre-seeded MEFs at 1×10^6 cells/well with YAC and 5% FBS-supplemented medium for 24 h. Subsequently, cholangiocytic induction (BEC-i (+) cells) was initiated by replacing the medium with mTeSR™1 complete medium (STEMCELL Technologies) containing YAC. This process of induction lasted 6 days, and the medium was renewed every 2 days. Matrigel (2%) was added to the induction medium on D6, to complete the cholangiocytic induction, and the cells were cultured for an additional 6 days, with the medium being replaced every other day. After this 12-day induction, the cells were analyzed in various ways. As a negative control (BEC-i (–) cells), sorted small cells were cultured on MEFs only with YAC throughout the corresponding culture

period.

RNA Isolation and qRT–PCR

RNA of the isolated or cultured cells was extracted using an RNeasy Mini Kit (Qiagen) according to the manufacturer's instructions. Total RNA was reverse transcribed into cDNA using a PrimeScript RT Reagent Kit with gDNA Eraser (Takara Bio) in a Veriti 96-well Thermal Cycler (Applied Biosystems) using the following conditions: incubation at 37°C for 15 min and at 85°C for 15 s. qPCR was carried out in a total reaction volume of 25 μL containing 5 μL of template cDNA mixture, 12.5 μL of a Platinum SYBR Green PCR Mix (Invitrogen), and 2 μL of a 10 μM corresponding primer mixture. The PCR conditions on a Step One Plus Real Time PCR System (Applied Biosystems) included pre-denaturation at 95°C for 20 s, followed by 40 cycles of 95°C for 3 s and 60°C for 7 s. The relative expression was determined using the relative standard curve method and *β-actin* was used as an endogenous control. Table S1 shows the sequences of the primers used in this experiment.

Flow Cytometry Analysis

We harvested the cultured Hep-i (+) cells using 0.5% trypsin/EDTA and fixed them

with precooled (–30°C) methanol (Wako) for 10 min at room temperature (RT). The cells were then centrifuged and washed with phosphate-buffered saline (PBS; Gibco) at 400×*g* for 2 min. PBS containing 1% (w/v) BSA was used to block the cells for 30 min at RT. Next, the cells were incubated with primary antibodies against ALB (1:300; GeneTex), CK18 (1:250; Abcam), HNF4A (1:200; Bioss), and MRP2 (1:200; Abcam) for 1 h at RT, respectively. After washing twice with PBS at 400×*g* for 2 min each time, the cells were incubated with the following secondary antibodies, anti-rabbit Alexa Fluor 488 (1:800; Cell Signaling Technology) or anti-mouse APC (1:400; BioLegend), depending on the species in which the primary antibody was raised, for 30 min at RT in the dark, followed by a final wash in PBS at 400×*g* for 2 min. All prepared cells were resuspended in HBSS (–), filtered using a 40-μm strainer, and analyzed using a FACS Canto II Flow Cytometer (BD Biosciences).

Immunofluorescence Staining

The cultured cells were washed three times using HBSS (–) and then fixed in precooled (–30°C) methanol for 10 min at RT. After washing with HBSS (–), blocking was performed with PBS containing 1% (w/v) BSA for 30 min at RT.

Subsequently, the cells were incubated with primary antibodies for 1 h at RT, followed by incubation with secondary antibodies for 30 min at RT in the dark. All antibodies were prepared in PBS and were diluted as follows: AFP, CD90, CK18, CK19, CFTR, EPCAM, HNF4A, MRP2, and ZO-1, 1:100; ALB, AXIN2, AQP1, and SOX9, 1:200; anti-rabbit Alexa Fluor 488, and anti-mouse Alexa Fluor 647, 1:500. Finally, the nuclei were stained with Hoechst 33342 (1:800; Thermo Fisher Scientific) for 3 min at RT and washed with HBSS (–) for observation. A FluoView FV10i Confocal Laser Scanning Microscope (Olympus) was used to complete the image acquisition and analysis.

Cell Cycle Analysis

A part of YAC (–) Sort cells were fixed using precooled (–30°C) 70% ethanol and stored at –30°C till analyzing. The rest YAC (–) Sort cells were cultured with YAC and collected when achieving 30% confluence. Cultured cells were also fixed by 70% ethanol (–30°C). All cells were washed with PBS and then stained with FxCycle^{TM} PI/RNase Staining Solution (Invitrogen) for 30 min. Stained cells were analyzed using a FACS Canto II Flow Cytometer and the data was analyzed using the FlowJo software.

Oil Red O Staining

Lipid was detected in the cells by oil red O staining. The oil red O stock solution was prepared by dissolving 0.5 g of oil red O powder (Sigma-Aldrich) in 100 mL of isopropanol (Wako), and the oil red O working solution was formulated by mixing the oil red O stock solution with ddH_2O at a ratio of 3:2. Cultured YAC (+) sort small cells were washed with PBS and fixed with 4% paraformaldehyde (PFA) for 5 min at RT. Next, the cells were washed three times using PBS and rinsed with 60% isopropanol for 2 min. The freshly prepared oil red O working solution was filtered with filter paper (Advantec) and used to stain the cells for 15 min at RT, followed by rinsing with 60% isopropanol for 30 s. Nuclei were stained with hematoxylin (Sigma-Aldrich) for 90 s, and the cells were prepared for observation after washing with PBS. A BZ-9000 All-In-One Fluorescence Microscope (Keyence) was used to scan the samples.

PAS Staining

PAS staining was performed to determine glycogen using a PAS Staining System (Sigma-Aldrich). The cell preparation and staining strategy was according to the standard procedures provided by the manufacturer. Image acquisition was

performed using a BZ-9000 All-In-One Fluorescence Microscope.

BODIPY 493/503 Staining

BODIPY 493/503 was added to the culture medium at a final concentration of 2 μg/mL. After incubating for 30 min at 37°C, small cells were rinsed twice with Hank's Balanced Salt Solution (HBSS (+); Gibco) and then overlaid with a live-cell imaging solution (Invitrogen) for scanning. A FluoView FV10i Confocal Laser Scanning Microscope was used to acquire the images.

EdU Assay

Proliferating cells were identified by EdU assay using a Click-iT® Plus EdU Imaging Kit (Invitrogen). Before detection, the cells were incubated with the corresponding medium containing 12.5 μM EdU for 24 h, followed by treatment according to the manufacturer's experimental protocols. A FluoView FV10i Confocal Laser Scanning Microscope was used to acquire images.

ALB Secretion Assay

The secreted ALB concentration was measured using a Rat Albumin ELISA Kit (Bethyl), according to the manufacturer's instructions. Culture supernatants collected from Hep-i (+) cells and Hep-i (–) cells on D9 or D11 were used as test

samples; the Matrigel overlaying the Hep-i (+) cells was collected after being converted into a liquid at 4°C. The mean cell number counted on D9 and D11 was used for normalizing the ALB secretion from D6–D9 and D9–D11, respectively. Cell-free YAC-containing SHM and cell-free Matrigel-mixed hepatic induction media were incubated for the corresponding periods and were regarded as blank controls to establish the baseline. A SpectraMax Paradigm Multi-Mode Detection System (Molecular Devices) was used to measure the absorbance and calculate the results.

Urea Synthesis Assay

A Urea Assay Kit (Abcam) was used to determine the secreted urea concentration. The test samples and blank controls prepared for the ALB secretion assay were also applied to the urea synthesis assay. The urea secretion from D6–D9 and D9–D11 was quantified according to the manufacturer’s instructions and normalized to the mean cell number counted on D9 or D11. A SpectraMax Paradigm Multi-Mode Detection System performed the output measurement and calculation of the results.

FDA Hydrolysis Assay

An FDA assay was used to evaluate the secretory function of Hep-i (+) cells, and to indicate the synthesis capacity of different cells. Cells were incubated with a corresponding medium containing 2.5 μg/mL of FDA (Sigma-Aldrich) in an incubator for 15 min, and then the medium was replaced with HBSS (+) for observation. The hydrolysates of FDA emitted green fluorescence, and their distribution was detected by a FluoView FV10i Confocal Laser Scanning Microscope or a BZ-9000 All-In-One Fluorescence Microscope.

Secretin Assay

The secretory capacity of BEC-i (+) cells was determined by a secretin assay. After washing with PBS 3 times, cells were cultured with HBSS (+) containing 2×10^{-7} M rat secretin (Tocris Bioscience) for 1 h. Image acquisition was performed by a FluoView FV10i Confocal Laser Scanning Microscope.

Proliferation Assay

The proliferation of cells cultured under diverse conditions was measured using a Cell Counting Kit-8 (CCK-8; Dojindo Laboratories). Cells were seeded onto a collagen type I-coated 96-well plate at an initial density of 1×10^4 cells/well, and then cultured under the corresponding conditions and periods with replacement

with fresh medium every 2 days. Cell-free medium cultured during the same period was used as a blank control. For each test, 10 μL/well of CCK-8 solution was added to the medium (including the blank controls) and the cells were cultured for 90 min in an incubator. A GloMax-Multi+ Detection System (Promega) was used to determine absorbance.

Statistical Analysis

All data are presented as the mean±standard deviation (SD). Intergroup differences were identified using one-way analysis of variance, followed by Tukey's multiple comparisons test. Multiple *t*-tests, corrected using the Holm–Sidak method, were used to analyze longitudinal data. Unpaired *t*-tests or Welch's test were applied to determine pairwise differences, depending on whether variance was equal. Differences at P (or adjusted P)<0.05 were considered statistically significant, and Graph Pad Prism 7.0 (GraphPad Software) was used to perform the statistical analysis.

Supplemental References

1. Fu Q, Ohnishi S, Sakamoto N. Conditioned Medium from Human Amnion-Derived Mesenchymal Stem Cells Regulates Activation of Primary Hepatic Stellate Cells. Stem Cells Int. 2018;2018:4898152.
2. Seglen PO. Preparation of isolated rat liver cells. Methods Cell Biol. 1976;13:29-83.
3. Chen Q, Kon J, Ooe H, Sasaki K, Mitaka T. Selective proliferation of rat hepatocyte progenitor cells in serum-free culture. Nat Protoc. 2007;2(5):1197-205.
4. Katsuda T, Kawamata M, Hagiwara K, Takahashi RU, Yamamoto Y, Camargo FD, et al. Conversion of Terminally Committed Hepatocytes to Culturable Bipotent Progenitor Cells with Regenerative Capacity. Cell Stem Cell. 2017;20(1):41-55.
5. Kamiya A, Kojima N, Kinoshita T, Sakai Y, Miyaijma A. Maturation of fetal hepatocytes in vitro by extracellular matrices and oncostatin M: induction of tryptophan oxygenase. Hepatology. 2002;35(6):1351-9.
