# Supplementary material for: Small-molecule inhibitor cocktail promotes the proliferation of pre-existing liver progenitor cells
Source: Stem Cell Reports. 2022 Jun 30;17(7):1589–603. doi: 10.1016/j.stemcr.2022.05.023 (PMC9287679; doi:10.1016/j.stemcr.2022.05.023)
Supplement: Document S2. Article plus supplemental information [file mmc3.pdf]

Stem Cell Reports
Article
OPEN ACCESS

Small-molecule inhibitor cocktail promotes the proliferation of pre-existing liver progenitor cells

Qingjie Fu,[1] Shunsuke Ohnishi,[1,2,*] Goki Suda,[1] and Naoya Sakamoto[1]
[1]Department of Gastroenterology and Hepatology, Hokkaido University Graduate School of Medicine, Sapporo 060-8638, Japan
[2]Laboratory of Molecular and Cellular Medicine, Faculty of Pharmaceutical Sciences, Hokkaido University, Sapporo 060-0812, Japan
*Correspondence: sonishi@pop.med.hokudai.ac.jp
https://doi.org/10.1016/j.stemcr.2022.05.023

SUMMARY

A recent study showed that a cocktail of three small molecules, Y-27632, A83-01, and CHIR99021 (YAC), converts mature hepatocytes (MHs) into proliferative bipotent cells that can be induced into MHs and cholangiocytes in rats. However, when we reproduced these experiments, it was found that bipotent cells may be derived from resident liver progenitor cells (LPCs), whose proliferative activity was promoted by YAC. A simple and efficient sorting scheme was also developed in this study to harvest high-purity and high-yield LPCs. The inducible bipotency of purified LPCs was verified; in addition, they were found to spontaneously differentiate into hepatocytes and cholangiocytes due to changes in proliferative status even without induction. Moreover, during the differentiation process, some hepatocytes spontaneously reconverted to LPCs under certain conditions, such as the release of contact inhibition. These findings may improve our understanding of LPCs and provide a cell source for regenerative medicine.

INTRODUCTION

It is well known that the liver has a powerful regenerative capacity. Mature hepatocytes (MHs) and liver progenitor cells (LPCs), a type of proliferative cells that can differentiate into hepatocytes and cholangiocytes, are believed to be involved in liver regeneration (Espanol-Suner et al., 2012; Fausto and Campbell, 2003; Miyaoka et al., 2012). When the liver is injured and MH proliferation is inhibited, LPCs play a crucial role as building blocks for liver reconstruction (Fausto, 2004). The most cited theory is that LPCs, as a component of ductular reactions, originate in the canals of Hering (Theise et al., 1999); however, their origin is debatable and open to interpretation. MHs are also reported to convert into LPCs and reconstruct the liver (Tarlow et al., 2014). Notably, a combination of three small molecules, i.e., Y-27632 (Rho-associated kinase inhibitor), A83-01 (type 1 transforming growth factor β receptor inhibitor), and CHIR99021 (glycogen synthase kinase-3 inhibitor) (YAC), has been reported to revert rodent MHs to proliferative LPCs termed chemically induced liver progenitors (CLiPs) (Katsuda et al., 2017).

A sufficient understanding of LPCs will not only help comprehend how the liver functions, but will also be beneficial for therapeutic purposes. For patients with end-stage liver disease, liver transplantation is the only curative therapy (Dhawan et al., 2010); however, the shortage of donated organs limits this approach. Although MH transplantation has been recognized as an alternative treatment (Dhawan et al., 2010), great difficulties in expanding MHs *in vitro* restrict their clinical application (Bhatia et al., 2014; Guguen-Guillouzo and Guillouzo, 2010). Thus, transplanting LPCs seems to be a more reasonable option. The methods available currently for the purification of LPCs are mostly based on fluorescence-activated cell sorting (FACS) via cell labeling with specific antibodies (Liu et al., 2019b; Suzuki et al., 2008); however, for clinical application, the safety of antibody-conjugated cell transplantation is also of concern. Therefore, the generation of abundant and clinically available LPCs is a new challenge, and more feasible methods should be developed to attain this goal.

Here, we revisit how YAC works on LPCs and provide a simple and efficient strategy to obtain purified LPCs that may serve as a practical tool for studying liver regeneration and LPC transplantation. Also, we describe how LPC regulates differentiation in response to proliferation signals.

RESULTS

Small-molecule inhibitor cocktail promotes the proliferation of resident LPCs

The combination of Y-27632, A83-01, and CHIR99021, referred to as YAC, has been suggested to convert MHs into culturable bipotent progenitor cells in a previous study (Katsuda et al., 2017). However, we found another possibility for the appearance of culturable cells when culturing rat MHs with YAC. As described in that study, a small hepatocyte culture medium (SHM) was used as the basal medium to culture the freshly isolated MHs (YAC (–) cells). Cells proliferated rapidly in the presence of YAC (YAC (+) cells), reaching a number of cells that was 2.51 ± 0.09 times greater than that of YAC (–) cells after 14 days of culture (Figures 1A and S1A). During cell culture, two types of cells that were morphologically distinct were observed. One of

This is an open access article under the CC BY license (http://creativecommons.org/licenses/by/4.0/).

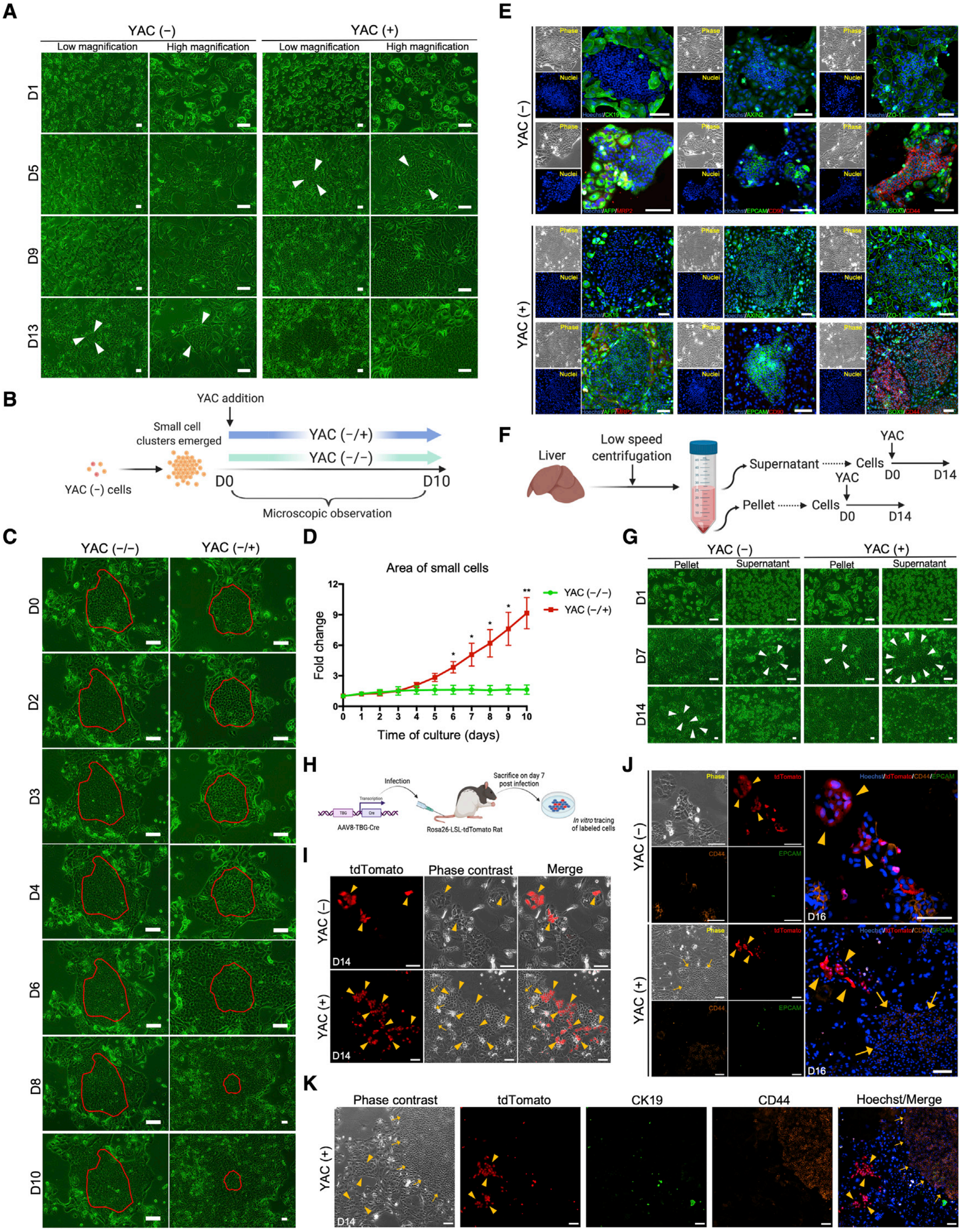

Figure 1. YAC enhances the proliferation of resident rat liver progenitor cells (LPCs; i.e., small cells)

(A) Phase-contrast images of freshly isolated rat MHs cultured with or without YAC. MH, mature hepatocyte; YAC, the combination of Y-27632, A83-01, and CHIR99021; YAC (−), MH culture without YAC; YAC (+), MH culture with YAC.

(B) Schematic representation of the supplementation of YAC (−) cells with YAC. According to the addition of YAC to YAC (−) cells, the culture conditions were termed YAC (−/−) and YAC (−/+).

(C) Representative phase-contrast images of cells with or without additional YAC. The red closed loops denote the initial areas of small cells on day 0.

(legend continued on next page)

them was a small ovoid cell type that proliferated within a short period (Figure 1A). Although small-cell clusters were rare, they were observed in the subsequent culture of YAC (−) cells (Figure 1A). Small cells first emerged on day 7.00 ± 0.39 and day 11.38 ± 0.63 under YAC and YAC-free conditions, respectively (Figure S1B), which indicates that small cells proliferated faster under the effect of YAC, and a long-term culture demonstrated this difference significantly (Figure S1C). To verify whether YAC could promote small-cell proliferation selectively, we further cultured the non-YAC-induced small cells with/without YAC (Figure 1B). In the absence of YAC (YAC (−/−) cells), small cells sustained slow proliferation, whereas under YAC treatment (YAC (−/+) cells), small cells proliferated rapidly without accompanying large-cell growth (Figure 1C). During the 10-day culture, the area of small cells increased by 9.16 ± 0.77-fold under YAC stimulation, far exceeding the 1.64 ± 0.27-fold increase obtained after culture on SHM alone (Figure 1D). Interestingly, YAC did not play a role at the very beginning of the cell culture; rather, it began promoting small-cell proliferation between days 3 and 4 of culture (Figures 1C and 1D), in accordance with the results of a previous study (Katsuda et al., 2017).

Gene expression was analyzed by qRT-PCR in YAC-treated cells on day 14 and it revealed that the LPC marker expressions were upregulated compared with that of fresh MHs (Figure S1D), implying that LPCs were generated under YAC stimulation. To identify LPCs in the cell culture, we performed immunostaining using MH- and LPC-specific markers. The LPC markers EPCAM, CD44, and CD90 (occasionally) were expressed in small cells exclusively (Figures 1E and S1E). Conversely, the expression of another LPC marker CK19 (also a cholangiocyte marker) and the MH marker MRP2 was only observed in surrounding large cells (Figures 1E and S1E). Other widely used LPC markers, such as AFP, AXIN2, and SOX9, were expressed in both cell types (Figure 1E). Based on these results, although the expression pattern of the LPC-specific markers was not entirely consistent with the usual LPC profile, we considered that the small cells were LPCs and the surrounding large cells were mature cells. Furthermore, ZO-1 expression showed tight junctions between cells and revealed that small cells had a high nucleus-to-cytoplasm ratio (Figure 1E), which was in line with the description of LPCs provided in a previous article (Kohn-Gaone et al., 2016). In addition, the individual characteristics of YAC (+) cells did not change during the culture with YAC, compared with YAC (−) cells (Figure 1E), which indicated that YAC does not generate a brand-new cell type.

Since the absence of YAC did not affect the small-cell emergence, we hypothesized that small cells originated from pre-existing cells mingled in the MH fraction during liver cell isolation. In that process, MHs were predominant in the pellet after low-speed centrifugation, whereas other smaller cells were enriched in the supernatant (Chen et al., 2007). Thus, to verify our hypothesis, we cultured the cells obtained from the pellet and the supernatant, respectively (Figure 1F). Under YAC (−) conditions, visible small cells in cultured supernatant-derived cells appeared much earlier than those detected in cultured pellet-derived cells; moreover, in the presence of YAC, when small cells emerged in cultured pellet-derived cells, those derived from supernatant had already proliferated in large quantities (Figure 1G). These results met our expectations, namely, a greater number of pre-existing small cells was associated with the earlier appearance of small-cell clusters.

To explore the origin of small cells accurately, we performed genetic lineage tracing using AAV8-TBG-Cre and Rosa26-LSL-tdTomato rat (Figure 1H) (Igarashi et al., 2016). Although the labeling efficiency was low (Figures S1F and S1H), labeled cells were observed to divide on day 3 (Figure S1G) and were confirmed to be able to proliferate regardless of YAC stimulation (Figure 1I). However, the proliferative tdTomato$^+$ cells were obviously different from the typical small cells in morphology (Figures 1I–1K) and they were hardly expressed CD 44 and EPCAM (Figure 1J), which were confirmed expressing in Rosa26-LSL-tdTomato rat-derived small cells (Figure S1K). Moreover, we sorted only tdTomato$^+$ cells to analyze (Figure S1H), but these cells did not proliferate nor express

(D) The area of small cells was measured at the indicated time points. The values are normalized to the initial area recorded on day 0. The data are expressed as the mean ± SD (n = 3 tracing areas), *p < 0.05; **p < 0.01.
(E) Immunofluorescence staining of LPC markers CK19, AXIN2, AFP, EPCAM, CD90, SOX9, and CD44; the MH marker MRP2; and the tight junction marker ZO-1 in YAC (−) and YAC (+) cells.
(F) Schematic representation showing the method used for isolating and culturing supernatant-derived cells and pellet-derived cells.
(G) Phase-contrast images of supernatant-derived cells and pellet-derived cells cultured with or without YAC.
(H) Schematic representation of the lineage tracing experiments of rat MHs.
(I) Phase-contrast and fluorescence images of tdTomato$^+$ MH-derived cells cultured with or without YAC on day 14 (D14).
(J) Immunofluorescence staining of LPC markers CD44 and EPCAM in Rosa26-LSL-tdTomato rat cells cultured with or without YAC.
(K) Immunofluorescence staining of cholangiocyte marker CK19 and LPC marker CD44 in Rosa26-LSL-tdTomato rat cells cultured with YAC. Scale bars, 100 μm. The arrowheads indicate the small cells that first appeared in corresponding culture conditions in (A) and (G). The arrowheads indicate the tdTomato$^+$ cells and the arrows indicate typical small cells in (I), (J), and (K). See also Figure S1.

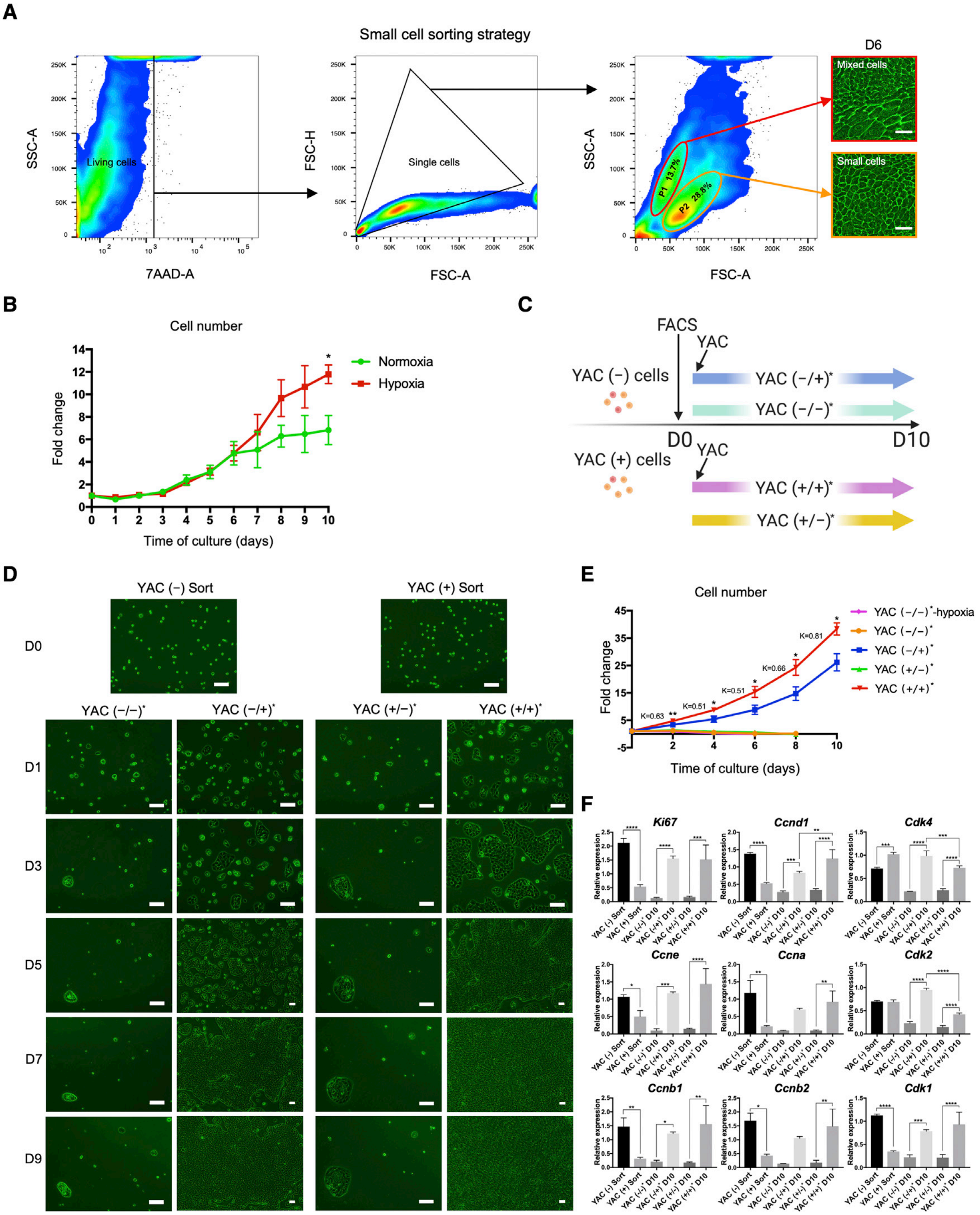

Figure 2. Highly purified small cells can be efficiently sorted by fluorescence-activated cell sorting (FACS)

(A) Strategy used for isolating small cells from YAC (+) cells. Phase-contrast images show the morphology of sorted cells from the P1 and P2 fraction cultured with YAC for 6 days. FSC, forward scatter; SSC, side scatter; 7-AAD, 7-aminoactinomycin D.

(B) The number of YAC (+) Sort cells cultured with YAC under normoxia (20% O_2) and hypoxia (5% O_2). Values are normalized to the initial number of cells recorded on day 0.

(C) Schematic representation of the protocol used for supplementing sorted small cells with YAC. According to whether YAC was applied before and after FACS, the culture conditions were termed YAC (−/−)*, YAC (−/+)*, YAC (+/−)*, and YAC (+/+)*.

(D) Phase-contrast images of sorted small cells cultured in the YAC (−/−)*, YAC (−/+)*, YAC (+/−)*, and YAC (+/+)* conditions.

(legend continued on next page)

LPC markers (Figures S1I and S1J) even culturing with YAC. A previous study also indicated that some cholangiocytes may be mislabeled due to the expression of TBG (Lee et al., 2020), but no CK19 expression was observed in the labeled cells as well (Figure 1K).

Altogether, these results showed that YAC promoted the proliferation of pre-existing LPCs. Considering the apparent difference in morphology between small cells and surrounding cells, we wondered if they could be separated based on this parameter for further analysis.

A large number of highly purified LPCs can be obtained using FACS

We attempted to isolate small cells by FACS from YAC (+) cells that were cultured for 14 days and fully displayed the morphological characteristics. Although it was inconsistent with our expectation that cell clusters with distinct forward scatter would appear due to their different sizes, cells were still clearly divided into two groups (Figure 2A). We sorted these two cell fractions and further cultured with YAC. Cells sorted from P1 still contained two cell types, while cells sorted from P2 (YAC (+) Sort cells) exhibited the small-cell morphology exclusively and generally expressed LPC markers after culturing for 6 days (Figures 2A and S2A). Moreover, the expression of a set of LPC markers in cultured P2 cells was higher than that in cultured P1 cells at both mRNA level and protein level (Figures S2B and S2C). These results demonstrated that we could easily obtain highly purified small cells, i.e., LPCs, by FACS. The diameter of the purified small cells was 16.8 μm on average, which was much smaller than 24.5 μm in freshly isolated MHs (Figure S2D). To investigate whether YAC has effects other than promoting cell proliferation, we sorted small cells from YAC (−) cells (YAC (−) Sort cells) using the same strategy for comparison. Because the proportion of small cells obtained in the absence of YAC was too low, we extended the culture period and sorted cells on day 22 (Figure S2E). Regardless of YAC stimulation, the gene expression of several LPC markers, such as *Afp* and *Sox9*, was almost the same in either sorted cell (on D22); however, that of other genes, such as *Epcam*, *Cd44*, and *Foxj1*, was significantly different in the presence of YAC (Figure S1D). Besides, sorted cells' *Alb* expression was constant at an extremely lower level compared with fresh hepatocytes (Figure S1D). Based on these results, we reconfirmed that YAC does not markedly alter the features of small cells. A prior study demonstrated that the growth of biphenotypic human hepatocytes could be further extended in a hypoxic condition of 5% O_2 (Zhang et al., 2018); thus, we also cultured the sorted small cells using YAC in combination with hypoxia. Notably, hypoxia further enhanced small-cell proliferation from day 7 (Figure 2B), which might provide a more efficient procedure for obtaining abundant cells.

To further elucidate the principle of YAC-induced small-cell proliferation and investigate whether YAC is required to maintain their proliferation, we cultured the sorted small cells in various ways, considering the timing of YAC application (Figure 2C). When YAC (−) Sort cells continued to be cultured without YAC (YAC (−/−)* cells), they could hardly proliferate; furthermore, YAC (+) Sort cells also exhibited a deceleration in the proliferation rate after the withdrawal of YAC (YAC (+/−)* cells) (Figures 2D and 2E), suggesting that YAC is essential for maintaining the continuous proliferation of small cells. Regardless of the presence or absence of YAC in culture before sorting, sorted small cells showed a strong proliferative capacity under YAC treatment; among them, cells that were continuously stimulated by YAC (YAC (+/+)* cells) maintained a rapid growth throughout the experiment, and cells cultured with YAC later (YAC (−/+)* cells) showed a distinct proliferative trend from day 3 (Figures 2D and 2E). The index K was defined as the ratio of the YAC (−/+)* cell proliferation rate to that of the YAC (+/+)* cells. As the cultures continued, the K value gradually increased and reached a value close to 1 (Figure 2E), which indicates that the proliferation rate induced by YAC eventually tended to be consistent, despite the application of YAC stimulation at different time points. Moreover, culturing small cells under hypoxia alone (YAC (−/−)* Hypoxia cells) did not benefit proliferation to a greater extent compared with YAC (−/−)* cells (Figure 2E), implying that although the combination of hypoxia and YAC could further promote small-cell proliferation, hypoxia cannot replace the crucial pro-proliferative role of YAC. Proliferation-related gene expression was assessed by qRT-PCR, which showed that *Ki67* and most cell-cycle-associated gene expression in YAC (−) Sort cells was higher than in YAC (+) Sort cells (Figure 2F), indicating that small

(E) The number of sorted small cells under the corresponding conditions was counted at the indicated time points. Values are normalized to the initial number of cells recorded on D0. The index K is defined as the ratio of the YAC (−/+)* cell proliferation rate to that of YAC (+/+)* cells. The slope between the two time points is regarded as the ratio of cell proliferation. YAC (−/−)*-Hypoxia, YAC (−/−)* cells cultured in the hypoxic condition (5% O_2).
(F) Cell-cycle-related gene expression in freshly sorted small cells and in those cells cultured under YAC (−/−)*, YAC (−/+)*, YAC (+/−)*, and YAC (+/+)* conditions.
The data are shown as the mean ± SD (n = 3 independent experiments), *p < 0.05; **p < 0.01; ***p < 0.001; ****p < 0.0001. Scale bars in (A), 50 μm; in (D), 100 μm. See also Figure S2.

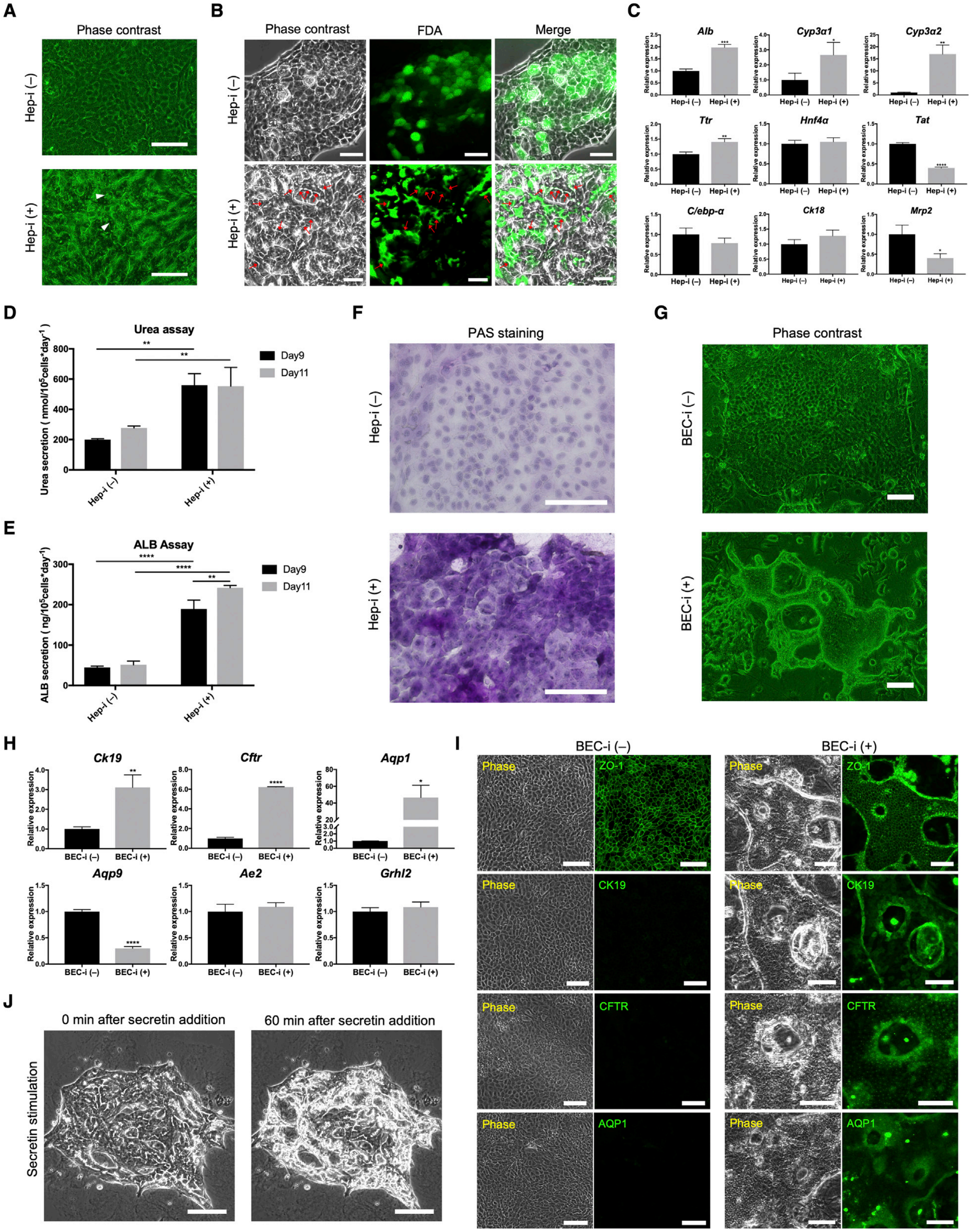

(legend on next page)

cells have an innate potential for self-renewal. In YAC (+) Sort cells, the downregulation of those genes was attributed to the contact inhibition caused by the YAC-induced rapid small-cell proliferation, which was confirmed by analyzing sorted cells at different levels of confluence (Figure S2F). Conversely, the *Cdk4* upregulation and stable *Cdk2* expression (Figure 2F), relating to the G1 and G1/S phases of the cell cycle, respectively (Figure S2G), suggests that YAC (+) Sort cells were still undergoing active substance synthesis and were ready for entering the cell-division stage. Corresponding to the cell proliferation profiles mentioned above, YAC significantly promoted the proliferation-related gene expression in sorted small cells, including *Cdk4* and *Cdk2*; conversely, the expression of those genes was kept lower because of the absence or withdrawal of YAC (Figure 2F). Furthermore, by adding YAC in YAC (−) Sort cells, we confirmed that YAC accelerated small-cell proliferation by shortening the G1 phase (Figure S2H).

We also sorted small cells from YAC-treated pellet- and supernatant-derived cells, with no noticeable difference in small-cell proportion (Figure S2I). The sorted small cells exhibited the same morphology and growth patterns (Figure S2J) with equivalent LPC marker gene expression (Figure S2K), proving that the small cells present in the pellet and supernatant were identical. It is worth mentioning that nonparenchymal cells (NPCs) occasionally appeared among cultured supernatant-derived sorted cells (Figure S2L), probably because they were also small-sized cells and could not be thoroughly eliminated even by FACS.

Using the method described above, we could easily and efficiently obtain a substantial amount of highly purified LPCs. To verify if these LPCs retained their function under YAC stimulation, next we induced the purified small cells to differentiate into MHs and cholangiocytes.

YAC-treated LPCs retain the bipotentiality to differentiate into MHs and cholangiocytes

We used a modified protocol based on a previous study (Kamiya et al., 2002) to induce the hepatocytic differentiation of the YAC (+) Sort cells (Figure S3A). Small cells exposed to hepatic stimulation (Hep-i (+) cells) exhibited a typical MH morphology, such as a polygonal appearance, dual nucleus, and reduced nucleus-to-cytoplasm ratio (Figure 3A). Furthermore, we performed a fluorescein diacetate (FDA) hydrolysis assay and observed fluorescence in the canaliculi-like structures in Hep-i (+) cells, demonstrating induced cell secretory function (Figure 3B). In contrast, the fluorescence in uninduced cells (Hep-i (−) cells) remained inside the cells (Figure 3B). Hep-i (+) cells exhibited a higher expression of several genes related to hepatocytic function, such as *Alb*, *Cyp3a1*, *Cyp3a2*, and *Ttr*, compared with Hep-i (−) cells, whereas other genes, such as *Hnf4α*, *C/ebp-α*, *Ck18*, *Mrp2,* and *Tat*, did not vary significantly or were downregulated (Figure 3C). The high expression of ALB, CK18, HNF4A, and MRP2 showed that Hep-i (+) cells gained MH characteristics; however, Hep-i (−) cells showed almost the same levels as well (Figure S3B). That may be related to the spontaneous hepatic differentiation of Hep-i (−) cells (more hereof later). Moreover, Hep-i (+) cells were more active in urea synthesis and ALB secretion than Hep-i (−) cells, and prolonging the induction period further enhanced these capabilities (Figures 3D and 3E). In addition, this additional improvement in MH physiological function was verified by the upregulation of *Alb* and *Cyp3a2* (Figure S3C), and these gradations showed a continuous process of differentiation into MHs. Glycogen assay, as evaluated by periodic-acid Schiff (PAS) staining, also showed that Hep-i (+) cells synthesized and stored more glycogen (Figure 3F). Along with induction into

Figure 3. Small cells exhibit bipotentiality for being induced into both MHs and cholangiocytes

(A) Phase-contrast images of the sorted small cells with or without hepatic induction. The arrowheads indicate the binucleate cells. Hep-i (−), sorted small cells cultured with YAC alone; Hep-i (+), sorted small cells cultured under hepatic induction.

(B) Uptake and secretion of fluorescein diacetate (FDA) by sorted small cells with or without hepatic induction. The arrows indicate the accumulation of hydrolyzed FDA in the canaliculi-like structures.

(C) MH-related genes expression in Hep-i (−) cells and Hep-i (+) cells.

(D) Urea synthesis after completing conventional (D9) and extended (D11) hepatic induction. Values are normalized to the mean number of cells recorded on D9 or D11, and the corresponding culture period.

(E) Albumin (ALB) secretion after completing normal (D9) and extended (D11) hepatic induction. Values are normalized to the mean number of cells recorded on D9 or D11, and the corresponding culture period.

(F) The glycogen synthesis and storage capacity of Hep-i (+) cells were investigated by periodic-acid Schiff (PAS) staining.

(G) Phase-contrast images of the sorted small cells with or without cholangiocytic induction. BEC-i (−), sorted small cells cultured without cholangiocytic induction; BEC-i (+), sorted small cells cultured under cholangiocytic induction.

(H) Cholangiocyte-related gene expression in BEC-i (−) and BEC-i (+) cells.

(I) Immunofluorescence staining of cholangiocyte markers CK19, CFTR, and AQP1, and the tight junction marker ZO-1 in BEC-i (−) and BEC-i (+) cells.

(J) Phase-contrast images of BEC-i (+) cells before and 60 min after secretin stimulation. See also Video S1.

The data are shown as the mean ± SD (n = 3 independent experiments), $^{*}p < 0.05$; $^{**}p < 0.01$; $^{***}p < 0.001$; $^{****}p < 0.0001$. Scale bars in (B), 50 μm; in (A), (F), (G), (I), and (J), 100 μm. See also Figure S3.

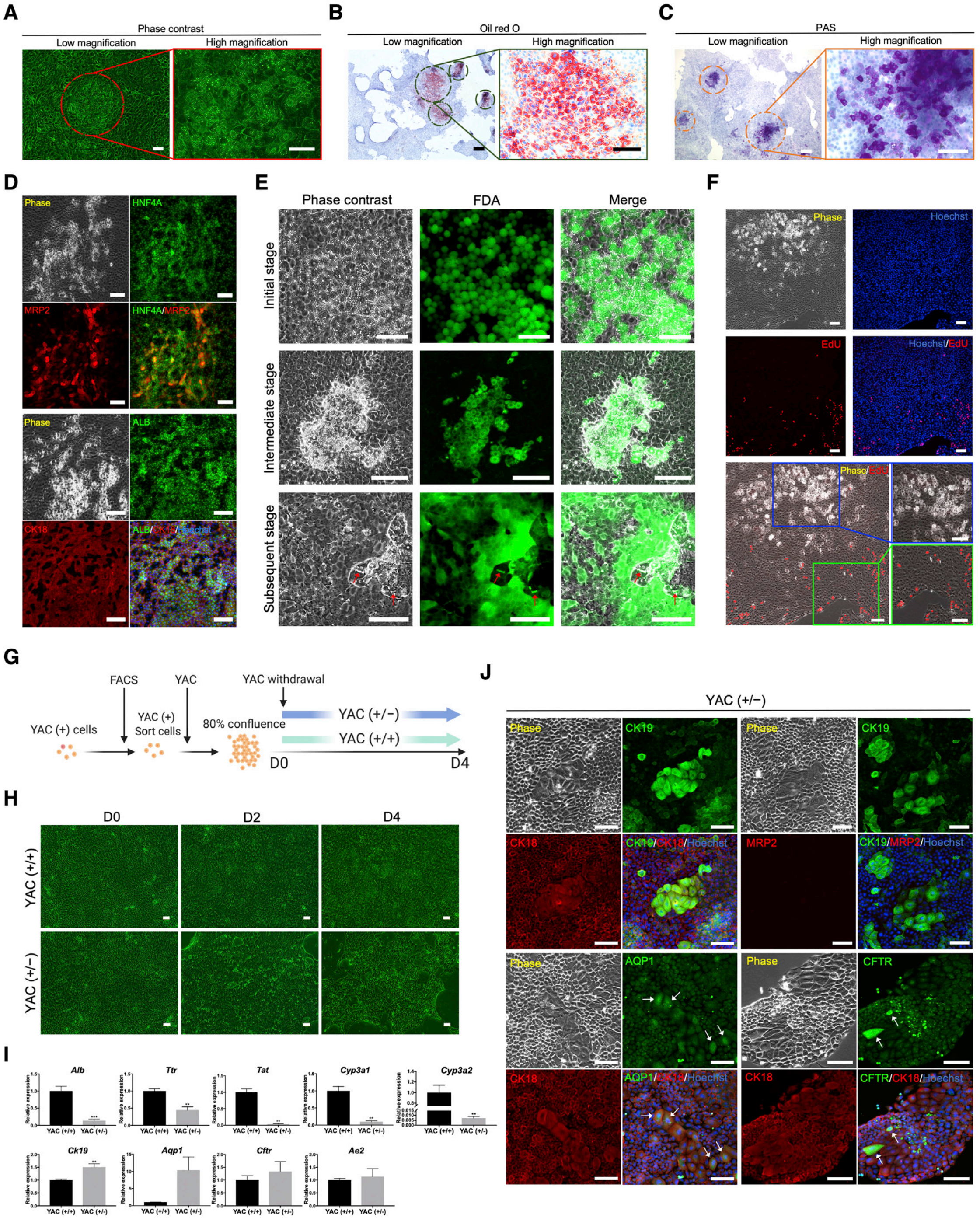

Figure 4. Small cells possess a capacity for spontaneous maturation associated with the cell proliferation status

(A) Emergence of droplet-like components in cultured sorted small cells under YAC stimulation.

(B) The identification of droplet-like components was performed using oil red O staining, indicating their identity as lipid droplets. The dark-green closed loops indicate the positions of cells containing lipid droplets (LDCs) in cell clusters.

(C) The glycogen synthesis and storage capacity of LDCs were investigated by PAS staining. The orange closed loops denote the position of LDCs in cell clusters.

(D) The expression of MH markers HNF4A, MRP2, ALB, and CK18 was confirmed in LDCs by immunofluorescence staining.

(legend continued on next page)

hepatocytes, small cells lost the characteristics of LPCs, as confirmed by the LPC marker gene expression (Figure S3D).

Next, we induced YAC (+) Sort cells into cholangiocytes using a previously reported method (Katsuda et al., 2017; Figure S3E). Small cells that were simply co-cultured with mouse embryonic fibroblasts (BEC-i (−) cells) retained the oval morphology and proliferated as a cell monolayer, while the proliferation of cholangiocytic-induced cells (BEC-i (+) cells) was slowed down with forming a tubular structure (Figure 3G). BEC-i (+) cells expressed a higher level of cholangiocyte marker *Ck19* than BEC-i (−) cells, and *Cftr* and *Aqp1* expression was also increased in BEC-i (+) cells; however, several other cholangiocyte-associated genes, such as *Aqp9*, *Ae2*, and *Crhl2*, were downregulated or remained at the same level (Figure 3H). Consistent with these results, the expression of CK19, CFTR, AQP1, and ZO-1 was confirmed by immunostaining, with particularly high expression observed around the tubular structure (Figure 3I). Notably, ZO-1 expression clearly showed how the tubular structure was formed, similar to that of the intrahepatic bile duct (Rao and Samak, 2013), the lumen was lined with a monolayer of cholangiocyte-like cells with tight junctions sealing the paracellular spaces (Figure S3F). Secretin was used to evaluate the ability to transport water (Nishikawa et al., 2013); the luminal space of BEC-i (+) cells was enlarged under secretin stimulation (Figure 3J and Video S1), showing their secretory properties.

Based on these results, we demonstrated that YAC-treated small cells could still differentiate into MHs and cholangiocytes while gaining significant proliferation capacity.

YAC-treated small cells can differentiate into hepatocytes and cholangiocytes spontaneously

During a long-term culture of sorted small cells with YAC, we incidentally observed that droplet-like components appeared in the cytoplasm of several cells (Figures 4A and S4A), which were recognized as lipid droplets by oil red O staining (Figures 4B and S4B). It is well known that lipid storage is a feature of MHs; therefore, we inferred that those cells containing lipid droplets (LDCs) were MHs. PAS staining confirmed their capacity for glycogen synthesis (Figure 4C), and immunostaining indicated that they expressed higher levels of HNF4A, MRP2, ALB, and CK18 than normal small cells (Figure 4D), demonstrating that LDCs were indeed MHs. These results suggest that, even without induction, small cells can spontaneously differentiate into hepatocytes. Subsequently, we verified that this process was generally divided into three stages: in the initial stage, cells maintained a small-cell morphology with emergence of cytosolic lipid droplets; in the intermediate stage, cells presented the polygonal features of MHs without significant variation in cell size, which was accompanied by a slight decrease in lipid droplets; in the subsequent stage, cells became larger, exhibited visible MH morphological features, and showed secretory functions (Figures 4E and S4A). Moreover, an FDA assay demonstrated that cells involved in a maturation process possessed higher esterase activity (Figure 4E), revealing the enhanced protein-synthesis capacity of MHs. After culturing small cells further, we found that additional LDCs emerged (Figure S4C), suggesting that automatic entry into hepatocytic maturation is a common event in appropriate conditions. Interestingly, LDCs always first emerged in the center of cell clusters (Figures 4A–4C and S4B), where cells may not proliferate because of contact inhibition. Thus, we supposed that this spontaneous conversion was related to their proliferation situation. As expected, maturating cells stopped proliferating, or it should be explained that cells that had stopped proliferating initiated a process of spontaneous hepatocytic differentiation; in contrast, peripheral cells in clusters retained a robust proliferative capacity, as confirmed by EdU assay (Figure 4F).

We demonstrated that YAC played an essential role in maintaining small-cell proliferation, as well as in their differentiation after cells stopped proliferating. Subsequently, we wondered if small cells would enter the spontaneous

(E) Uptake and secretion of FDA by spontaneously matured small cells at different stages. The arrows indicate the accumulation of hydrolyzed FDA in canaliculi-like structures in totally maturated small cells (subsequent stage).
(F) The proliferation status of LDCs was verified by EdU assay. A magnified image of LDCs is shown in the blue box, and a magnified image of peripheral cells is shown in the green box.
(G) Schematic representation of the method used for culturing proliferated small cells with YAC withdrawal. YAC (+/−), YAC withdrawal from massively proliferated sorted small cells; YAC (+/+), YAC retention in sorted small cells cultured with YAC.
(H) Phase-contrast images of proliferated small cells cultured with or without YAC withdrawal.
(I) MH- and cholangiocyte-associated gene expression in small cells cultured with or without YAC withdrawal. The data are expressed as the mean ± SD (n = 3 independent experiments), $^{**}p < 0.01$, $^{***}p < 0.001$.
(J) Immunofluorescence staining of cholangiocyte markers CK18, CK19, AQP1, and CFTR, and the MH marker MRP2 in small cells cultured with YAC withdrawal. The arrows indicate cells expressing AQP1 or CFTR.
Scale bars in (A) high magnification, 50 μm; in (A) low magnification, (B) high magnification, (C) high magnification, (D), (E), (F), (H), and (J), 100 μm; in (B) low magnification, (C) low magnification, 300 μm. See also Figure S4.

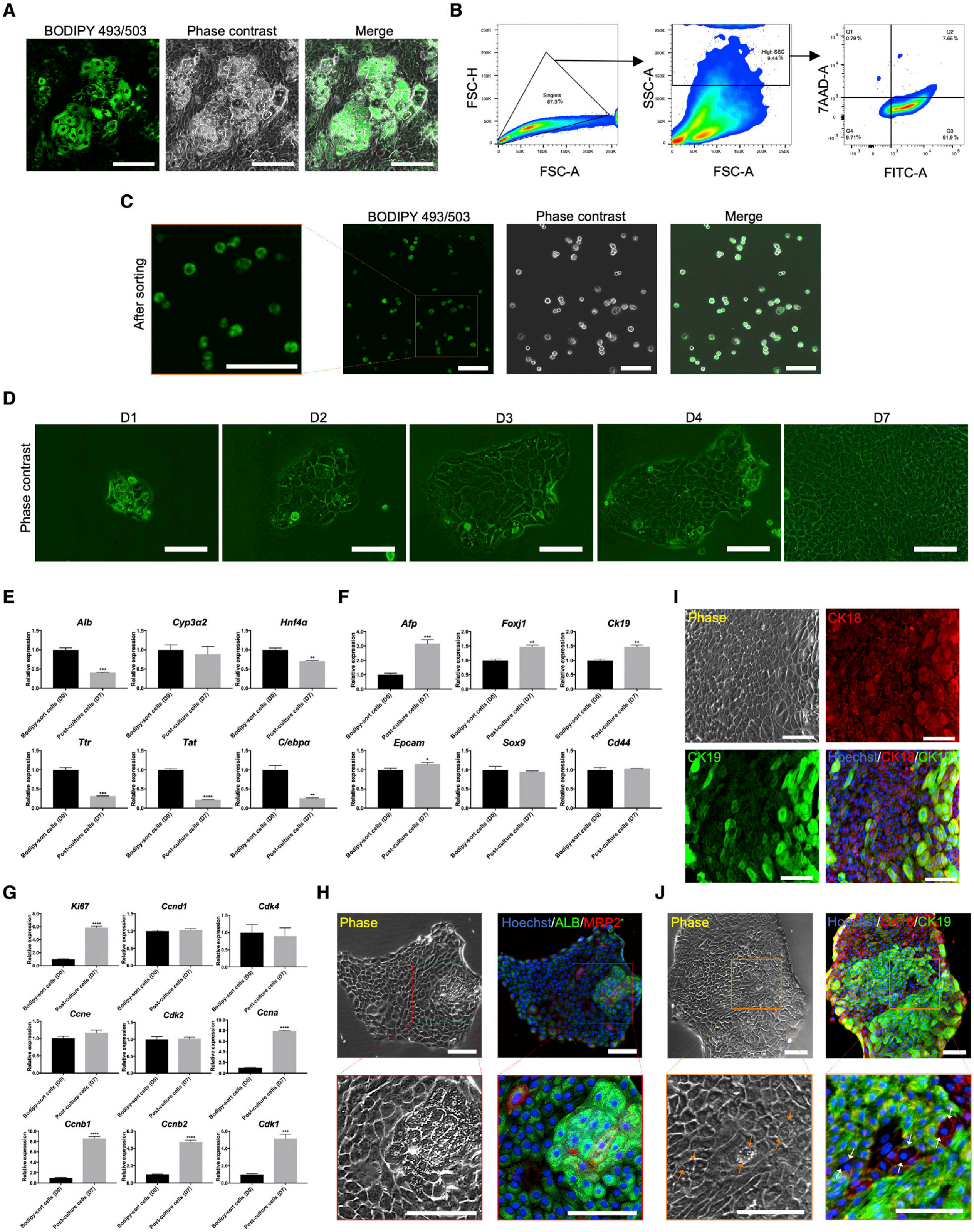

(legend on next page)

hepatocytic maturation process if YAC was withdrawn when cells reached confluence. We discontinued YAC when small cells approached 80% confluence (Figure 4G). Unexpectedly, masses of cells died after YAC withdrawal (YAC (+/−) cells), and MH-shaped cells were barely observed (Figure 4H), suggesting that the persistence of proliferation-stimulation signals was also necessary for cell survival, and the loss of contact inhibition or/and the lack of YAC suspended the maturation of small cells. Furthermore, several cells with larger size and lower nucleus-to-cytoplasm ratio emerged (Figure S4D), indicating that some phenotypic conversion occurred in small cells. qRT-PCR demonstrated that small cells began to differentiate into cholangiocytes rather than hepatocytes after YAC withdrawal (Figure 4I), and enhanced CK19 expression was confirmed in emerging large cells by immunostaining (Figure 4J). Moreover, expression of CK18 but not MRP2 supported their transition into cholangiocytes, and the detection of AQP1 and CFTR proved their secretory function (Figure 4J). Besides, some small cells with decreased nucleus-to-cytoplasm ratio were also observed (Figure S4E). Similar to those large cells, they also expressed cholangiocyte markers rather than MH markers (Figure S4F), which likely demonstrated the continuity of conversion from small cells to cholangiocytes.

Without additional induction, small cells exhibited spontaneous bilineage-differentiation capacity associated with the status of cell proliferation, which provides a useful experimental model to study differentiation mechanisms under conditions resembling physiological status.

Differentiated hepatocytes can re-dedifferentiate into LPCs spontaneously

After passage, LDCs regained their remarkable proliferative activity, accompanied by the disappearance of lipid droplets (Figure S5A). Subsequently, proliferating cells spontaneously re-differentiated into MHs during culture, together with recurrence of lipid droplets and enhanced synthetic activity (Figure S5B). These phenomena suggested the interconvertibility between small cells and LDCs. To explore this conversion relationship more precisely, we attempted to sort LDCs using BODIPY 493/503. Lipid droplets incorporated BODIPY 493/503 (Figure 5A), and FACS was performed when a sufficient number of LDCs appeared. Due to abundant intracellular lipid droplets, a high side scatter area was gated as a candidate, and cells with high fluorescence were sorted subsequently (Figures 5B and S5C). After sorting, the identity of LDCs was re-confirmed by the presence of fluorescence in cell plasma (Figure 5C), and lipid droplets could be easily observed in sorted cells after 1 day in culture (Figure 5D). Consistent with the above results, sorted LDCs resumed significant proliferation accompanied by disappearance of lipid droplets during culture with YAC (Figure 5D). Compared with freshly sorted LDCs, MH-related genes were downregulated and LPC-associated genes were upregulated in cells cultured for 7 days (Figures 5E and 5F). Furthermore, expression of LPC markers CD44 and EPCAM in LDC-derived cells was increased with losing lipid droplets (Figure S5D). These results indicated that LDCs de-differentiated from differentiated hepatocytes into LPCs. Moreover, a cell-cycle-related gene analysis showed that G2/M phase-related genes, such as *Ccna*, *Ccnb1*, *Ccnb2*, and *Cdk1*, were upregulated after culture (Figure 5G), suggesting that LDCs retained proliferative potential but could not divide because of contact inhibition.

To verify whether LDC-derived small cells still reserved the potential for bipotent differentiation, we induced them to differentiate spontaneously using the methods mentioned above. After a long-term culture, LDCs recurred in the small-cell cluster (Figure S5E), accompanied by upregulated expression of MH markers ALB and MRP2 (Figure 5H). Besides, we removed YAC when small cells had proliferated to a considerable number. Some hypertrophic cells emerged subsequently (Figure S5F), which expressed the cholangiocyte markers CK18 and CK19 (Figure 5I).

Figure 5. Spontaneously mature hepatocytes can re-differentiate into fully functional small cells

(A) Lipid droplets were labeled by BODIPY 493/503.

(B) Method used for isolating LDCs (in Q3). FITC, fluorescein isothiocyanate (used to detect BODIPY 493/503).

(C) Verification of sorted LDCs by re-confirming the fluorescence signal.

(D) Phase-contrast images of cultured sorted cells showing that LDCs re-entered proliferation with the disappearance of lipid droplets.

(E) MH-related gene expression in freshly sorted LDCs and those cultured for 7 days.

(F) LPC-specific gene expression in freshly sorted LDCs and those cultured for 7 days.

(G) Cell-cycle-associated gene expression in freshly sorted LDCs and those cultured for 7 days.

(H) Immunofluorescence staining of MH markers ALB and MRP2 in cultured sorted cells.

(I) Immunofluorescence staining of cholangiocyte markers CK18 and CK19 in sorted cells that were induced into spontaneous cholangiocytic maturation.

(J) Immunofluorescence staining of sorted cells that were induced into spontaneous cholangiocytic maturation, showing no CK19 expression in pre-existing LDCs after induction.

The arrows denote LDCs. The data are expressed as the mean ± SD (n = 3 independent experiments), *p < 0.05; **p < 0.01; ***p < 0.001; ****p < 0.0001. Scale bars, 100 μm. See also Figure S5.

Consequently, we believe that LDCs could dedifferentiate into functional LPCs. Interestingly, some emerged LDCs did not change their morphological characteristics significantly after withdrawing YAC (Figure S5G) with expressing CK18, but no CK19 (Figures 5J and S5H), indicating that these LDCs were not affected by YAC withdrawal.

Noticeably, we confirmed that LDCs in the initial stage of hepatocytic differentiation could convert into small cells; however, whether this transformation process is restricted to certain stages is unclear.

DISCUSSION

The findings in our study propose new views on the origin of YAC-induced proliferative LPCs (small cells), and taking advantage of their proliferative properties under YAC stimulation, we purified LPCs and studied their differentiation patterns.

Currently, centrifugation remains the standard method for isolating fresh hepatocytes from rats (Zhang et al., 2016). Despite applying density gradient centrifugation, a non-negligible number of NPCs remain among the purified hepatocytes (Smedsrod and Pertoft, 1985). MHs, the source of CLiPs, were also obtained through centrifugation (Katsuda et al., 2017); therefore, it is probable that LPCs, which are similar in size to NPCs, contaminated the hepatocyte fraction and proliferated under YAC stimulation. Moreover, cells isolated from the supernatant, in which few MHs exist (Chen et al., 2007), were more likely to form small-cell colonies, suggesting that small cells originate from cells other than MHs. SHM is a selective medium for hepatocyte-progenitor cells (Chen et al., 2007), and we prefer that small cells are resident LPCs selected by SHM, which respond to YAC stimulation for further proliferation.

In recent years, small-molecule compounds have been proven to regulate different aspects of cell metabolism. YAC application, in combination or alone, has often been shown to regulate the cell proliferation status, such as maintaining embryonic stem cell self-renewal (Tsutsui et al., 2011), re-activating cardiomyocyte proliferation (Fan et al., 2018), and sustaining hepatoblast multiplication (Lv et al., 2015). Particularly, YAC was reported to be crucial for the long-term culture of hepatocyte-derived proliferative duct-like cells, a type of LPCs (Wu et al., 2017). We believe that YAC plays a similar role in small cells, facilitating the expansion of resident cells by inhibiting ROCK, TGFβR1, and GSK3, which are closely related to cell proliferation. Remarkably, at least two reports mentioned that modified YAC compound also reverses human MHs to proliferative LPCs (Katsuda et al., 2019; Kim et al., 2019); however, we suggest that the precise mechanism underlying the actions of YAC should be evaluated.

MH hypertrophy and hyperplasia are considered the main contributors to liver reconstruction under normal physiological conditions (Miyaoka et al., 2012). However, LPCs that are rarely observed in the normal liver play a crucial role in maintaining hepatocyte homeostasis during chronic liver injury (Espanol-Suner et al., 2012; Tarlow et al., 2014). The origin of LPCs remains highly controversial, and the potential candidate cells include dormant precursor cells (Theise et al., 1999), adult cholangiocytes (Espanol-Suner et al., 2012), hepatocytes (Tarlow et al., 2014), and metaplastic hepatic stellate cells (Kordes et al., 2007, 2014). It is also suggested that the liver has a flexible system of regeneration involving multiple cells, rather than a single type of LPC (Kuwahara et al., 2008). Although various molecular markers, such as AFP, CD133, EPCAM, and CK19, have been identified to describe LPCs, they may express distinct markers based on different origins or growth stages (Miyajima et al., 2014), and this uncertainty brought great difficulties to the identification of primary LPCs. For example, lineage tracing pointed out that $SOX9^+$ cholangiocytes are LPC candidates and can convert into hepatocytes (Furuyama et al., 2011), whereas the latest study on this subject repudiated this conclusion after eliminating the interference of a handful of $SOX9^+$ hepatocytes (He et al., 2017). Given the cell morphology and the profile of marker expression ($CD44^+$/$EPCAM^+$/AFP^+), we believe that the origin of the YAC-induced proliferative small cells is hepatocytic progenitors (Mitaka, 2010); nonetheless, the absence of CK19 expression in small cells was not consistent with the results of the previous report (Mitaka, 2010). This difference in markers may also be explained by the above-mentioned theory that cells are at a variety of differentiation stages (Miyajima et al., 2014). The isolating and purifying method we reported may be an alternative solution for obtaining LPCs, as it avoids deviations caused by marker uncertainty.

Similar to the report that hepatocytic progenitors matured by interacting with NPCs (Mitaka et al., 1999), we found that small cells can mature spontaneously. Cell-cycle proteins are reported playing a role in enforcing pluripotency, and the knockdown of specific cyclins or CDKs results in the loss of the pluripotent state and triggers the differentiation of embryonic stem cells (Liu et al., 2019a). Based on the same logic, the decreased cyclins and CDK expression caused by growth arrest probably facilitates the spontaneous maturation of small cells. After release from contact inhibition, a rebound in the cell-cycle-related protein expression may cause the dedifferentiation of certain cells into proliferative cells during the initial stage of differentiation. To our surprise, despite the observation that YAC withdrawal also created a circumstance of suspended cell proliferation, it resulted in proliferated cells differentiating into cholangiocytes, rather than MHs.

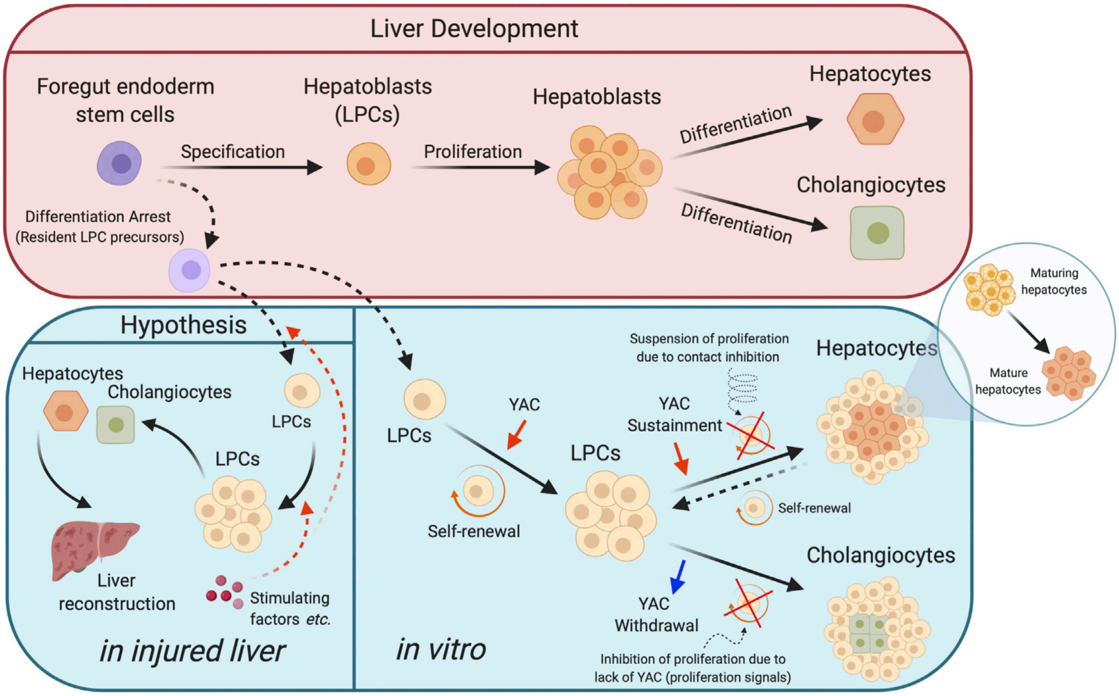

Figure 6. Schematic model of the developmental process of the liver, the transformation characteristics of liver progenitor cells (LPCs) *in vitro,* and the hypothesis of the protective mechanisms of LPCs against liver injury
Although it is not clear why LPCs cannot be directly observed in the normal liver, the current evidence proves their existence, perhaps in the form of their precursors. In *in vitro* culture, by responding to changes in proliferation signals (the presence or absence of YAC, the percentage of cell confluence, etc.), LPCs can regulate and transform spontaneously between proliferation and differentiation statuses. Based on these data, we hypothesize that when liver injury occurs, LPCs are very likely to undergo regulation of their proliferation and differentiation with the same mechanism (the *in vitro* results we reported here) to reconstruct the liver. Created using BioRender.com.

Such evidence indicates that small-cell differentiation is closely related to their proliferation status; however, this association cannot be solely attributed to whether the cells can continue to proliferate. Based on the results of our *in vitro* experiments, we hypothesized that small cells are resident hepatoblast precursors and play the following role in liver regeneration: when liver injury occurs, they first proliferate in large quantities and then gradually mature into functional hepatocytes (Miyajima et al., 2014) (Figure 6).

The capacity of small cells to proliferate abundantly *in vitro*, maintain bipotent differentiation capacity, and mature spontaneously indicates that they are a great tool for studying liver regeneration. The applicability of small cells to humans should be examined, and, if feasible, it is most likely to provide a new approach for the treatment of chronic liver disease, especially cirrhosis.

Limitations of the study

Although SHM is considered as an LPC-selective medium, the possibility that emerging LPCs are cells reprogrammed by SHM cannot be excluded, and SHM contains complex components, whether the pro-proliferation effect of YAC requires the cooperation of some of these substances is unknown.

EXPERIMENTAL PROCEDURES

Animals

Adult male Sprague-Dawley rats (Japan SLC) with a body weight of 400 to 450 g were used for all experiments except lineage tracing (the Rosa26-LSL-tdTomato rat). The Animal Care and Use Committees of Hokkaido University approved the experimental protocol and animal care.

Isolation of MHs and LPCs

Whole liver cells were obtained from rats using a method we reported previously (Fu et al., 2018), and the centrifugation strategy used for isolating MHs and LPCs was in accordance with other procedures (Chen et al., 2007; Seglen, 1976). In brief, after full enzymatic digestion, the cell suspension was first centrifugated for 10 min at 600 × *g*, and then the pellet was resuspended and centrifugated for 1 min at 60 × *g* twice, followed by mixing cells and Percoll, and centrifuging for 10 min at 60 × *g* to isolate MHs (see the

supplemental experimental procedures). For isolating LPCs, the supernatant from the above-mentioned 60 × g centrifugation was collected and centrifuged at 50 × g for 5 min, then the pellet was resuspended and centrifugated for 5 min at 150 × g twice, followed by a final centrifugation at 50 × g for 5 min (see the supplemental experimental procedures).

Cell culture strategy

After isolating MHs and LPCs, we cultured cells in basal medium or under YAC stimulation to evaluate their effects on promoting the proliferation of small cells. When a sufficient amount of small cells appeared in YAC-treated MHs, FACS was performed to purify small cells. To verify the bipotency of small cells, purified cells were induced to hepatocytes and cholangiocytes (Kamiya et al., 2002; Katsuda et al., 2017). Small cells also showed the ability to differentiate spontaneously after proliferation, and we controlled the direction of spontaneous differentiation by adding or withdrawing YAC. The concrete steps of the above-mentioned experimental procedures and related analysis are described in the supplemental experimental procedures, and Tables S1 and S2.

SUPPLEMENTAL INFORMATION

Supplemental information can be found online at https://doi.org/10.1016/j.stemcr.2022.05.023.

AUTHOR CONTRIBUTIONS

Conceptualization, Q.F. and S.O.; Methodology, Q.F., G.S., and S.O.; Validation, Q.F.; Formal analysis, Q.F.; Investigation, Q.F.; Resources, Q.F.; Data Curation, Q.F.; Writing-Original Draft, Q.F.; Writing-Review & Editing, S.O.; Visualization, Q.F.; Supervision, N.S.; Project administration, S.O.; Funding acquisition, N.S.

ACKNOWLEDGMENTS

This study was supported by a Grant-in-Aid (B) from the Japan Society for the Promotion of Science (JSPS, 19H03630). Q.F. received scholarships from Otsuka Toshimi Scholarship Foundation (No. 18-60 and No. 19-43). Rosa26-LSL-tdTomato rats were generated by Hiroyuki Igarashi et al. and provided by NBRP-Rat with support in part by the National BioResource Project of the MEXT, Japan. AAV-TBG-Cre viral preps were produced by Addgene using plasmids gifted by Dr. James M. Wilson. The authors thank Enago (www.enago.jp) for the English language review.

CONFLICT OF INTERESTS

The authors declare no competing interests.

Received: March 3, 2021
Revised: May 31, 2022
Accepted: May 31, 2022
Published: June 30, 2022

REFERENCES

Bhatia, S.N., Underhill, G.H., Zaret, K.S., and Fox, I.J. (2014). Cell and tissue engineering for liver disease. Sci. Transl. Med. *6*, 245sr2. https://doi.org/10.1126/scitranslmed.3005975.

Chen, Q., Kon, J., Ooe, H., Sasaki, K., and Mitaka, T. (2007). Selective proliferation of rat hepatocyte progenitor cells in serum-free culture. Nat. Protoc. *2*, 1197–1205. https://doi.org/10.1038/nprot.2007.118.

Dhawan, A., Puppi, J., Hughes, R.D., and Mitry, R.R. (2010). Human hepatocyte transplantation: current experience and future challenges. Nat. Rev. Gastroenterol. Hepatol. *7*, 288–298. https://doi.org/10.1038/nrgastro.2010.44.

Español–Suñer, R., Carpentier, R., Van Hul, N., Legry, V., Achouri, Y., Cordi, S., Jacquemin, P., Lemaigre, F., and Leclercq, I.A. (2012). Liver progenitor cells yield functional hepatocytes in response to chronic liver injury in mice. Gastroenterology *143*, 1564–1575.e7. https://doi.org/10.1053/j.gastro.2012.08.024.

Fan, Y., Ho, B.X., Pang, J.K.S., Pek, N.M.Q., Hor, J.H., Ng, S.Y., and Soh, B.S. (2018). Wnt/β-catenin-mediated signaling re-activates proliferation of matured cardiomyocytes. Stem Cell Res. Ther. *9*, 338. https://doi.org/10.1186/s13287-018-1086-8.

Fausto, N. (2004). Liver regeneration and repair: hepatocytes, progenitor cells, and stem cells. Hepatology *39*, 1477–1487. https://doi.org/10.1002/hep.20214.

Fausto, N., and Campbell, J.S. (2003). The role of hepatocytes and oval cells in liver regeneration and repopulation. Mech. Dev. *120*, 117–130. https://doi.org/10.1016/s0925-4773(02)00338-6.

Fu, Q., Ohnishi, S., and Sakamoto, N. (2018). Conditioned medium from human amnion-derived mesenchymal stem cells regulates activation of primary hepatic stellate cells. Stem Cell. Int. *2018*, 4898152. https://doi.org/10.1155/2018/4898152.

Furuyama, K., Kawaguchi, Y., Akiyama, H., Horiguchi, M., Kodama, S., Kuhara, T., Hosokawa, S., Elbahrawy, A., Soeda, T., Koizumi, M., et al. (2011). Continuous cell supply from a Sox9-expressing progenitor zone in adult liver, exocrine pancreas and intestine. Nat. Genet. *43*, 34–41. https://doi.org/10.1038/ng.722.

Guguen-Guillouzo, C., and Guillouzo, A. (2010). General review on in vitro hepatocyte models and their applications. Methods Mol. Biol. *640*, 1–40. https://doi.org/10.1007/978-1-60761-688-7_1.

He, L., Li, Y., Li, Y., Pu, W., Huang, X., Tian, X., Wang, Y., Zhang, H., Liu, Q., Zhang, L., et al. (2017). Enhancing the precision of genetic lineage tracing using dual recombinases. Nat. Med. *23*, 1488–1498. https://doi.org/10.1038/nm.4437.

Igarashi, H., Koizumi, K., Kaneko, R., Ikeda, K., Egawa, R., Yanagawa, Y., Muramatsu, S.i., Onimaru, H., Ishizuka, T., and Yawo, H. (2016). A novel reporter rat strain that conditionally expresses the bright red fluorescent protein tdTomato. PLoS One *11*, e0155687. https://doi.org/10.1371/journal.pone.0155687.

Kamiya, A., Kojima, N., Kinoshita, T., Sakai, Y., and Miyaijma, A. (2002). Maturation of fetal hepatocytes in vitro by extracellular matrices and oncostatin M: induction of tryptophan oxygenase. Hepatology *35*, 1351–1359. https://doi.org/10.1053/jhep.2002.33331.

Katsuda, T., Kawamata, M., Hagiwara, K., Takahashi, R.U., Yamamoto, Y., Camargo, F.D., and Ochiya, T. (2017). Conversion of terminally committed hepatocytes to culturable bipotent progenitor cells with regenerative capacity. Cell Stem Cell *20*, 41–55. https://doi.org/10.1016/j.stem.2016.10.007.

Katsuda, T., Matsuzaki, J., Yamaguchi, T., Yamada, Y., Prieto-Vila, M., Hosaka, K., Takeuchi, A., Saito, Y., and Ochiya, T. (2019). Generation of human hepatic progenitor cells with regenerative and metabolic capacities from primary hepatocytes. Elife *8*, e47313. https://doi.org/10.7554/elife.47313.

Kim, Y., Kang, K., Lee, S.B., Seo, D., Yoon, S., Kim, S.J., Jang, K., Jung, Y.K., Lee, K.G., Factor, V.M., et al. (2019). Small molecule-mediated reprogramming of human hepatocytes into bipotent progenitor cells. J. Hepatol. *70*, 97–107. https://doi.org/10.1016/j.jhep.2018.09.007.

Köhn-Gaone, J., Gogoi-Tiwari, J., Ramm, G.A., Olynyk, J.K., and Tirnitz-Parker, J.E.E. (2016). The role of liver progenitor cells during liver regeneration, fibrogenesis, and carcinogenesis. Am. J. Physiol. Gastrointest. Liver Physiol. *310*, G143–G154. https://doi.org/10.1152/ajpgi.00215.2015.

Kordes, C., Sawitza, I., Götze, S., Herebian, D., and Häussinger, D. (2014). Hepatic stellate cells contribute to progenitor cells and liver regeneration. J. Clin. Invest. *124*, 5503–5515. https://doi.org/10.1172/jci74119.

Kordes, C., Sawitza, I., Müller-Marbach, A., Ale-Agha, N., Keitel, V., Klonowski-Stumpe, H., and Häussinger, D. (2007). CD133+ hepatic stellate cells are progenitor cells. Biochem. Biophys. Res. Commun. *352*, 410–417. https://doi.org/10.1016/j.bbrc.2006.11.029.

Kuwahara, R., Kofman, A.V., Landis, C.S., Swenson, E.S., Barendswaard, E., and Theise, N.D. (2008). The hepatic stem cell niche: identification by label-retaining cell assay. Hepatology *47*, 1994–2002. https://doi.org/10.1002/hep.22218.

Lee, S., Zhou, P., Whyte, S., and Shin, S. (2020). Adeno-associated virus serotype 8-mediated genetic labeling of cholangiocytes in the neonatal murine liver. Pharmaceutics *12*, 351. https://doi.org/10.3390/pharmaceutics12040351.

Liu, L., Michowski, W., Kolodziejczyk, A., and Sicinski, P. (2019a). The cell cycle in stem cell proliferation, pluripotency and differentiation. Nat. Cell Biol. *21*, 1060–1067. https://doi.org/10.1038/s41556-019-0384-4.

Liu, W., Wang, Y., Sun, Y., Wu, Y., Ma, Q., Shi, Y., He, R., Zhang, T., Ma, Y., Zuo, W., et al. (2019b). Clonal expansion of hepatic progenitor cells and differentiation into hepatocyte-like cells. Dev. Growth Differ. *61*, 203–211. https://doi.org/10.1111/dgd.12596.

Lv, L., Han, Q., Chu, Y., Zhang, M., Sun, L., Wei, W., Jin, C., and Li, W. (2015). Self-renewal of hepatoblasts under chemically defined conditions by iterative growth factor and chemical screening. Hepatology *61*, 337–347. https://doi.org/10.1002/hep.27421.

Mitaka, T. (2010). Studies of Liver Cells: what are "small hepatocytes?". Tumor Res. *45*, 45–58.

Mitaka, T., Sato, F., Mizuguchi, T., Yokono, T., and Mochizuki, Y. (1999). Reconstruction of hepatic organoid by rat small hepatocytes and hepatic nonparenchymal cells. Hepatology *29*, 111–125. https://doi.org/10.1002/hep.510290103.

Miyajima, A., Tanaka, M., and Itoh, T. (2014). Stem/progenitor cells in liver development, homeostasis, regeneration, and reprogramming. Cell Stem Cell *14*, 561–574. https://doi.org/10.1016/j.stem.2014.04.010.

Miyaoka, Y., Ebato, K., Kato, H., Arakawa, S., Shimizu, S., and Miyajima, A. (2012). Hypertrophy and unconventional cell division of hepatocytes underlie liver regeneration. Curr. Biol. *22*, 1166–1175. https://doi.org/10.1016/j.cub.2012.05.016.

Nishikawa, Y., Sone, M., Nagahama, Y., Kumagai, E., Doi, Y., Omori, Y., Yoshioka, T., Tokairin, T., Yoshida, M., Yamamoto, Y., et al. (2013). Tumor necrosis factor-alpha promotes bile ductular transdifferentiation of mature rat hepatocytes in vitro. J. Cell. Biochem. *114*, 831–843. https://doi.org/10.1002/jcb.24424.

Rao, R.K., and Samak, G. (2013). Bile duct epithelial tight junctions and barrier function. Tissue Barriers *1*, e25718. https://doi.org/10.4161/tisb.25718.

Seglen, P.O. (1976). Preparation of isolated rat liver cells. Methods Cell Biol. *13*, 29–83. https://doi.org/10.1016/s0091-679x(08)61797-5.

Smedsrød, B., and Pertoft, H. (1985). Preparation of pure hepatocytes and reticuloendothelial cells in high yield from a single rat liver by means of Percoll centrifugation and selective adherence. J. Leukoc. Biol. *38*, 213–230. https://doi.org/10.1002/jlb.38.2.213.

Suzuki, A., Sekiya, S., Onishi, M., Oshima, N., Kiyonari, H., Nakauchi, H., and Taniguchi, H. (2008). Flow cytometric isolation and clonal identification of self-renewing bipotent hepatic progenitor cells in adult mouse liver. Hepatology *48*, 1964–1978. https://doi.org/10.1002/hep.22558.

Tarlow, B.D., Pelz, C., Naugler, W.E., Wakefield, L., Wilson, E.M., Finegold, M.J., and Grompe, M. (2014). Bipotential adult liver progenitors are derived from chronically injured mature hepatocytes. Cell Stem Cell *15*, 605–618. https://doi.org/10.1016/j.stem.2014.09.008.

Theise, N.D., Saxena, R., Portmann, B.C., Thung, S.N., Yee, H., Chiriboga, L., Kumar, A., and Crawford, J.M. (1999). The canals of Hering and hepatic stem cells in humans. Hepatology *30*, 1425–1433. https://doi.org/10.1002/hep.510300614.

Tsutsui, H., Valamehr, B., Hindoyan, A., Qiao, R., Ding, X., Guo, S., Witte, O.N., Liu, X., Ho, C.M., and Wu, H. (2011). An optimized small molecule inhibitor cocktail supports long-term maintenance of human embryonic stem cells. Nat. Commun. *2*, 167. https://doi.org/10.1038/ncomms1165.

Wu, H., Zhou, X., Fu, G.B., He, Z.Y., Wu, H.P., You, P., Ashton, C., Wang, X., Wang, H.Y., and Yan, H.X. (2017). Reversible transition between hepatocytes and liver progenitors for in vitro hepatocyte expansion. Cell Res. *27*, 709–712. https://doi.org/10.1038/cr.2017.47.

Zhang, K., Zhang, L., Liu, W., Ma, X., Cen, J., Sun, Z., Wang, C., Feng, S., Zhang, Z., Yue, L., et al. (2018). In vitro expansion of primary human hepatocytes with efficient liver repopulation capacity. Cell Stem Cell *23*, 806–819.e4. https://doi.org/10.1016/j.stem.2018.10.018.

Zhang, Q., Qu, Y., Li, Z., Zhang, Q., Xu, M., Cai, X., Li, F., and Lu, L. (2016). Isolation and culture of single cell types from rat liver. Cells Tissues Organs *201*, 253–267. https://doi.org/10.1159/000444672.

Stem Cell Reports, Volume *17*

Supplemental Information

Small-molecule inhibitor cocktail promotes the proliferation of pre-existing liver progenitor cells

Qingjie Fu, Shunsuke Ohnishi, Goki Suda, and Naoya Sakamoto

Figure S1

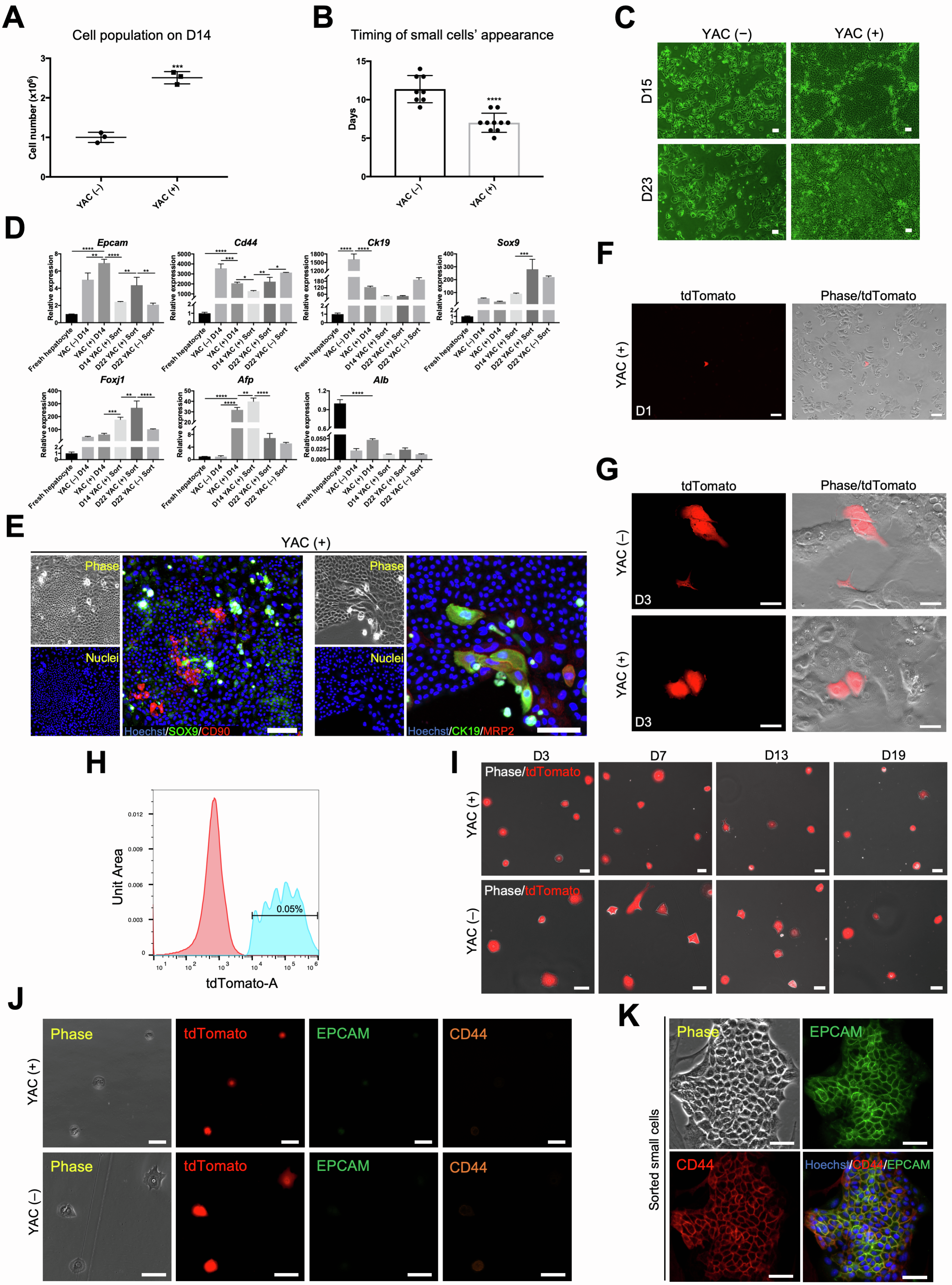

A
Cell population on D14
Cell number (x10⁶)
YAC (–)
YAC (+)
B
Timing of small cells' appearance
Days
C
YAC (−)
YAC (+)
D15
D23
D
Epcam
Cd44
Ck19
Sox9
Foxj1
Afp
Alb
Relative expression
Fresh hepatocyte
YAC (–) D14
YAC (+) D14
D14 YAC (+) Sort
D22 YAC (+) Sort
D22 YAC (–) Sort
E
YAC (+)
Phase
Nuclei
Hoechst/SOX9/CD90
Hoechst/CK19/MRP2
F
tdTomato
Phase/tdTomato
D1
G
D3
H
Unit Area
tdTomato-A
0.05%
I
D3
D7
D13
D19
Phase/tdTomato
J
Phase
tdTomato
EPCAM
CD44
K
Sorted small cells
Hoechst/CD44/EPCAM

Figure S1. Characterization of YAC-treated cells (related to Figure 1)

(A) The number of cells on D14 of culture. The data are expressed as the mean ± SD (n = 3 independent experiments), $^{***}P < 0.001$. YAC (–), cells cultured without YAC; YAC (+), cells cultured under YAC stimulation. (B) Time points of the first appearance of small cells. The data are expressed as the mean ± SD (YAC (–), n=8 independent experiments, YAC (+), n=10 independent experiments), $^{****}P < 0.0001$. (C) Phase-contrast images of cells treated with or without YAC in long-term culture. Scale bars, 100 μm. (D) Gene expression of LPC markers *Epcam*, *Cd44*, *CK19*, *Sox9*, *Foxj1*, and *Afp*, and of the MH marker *Alb* in fresh hepatocytes, YAC (–) cells, YAC (+) cells, YAC (–) Sort cells, and YAC (+) Sort cells. The data are expressed as the mean ± SD (n = 3 independent experiments), $^{*}P < 0.05$, $^{**}P < 0.01$, $^{***}P < 0.001$, $^{****}P < 0.0001$. LPC, liver progenitor cell; MH, mature hepatocyte; YAC (–) Sort cells, small cells sorted from YAC (–) cells; YAC (+) Sort cells, small cells sorted from YAC (+) cells. (E) Expression of LPC markers CD90 and SOX9 in small cells, and of the cholangiocyte marker (also an LPC marker) CK19 and the MH marker MRP2 in large cells. Scale bars, 100 μm. (F) Phase-contrast and fluorescence images of tdTomato^{+} MHs on D1. Scale bars, 100 μm.

(G) Phase-contrast and fluorescence images of tdTomato$^+$ MH-derived proliferative cells cultured with or without YAC on D3. Scale bars, 50 μm. (H) Gating strategy for tdTomato$^+$ MHs and ratio of tdTomato$^+$ MHs when performing FACS. (I) Images of sorted tdTomato$^+$ MHs cultured with or without YAC over time. Scale bars, 100 μm. (J) Expression of LPC markers CD44 and EPCAM in sorted tdTomato$^+$ MHs cultured with or without YAC on D21. Scale bars, 100 μm. (K) Expression of LPC markers CD44 and EPCAM in small cells sorted from Rosa26-LSL-tdTomato rat MHs cultured with YAC. Scale bars, 100 μm. The detailed sorting strategy is introduced in Figure 2A.

Figure S2

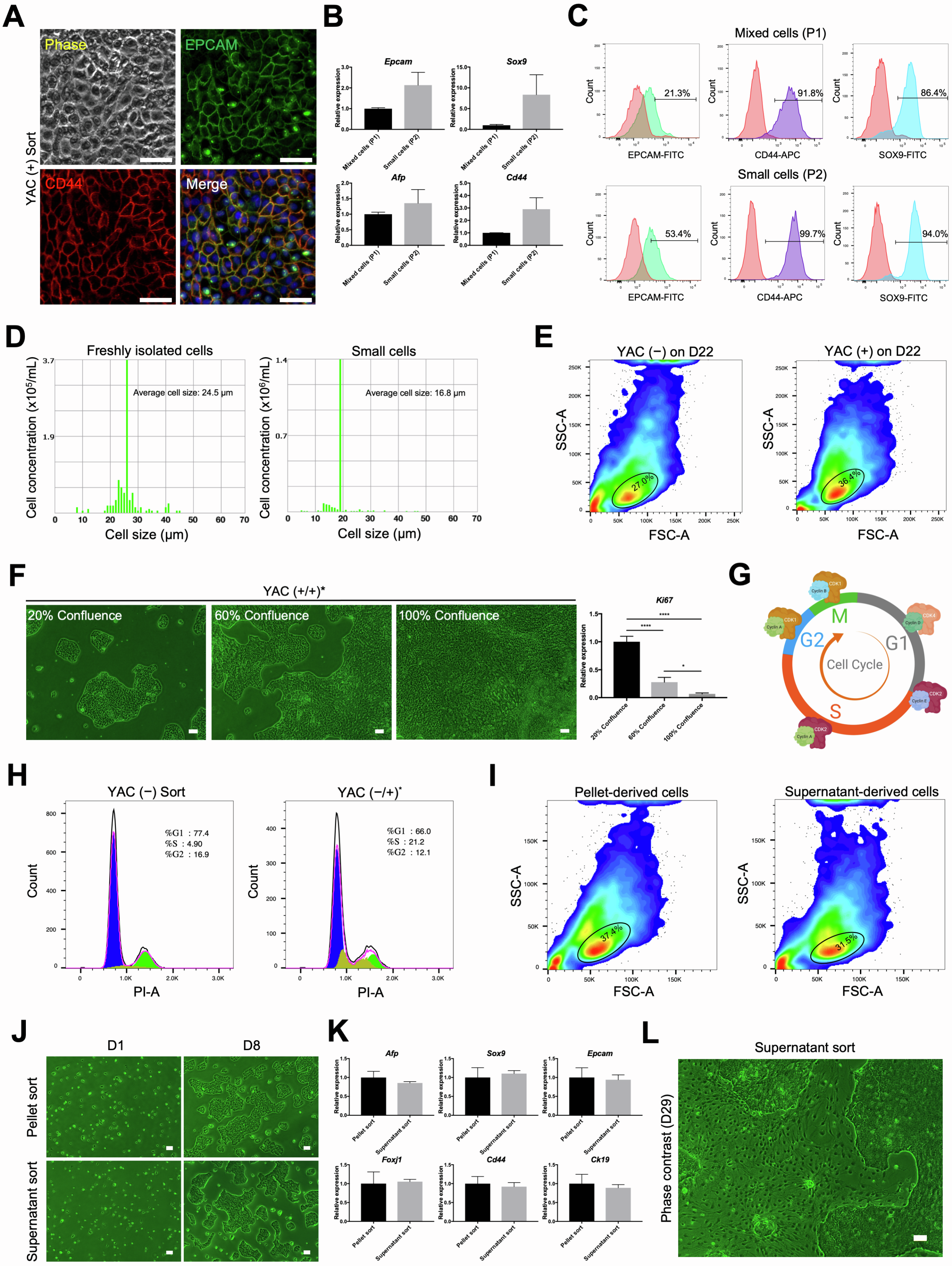

A
Phase
EPCAM
CD44
Merge
YAC (+) Sort
B
Epcam
Sox9
Afp
Cd44
Relative expression
Mixed cells (P1)
Small cells (P2)
C
Mixed cells (P1)
Small cells (P2)
Count
21.3%
91.8%
86.4%
53.4%
99.7%
94.0%
EPCAM-FITC
CD44-APC
SOX9-FITC
D
Freshly isolated cells
Small cells
Average cell size: 24.5 μm
Average cell size: 16.8 μm
Cell concentration (x10^5/mL)
Cell concentration (x10^6/mL)
Cell size (μm)
E
YAC (−) on D22
YAC (+) on D22
27.0%
36.4%
SSC-A
FSC-A
F
YAC (+/+)*
20% Confluence
60% Confluence
100% Confluence
Ki67
Relative expression
G
M
G1
S
G2
Cell Cycle
CDK1
Cyclin B
Cyclin A
CDK4
Cyclin D
CDK2
Cyclin E
H
YAC (−) Sort
YAC (−/+)*
%G1 : 77.4
%S : 4.90
%G2 : 16.9
%G1 : 66.0
%S : 21.2
%G2 : 12.1
Count
PI-A
I
Pellet-derived cells
Supernatant-derived cells
37.4%
31.5%
SSC-A
FSC-A
J
D1
D8
Pellet sort
Supernatant sort
K
Afp
Sox9
Epcam
Foxj1
Cd44
Ck19
Relative expression
Pellet sort
Supernatant sort
L
Supernatant sort
Phase contrast (D29)

Figure S2. Characterization of YAC-induced proliferative small cells (related to Figure 2)

(A) Expression of LPC markers EPCAM and CD44 in YAC (+) Sort cells. Scale bars, 100 µm. (B) Expression of the LPC-related genes *Epcam*, *Sox9*, *Afp*, and *Cd44* in sorted small cells (P2 cells) and in a cell mixture (P1 cells). The data are expressed as the mean ± SD (n = 3 independent experiments). See also Figure 2A. (C) Expression of LPC markers EPCAM, CD44 and SOX9 in sorted P1 cells and P2 cells. (D) Size of fresh MHs and sorted small cells. Cell counting and the measurement of cell size were performed using a Luna Automated Cell Counter. (E) Phase-contrast images of cultured YAC (+/+)* cells at different confluence levels and the corresponding gene expression of *Ki67.* The data are expressed as the mean ± SD (n = 3), $^{*}P < 0.05$, $^{****}P < 0.0001$. YAC (+/+)* cells, YAC (+) Sort cells cultured with YAC. (F) Phase-contrast images of cultured YAC (+/+)* cells at different confluence levels and the corresponding gene expression of *Ki67.* The data are expressed as the mean ± SD (n = 3 independent experiments), $^{*}P < 0.05$, $^{****}P < 0.0001$. YAC (+/+)* cells, YAC (+) Sort cells cultured with YAC. (G) Schematic representation of the cell cycle, showing the corresponding cyclins and cyclin-dependent kinases (CDKs) at each stage. (H) Cell cycle analysis of YAC (–)

Sort cells and YAC (–/+)* cells. YAC (–/+)* cells were treated with YAC until 30% confluence. PI, propidium iodide; YAC (–/+)* cells, YAC (–) Sort cells cultured with YAC. (I) Ratio of small cells among pellet-derived cells and supernatant-derived cells cultured with YAC for 14 days. (J) Phase-contrast images showing the proliferation of small cells sorted from YAC-treated pellet-derived cells and supernatant-derived cells under YAC stimulation. Scale bars, 100 μm. (K) Expression of LPC-related genes *Afp, Sox9, Epcam, Foxj1, Cd44* and *Ck19* in pellet-derived small cells and supernatant-derived small cells. The data are expressed as the mean ± SD (*n* = 3 independent experiments). (L) The phase-contrast image of cultured small cells sorted from YAC-treated supernatant-derived cells on D29, showing the appearance of other types of cells. Scale bar, 100 μm.

Figure S3

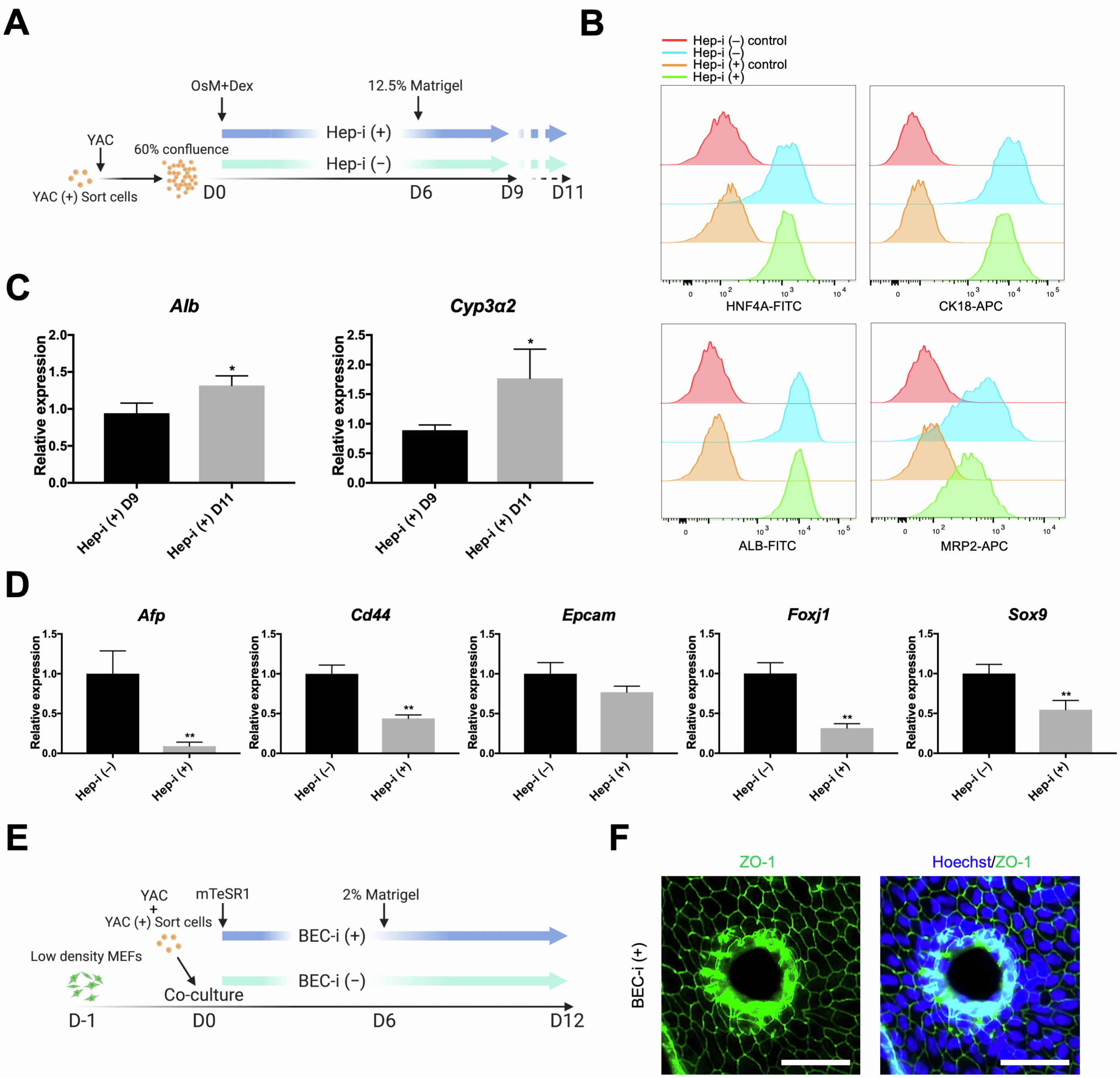
A
OsM+Dex
12.5% Matrigel
Hep-i (+)
Hep-i (–)
YAC
60% confluence
YAC (+) Sort cells
D0
D6
D9
D11
B
Hep-i (–) control
Hep-i (–)
Hep-i (+) control
Hep-i (+)
HNF4A-FITC
CK18-APC
ALB-FITC
MRP2-APC
C
Alb
Cyp3α2
Relative expression
Hep-i (+) D9
Hep-i (+) D11
D
Afp
Cd44
Epcam
Foxj1
Sox9
Hep-i (–)
Hep-i (+)
E
YAC
+
YAC (+) Sort cells
mTeSR1
2% Matrigel
BEC-i (+)
BEC-i (–)
Low density MEFs
Co-culture
D-1
D0
D6
D12
F
ZO-1
Hoechst/ZO-1
BEC-i (+)

Figure S3. Characterization of induced hepatocytes and cholangiocytes derived from small cells (related to Figure 3)

(A) Schematic representation of hepatocytic induction. Hep-i (–), YAC (+) Sort cells cultured with YAC alone; Hep-i (+), YAC (+) Sort cells cultured under hepatic induction. (B) Expression of MH markers ALB, CK18, HNF4A, and MRP2 in Hep-i (–) cells and Hep-i (+) cells, as determined by flow cytometry. (C) Gene expression of the MH functional markers *Alb* and *Cyp3α2* in Hep-i (+) cells on D9 and D11. The data are expressed as the mean ± SD (n = 3 independent experiments), $^{*}P < 0.05$. (D) Gene expression of LPC markers *Afp*, *Cd44*, *Epcam*, *Foxj1*, and *Sox9* in Hep-i (–) and Hep-i (+) cells on D9. The data are expressed as the mean ± SD (n = 3 independent experiments), $^{**}P < 0.01$. (E) Schematic representation of cholangiocytic induction. MEF, mouse embryonic fibroblast; BEC-i (–), YAC (+) Sort cells cultured without cholangiocytic induction; BEC-i (+), YAC (+) Sort cells cultured under cholangiocytic induction. (F) Expression of the tight junction marker ZO-1 in BEC-i (+) cells, showing a typical bile duct structure. Scale bars, 50 μm.

Figure S4

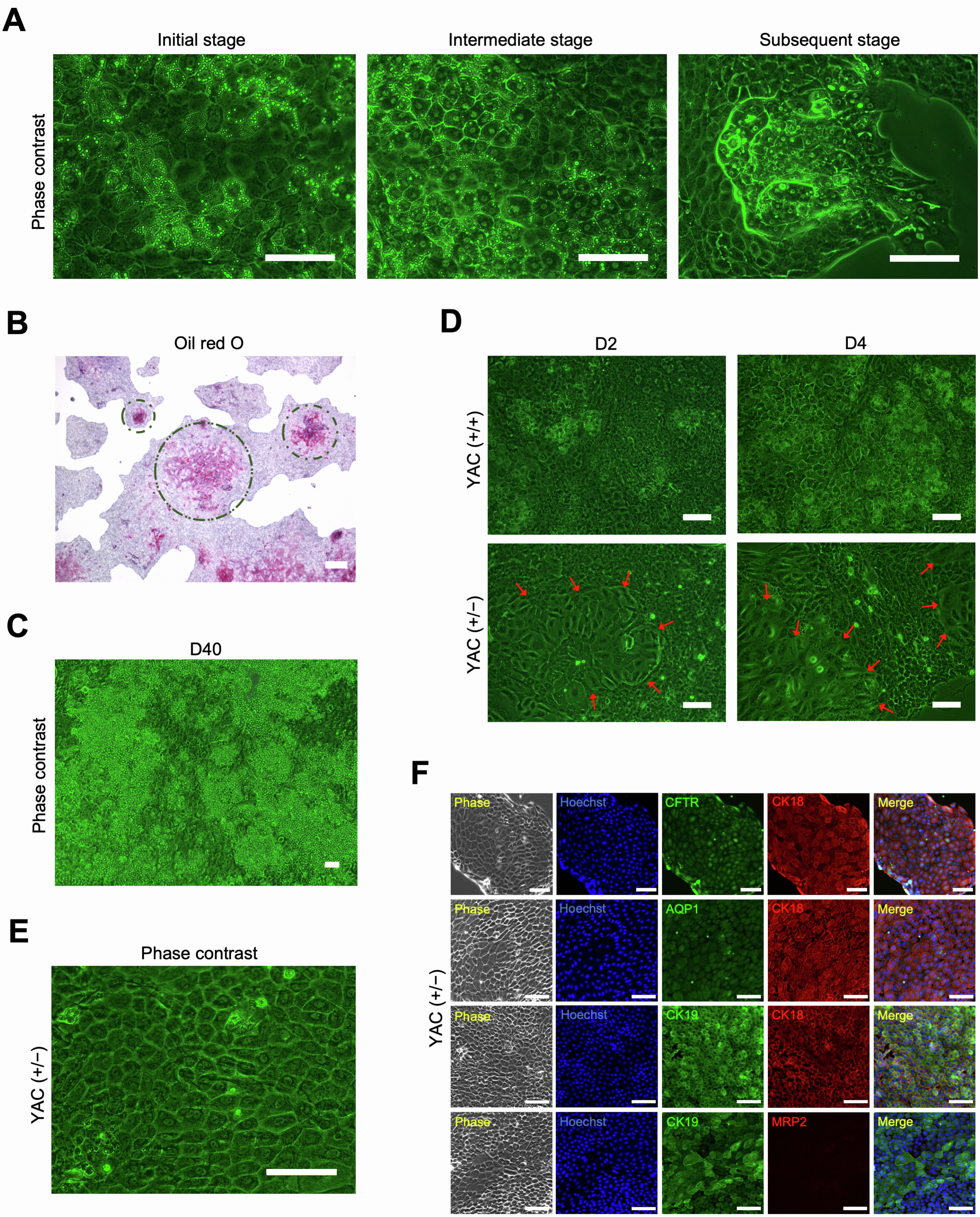
A
Initial stage
Intermediate stage
Subsequent stage
Phase contrast
B
Oil red O
C
D40
Phase contrast
D
D2
D4
YAC (+/+)
YAC (+/−)
E
Phase contrast
YAC (+/−)
F
Phase
Hoechst
CFTR
CK18
Merge
AQP1
CK19
MRP2
YAC (+/−)

Figure S4. Bidirectional spontaneous differentiation of small cells (related to Figure 4)

(A) Representative morphology of cells in the process of spontaneous differentiation to MHs. Scale bars, 100 μm. (B) Identification of lipid droplets in YAC-treated sorted small cells by oil red O staining. The dark-green closed loops indicate the position of LDCs in cell clusters. Scale bar, 300 μm. LDCs, cells containing lipid droplets. (C) The phase-contrast image showing the emergence of abundant lipid-droplet-containing cells in long-term culture. Scale bar, 100 μm. (D) Phase-contrast images of small cells treated with or without YAC withdrawal. The arrows indicate morphological changes in small cell. Scale bars, 100 μm. YAC (+/–), withdrawal of YAC from proliferated sorted small cells; YAC (+/+), maintenance of YAC in cultures of sorted small cells. (E) The phase-contrast image of cells with a low nucleus-to-cytoplasm ratio that appeared after YAC withdrawal. Scale bar, 100 μm. (F) Immunofluorescence staining of cells with a low nucleus-to-cytoplasm ratio for the cholangiocyte markers CK18, CK19, AQP1, and CFTR, and the MH marker MRP2. Scale bars, 100 μm.

Figure S5

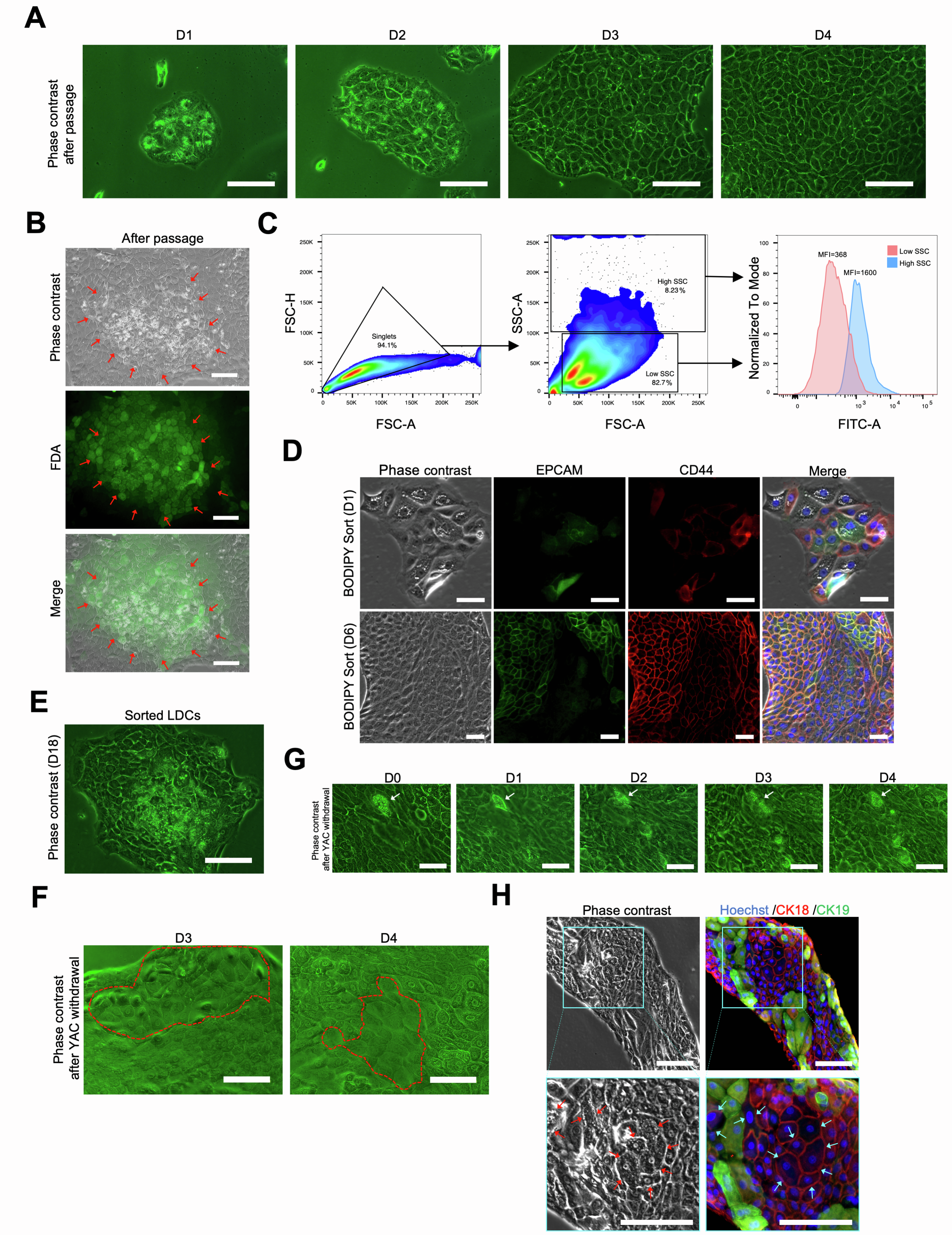

A
D1
D2
D3
D4
Phase contrast after passage
B
After passage
Phase contrast
FDA
Merge
C
FSC-H
Singlets 94.1%
FSC-A
SSC-A
High SSC 8.23%
Low SSC 82.7%
Normalized To Mode
MFI=368
MFI=1600
Low SSC
High SSC
FITC-A
D
Phase contrast
EPCAM
CD44
Merge
BODIPY Sort (D1)
BODIPY Sort (D6)
E
Sorted LDCs
Phase contrast (D18)
G
D0
D1
D2
D3
D4
Phase contrast after YAC withdrawal
F
D3
D4
Phase contrast after YAC withdrawal
H
Phase contrast
Hoechst /CK18 /CK19

Figure S5. Re-dedifferentiation of spontaneous mature hepatocytes into liver progenitor cells (related to Figure 5)

(A) Phase-contrast images of passaged LDCs exhibiting disappearance of lipid droplets after cells resumed proliferating. Scale bars, 100 μm. (B) Renewed spontaneous hepatocytic differentiation of small cells after passage, with enhanced synthetic capacity verified using an FDA assay. The arrows denote differentiated LDCs. Scale bars, 100 μm. FDA, fluorescein diacetate. (C) Gating strategy for LDC sorting. FITC, fluorescein isothiocyanate (used for the detection of BODIPY 493/503); MFI, mean fluorescence intensity. (D) Immunofluorescence staining of LPC markers EPCAM and CD44 in sorted LDCs that were cultured for 1 day and 6 days. Scale bars, 50 μm. (E) The phase-contrast image of sorted LDCs cultured for 18 days, showing the reappearance of lipid droplets. Scale bar, 100 μm. (F) Phase-contrast images of sorted LDCs that underwent YAC withdrawal. The closed loops indicate the position of morphologically altered cells. Scale bars, 100 μm. (G) Phase-contrast images of sorted cells that underwent YAC withdrawal, exhibiting the persistent presence of LDCs even after the elimination of YAC. The arrows denote LDCs. Scale bars, 100 μm. (H) Immunofluorescence

staining of cholangiocyte markers CK18 and CK19 in sorted cells that underwent YAC withdrawal. The arrows denote LDCs. Scale bars, 100 μm.

Table S1. Sequences of the primers used for qRT-PCR (related to Figures 2, 3, 4, 5, S1 and S2)

Gene	Forward primers (5′→3′)	Reverse primers (5′→3′)
Ae2	GACTCCTTTCCCTGTGTGGA	GAAGCATCCGCTCTTTCTTG
Afp	GCTGAACCCAGAGTACTGCAC	GACACGTCGTAGATGAACGTG
Alb	TGTCCCCAAAGAGTTTAAAGCTG	TCTTTATCTGCTTCTCCTTGTCTGG
Aqp1	CTGTGCGTTCTGGCTACCAC	GCACAGCAGAGCCAAATGAC
Aqp9	CTCAGTCCCAGGCTCTTCAC	TAAGACCTCCCAGGAAAGCA
C/ebp-α	GCCAAGAAGTCGGTGGATAA	CGGTCA TTGTCACTGGTCAA
Ccna	AACGATGAGCACGTCCCTACTGT	CAAGGATGGCCCGCATACTGTTA
Ccnb1	CCCTACCAAAACCTGTGGAC	CATCGGAGAAAGCCTGACAC
Ccnb2	TGGAGAGTGAAATACTGGAAGTCA	TGAGAAGCACACGATGGAAG
Ccnd1	TGCTTGGGAAGTTGTGTTGG	AATGCCATCACGGTCCCTAC
Ccne	GAAAATCAGACCGCCCAGAG	CGCTGCAGAAAGTGCTCATC
Cd44	GGCTTATCATCTTGGCATCC	CTGTTCCA TTGCCACTGTTG
Cdk1	GTACGGCAATCCGGGAAATC	GAGATACAGCCTGGAGTCCT
Cdk2	CATCAAGCTGGCTGACTTTGGA	GTGGAGTAGTACTTGCAGCCCAGAA

Cdk4	GGAGGCCTTTGAACATCCCA	ACTGGCGCATCAGATCCTTA
Cftr	GGTCATAGAGCAGGGCAATG	TGCACTTCTTCCTCCGTCTC
Ck18	TTCTGGGTTGACTGTGGAAGT	TGGTACTCTCCTCAATCTGCTG
Ck19	TTCCGGACCAAGTTTGAGAC	CCTCGTGGTTCTTCTTCAGG
Cyp3α1	TGGTAATAGACTTGAGAGAG	GGGCAGATATACATAAGGA
Cyp3α2	AGTAGTGACGATTCCAACATAT	TCAGAGGTATCTGTGTTTCCT
Epcam	TCGTGGTGGTGTTAGCAGTC	TCTGTGTATCTCACCCATCTCC
Foxj1	AGAACTGGGAACTGGGGACT	CTTCGGCTCTCGGAGTACAG
Grhl2	GTTCGATGCTCTGATGCTGA	GCAGCCCGTACTTCTCAGAC
Hnf4α	CTGCAGGCTCAAGAAGTGC	GGGAGGTGA TCTGCTGAGAC
Ki67	ATTTCAGTTCCGCCAATCC	GGCTTCCGTCTTCATACCTAAA
Mrp2	TGATCGGTTTCGTGAAGAGCT	ACGCACATTCCCAACACAAA
Sox9	TCCTAACGCCATCTTCAAGG	ACGTCTGTTTTGGGAGTGGT
Tat	ACCTCTGCTATGGGGCACT	ACTCCACGTCGTTCTCGAA T
Ttr	TCGTACTGGAAGGCTCTTGG	CCAGAGTCA TTGGCTGTGAA
β-actin	ATCTGGCACCACACCTTC	AGCCAGGTCCAGACGCA

Table S2. Key Resources Table (related to experimental procedures and supplemental experimental procedures)

REAGENT and RESOURCE	SOURCE	IDENTIFIER
**Antibodies**		
Mouse monoclonal anti-CD44	Cell Signaling	Cat#5640; RRID:AB_10547133
Mouse monoclonal anti-CD90	BioLegend	Cat#202501; RRID:AB_314013
Mouse monoclonal anti-CK18	Abcam	Cat#ab668; RRID:AB_305647
Mouse monoclonal anti-MRP2	Abcam	Cat#ab3373; RRID:AB_303751
Rabbit monoclonal anti-AFP	Abcam	Cat#ab213328; RRID:AB_2884974
Rabbit monoclonal anti-EPCAM	Abcam	Cat#ab213500; RRID:AB_2884975
Rabbit monoclonal anti-SOX9	Abcam	Cat#ab185230; RRID:AB_2715497
Rabbit polyclonal anti-ALB	GeneTex	Cat#GTX102419; RRID:AB_1949626
Rabbit polyclonal anti-AQP1	Sigma-Aldrich	Cat#SAB5200109; RRID:AB_2884973
Rabbit polyclonal anti-AXIN2	Abcam	Cat#ab32197; RRID:AB_2290204
Rabbit polyclonal anti-CFTR	Sigma-Aldrich	Cat#SAB4501942; RRID:AB_10745324

Rabbit polyclonal anti-CK19	Novus	Cat#NB100-687;<br>RRID:AB_2265512
Rabbit polyclonal anti-HNF4A	Bioss	Cat#bs-3828R;<br>RRID:AB_10856932
Rabbit polyclonal anti-ZO-1	Thermo Fisher Scientific	Cat#61-7300;<br>RRID:AB_2533938
Goat anti-rabbit Alexa Fluor 488	Cell Signaling	Cat#4412S;<br>RRID:AB_1904025
Goat anti-mouse Alexa Fluor 647	Abcam	Cat#ab150115;<br>RRID:AB_2687948
Goat anti-mouse APC	BioLegend	Cat#405308;<br>RRID:AB_315011
**Bacterial and virus strains**		
AAV.TBG.PI.Cre.rBG	Addgene	Addgene viral prep # 107787-AAV8;<br>RRID:Addgene_107787
**Chemicals, Peptides, and Recombinant Proteins**		
4% paraformaldehyde (PFA)	Wako	Cat#163-20145
7-aminoactinomycin D (7-AAD)	BD Biosciences	Cat#559925;<br>RRID:AB_2869266
A83-01	Wako	Cat#035-24113
BODIPY 493/503	Invitrogen	Cat#D3922
Bovine serum albumin (BSA)	Wako	Cat#015-23871
CHIR99021	Axon Medchem	Cat#1386

Collagenase II	Worthington Biochemical	Cat#LS004176
Deoxyribonuclease I (DNase I)	Worthington Biochemical	Cat#LS002139
Dexamethasone (Dex)	Wako	Cat#041-18861
DMEM/F12	Gibco	Cat#11320-033
Epidermal growth factor (EGF)	Sigma-Aldrich	Cat#SRP3238
Ethylenediaminetetraacetic acid (EDTA)	Invitrogen	Cat#15575-038
Fluorescein diacetate (FDA)	Sigma-Aldrich	Cat#F7378
FxCycle^{TM} PI/RNase Staining Solution	Invitrogen	Cat#F10797
HEPES	Gibco BRL	Cat#11344-033
Hoechst 33342	Thermo Fisher Scientific	Cat#62249
Insulin-transferrin-serine (ITS)-X	Gibco	Cat#51500-056
Isopropanol	Wako	Cat#164-08335
l-ascorbic acid 2-phosphate	Wako	Cat#013-12061
Live cell imaging solution	Invitrogen	Cat#A14291DJ
l -proline	Sigma-Aldrich	Cat#P5607
Matrigel Matrix	Corning	Cat#356237
MEF culture medium	Cosmo Bio	Cat#MEF-M
Methanol	Wako	Cat#131-01826
mTeSR^{TM}1 Complete Kit	STEMCELL Technologies	Cat#85850
Nicotinamide	Sigma-Aldrich	Cat#N3376
Oil red O	Sigma-Aldrich	Cat#O0625

Oncostatin M (OsM)	Sigma-Aldrich	Cat#SRP3250
Penicillin-streptomycin	Wako	Cat#168-23191
Percoll	GE Healthcare	Cat#17-0891-02
Phenol red-free MEMα	Wako	Cat#134-17225
Secretin	Tocris Bioscience	Cat#1919
Trypsin/EDTA	Wako	Cat#208-17251
Y-27632	Wako	Cat#036-24023
**Critical Commercial Assays**		
Cell Counting Kit-8 (CCK-8)	Dojindo Laboratories	Cat#347-07621
Click-iT® Plus EdU Imaging Kit	Invitrogen	Cat#C10640
PAS Staining Kit	Sigma-Aldrich	Cat#395B-1KT
Platinum SYBR Green qPCR Mix	Invitrogen	Cat#11744-500
PrimeScript RT Reagent Kit with gDNA Eraser	Takara Bio	Cat#RR047A
Rat Albumin ELISA Kit	Bethyl	Cat#E111-125
RNeasy Mini Kit	Qiagen	Cat#74106
Urea Assay Kit	Abcam	Cat#ab83362
**Experimental Models: Cell Lines**		
Mouse: Embryonic fibroblasts (MEF)	Cosmo Bio	Cat#MEF-01C
**Experimental Models: Organisms/Strains**		
Sprague-Dawley *Rat*	Japan SLC	Slc:SD
Rosa26-LSL-tdTomato *Rat*	NBRP-Rat	NBRP-Rat NO.0734

**Oligonucleotides**		
Primers used for qRT–PCR, see Table S1	This paper	N/A
**Software and Algorithms**		
ImageJ	https://imagej.nih.gov/ij/	RRID:SCR_003070
FlowJo V10	FLOWJO, LLC	RRID:SCR_008520
Graph Pad Prism V7.0	GraphPad Software	RRID:SCR_002798

Supplemental Experimental Procedures

Isolation of MHs

Whole cells of the rat liver were obtained using a method reported previously by us [1], whereas the centrifugation strategy used for isolating MHs was in accordance with another procedure [2]. In brief, the liver was perfused with 60 mL of Ca^{2+}/Mg^{2+}-free Hank's Balanced Salt Solution (HBSS (–); Gibco) containing 1 mM ethylenediaminetetraacetic acid (EDTA; Invitrogen) at 18 mL/min via the portal vein after anesthesia, followed by perfusion with 200 mL of HBSS (–) supplemented with 100 U/mL of collagenase II (Worthington Biochemical) at 7.5 mL/min. Subsequently, we peeled the capsule of the well-digested liver with tweezers and performed further digestion with 120 mL of HBSS (–) containing 65 U/mL of collagenase II and 400 U/mL of deoxyribonuclease I (DNase I; Worthington Biochemical) at 37°C with shaking on a stirring plate at 70 rpm for 20 min. The obtained cell suspension was filled up to 160 mL with HBSS (–) and filtered twice, using first a 100-μm and then a 70-μm cell strainer (Falcon). Next, we split the filtrate four ways into 50-mL tubes and performed centrifugation for 10 min at 600×*g* at 4°C. The pellets were gathered into two tubes, then rinsed with 40 mL of

HBSS (–) containing 120 U/mL of DNase I in each tube and centrifugated for 1 min at 60×*g* at 4°C. This step was repeated once. Cells were collected into one tube and resuspended in 20 mL of HBSS (–) containing 120 U/mL of DNase I, then mixed with 100% Percoll (GE Healthcare; Prepared in advance with Percoll stock solution and 1.5 M NaCl solution at a ratio of 9:1) to a final concentration of 48.9% (v/v). Cells in the pellet obtained after centrifugation for 10 min at 60×*g* at 4°C were regarded as purified MHs and were used in follow-up experiments after being centrifuged twice at 60×*g* for 2 min and filtered once with a 40-μm cell strainer (Falcon). A Luna automated cell counter (Logos Biosystems) was used to perform the cell count and measure cell size.

Isolation of LPCs

The supernatant obtained after the first centrifugation at 60×*g* during the isolation of MHs was used to obtain LPCs [3]. First, the supernatant was centrifugated at 50×*g* for 5 min at 4°C, followed by the dissociation of the pellet by adding 40 mL of HBSS (–) containing 120 U/mL of DNase I in each tube and an additional centrifugation under the same conditions. Cells were collected and rinsed with 40 mL of HBSS (–) containing 120 U/mL of DNase I in each tube, followed by

centrifugation for 5 min at 150×*g* at 4°C. This step was repeated once, and then the cells were gathered into one tube and rinsed with 40 mL of HBSS (–) containing 120 U/mL of DNase I. After centrifugation at 50×*g* for 5 min at 4°C, we filtered cells with a 40-μm cell strainer and obtained crude LPCs mixed with other NPCs.

Cell Culture Models

Typically, cells were cultured in a humidified atmosphere of 95% air and 5% CO_2 at 37°C, while a hypoxic environment was set at 37°C, 5% O_2 and 5% CO_2. SHM was used as the basal medium for cell culture [3], *viz*, DMEM/F12 (Gibco) containing 2.4 g/L $NaHCO_3$ and L-glutamine, which was supplemented with 5 mM HEPES (Gibco BRL), 30 μg/mL of L-proline (Sigma-Aldrich), 0.05% bovine serum albumin (BSA; Wako), 10 ng/mL of epidermal growth factor (Sigma-Aldrich), 1% insulin-transferrin-serine (ITS)-X (Gibco), 10^{-7} M dexamethasone (Dex; Wako), 10 mM nicotinamide (Sigma-Aldrich), 1 mM L-ascorbic acid 2-phosphate (Wako), and 1% penicillin–streptomycin solution (Wako). YAC was formulated as follows [4]: 10 μM Y-27632 (Wako), 0.5 μM A83-01 (Wako), and 3 μM CHIR99021 (Axon Medchem).

To verify the real role of YAC, we set up a variety of culture models by adjusting the timing of the addition and withdrawal of YAC. Unless otherwise stated, the

corresponding medium was replaced every other day. A Luna Automated Cell Counter was used to perform cell counting. During each cell culture process, image acquisition was carried out using an IX70 Inverted Tissue Culture Microscope (Olympus) and a DS-L3 Digital Camera Controller (Nikon). Image processing and relevant calculations were achieved using the ImageJ software (https://imagej.nih.gov/ij/).

The primary MHs were seeded on collagen type 1-coated plates (Iwaki) at 1×10^4 cells/cm^2 and cultured in SHM with or without YAC, accompanied by a change of the medium 1 day after seeding and every 2 days thereafter.

To verify if YAC promoted small-cell proliferation, YAC (–) cells were cultured until small-cell clusters emerged; then, the medium was replaced with YAC-containing medium (YAC (–/+) cells), which was recorded as D0. Cell culture lasted for 10 days, and YAC (–) cells cultured without YAC in the same period (YAC (–/–) cells) were used as controls. For comparison, multiple identical cell clusters were continuously observed.

Cells sorted from YAC (–) cells (YAC (–) Sort cells) and YAC (+) cells (YAC (+) Sort cells) on D22 were used to further investigate the effects of YAC on small cells.

The day of sorting was recorded as D0, and the YAC (–) Sort cells were cultured in the presence of YAC (YAC (–/+)* cells) or continued to be cultured using SHM alone (YAC (–/–)* cells) for 10 days. Similarly, YAC (+) Sort cells were cultured in the same manner, namely, in the form of YAC (+/+)* cells and YAC (+/–)* cells.

To examine the origin of small cells, we treated MHs (pellet-derived cells) and LPCs (supernatant-derived cells) under YAC stimulation during a 14-day culture. Homologous cells cultured with SHM throughout the corresponding period were set as controls.

For the purpose of assessing whether YAC had any effect on already proliferated small cells, YAC (+) Sort cells were cultured in the presence of YAC (YAC (+/+) cells) until 80% confluence, followed by the withdrawal of YAC (YAC (+/–) cells) for 4 days. Cells that were always cultured in medium containing YAC were used as a control.

FACS for Small Cells and Cells Containing Lipid Droplets (LDCs)

The MHs or crude LPCs cultured with YAC could be sorted on D14, whereas the sorting of cells cultured without YAC stimulation was performed on D22. The cells were collected using 0.5% trypsin/EDTA (Wako) and resuspended in phenol-red-

free MEMα (Wako) supplemented with 1.5% fetal bovine serum (FBS; Sigma-Aldrich) after centrifugation for 5 min at 400 × *g*. The suspension was filtered using a 40-μm cell strainer and adjusted to a final concentration of 1 × 10^7 cells/mL. 7-Aminoactinomycin D solution (7-AAD, 2 μL/10^6 cells; BD Biosciences) was added to the suspension immediately before sorting. A 15 mL polypropylene tube containing 10 mL of SHM was used to collect the sorted cells.

Small cells were cultured with YAC until plenty of LDCs appeared. First, cells were incubated with 2 μg/mL of BODIPY 493/503 (Invitrogen) for 30 min at 37°C. The cells were then collected using 0.5% trypsin/EDTA and resuspended in phenol-red-free MEMα supplemented with 1.5% FBS after centrifugation for 5 min at 400 × *g*. The suspension was filtered using a 40-μm cell strainer, and 7-AAD (2 μL/10^6 cells) was added to the suspension immediately before sorting. A 15 mL polypropylene tube containing 10 mL of SHM supplemented with YAC was used to collect the sorted LDCs.

A FACS Aria III Cell Sorter (BD Biosciences) was used to perform FACS, and the data were analyzed using FlowJo software (FLOWJO, LLC). The collected cells were centrifuged for 5 min at 400×*g* at 4°C, followed by seeding on collagen

type 1-coated plates at 1.5×10^4 cells/cm^2.

***In vitro* lineage tracing of rat MHs**

AAV8-TBG-Cre was injected to a 16-week-old male Rosa26-LSL-tdTomato rat from the tail vein at 1.1×10^{12}GC. After 7 days, we sacrificed the rat and isolated MHs. On the other hand, we sorted tdTomato$^+$ MHs using an SH800 Cell Sorter (Sony Biotechnology) with 561nm laser. Unsorted MHs and sorted tdTomato$^+$ MHs were cultured with or without YAC using the method described above.

Hepatocytic Induction of Small Cells

Small cells sorted from YAC-treated cells were induced into MHs. The sorted cells were cultured with YAC until 50% cell confluence. Subsequently, for hepatocytic induction (Hep-i (+) cells), the culture medium was supplemented with 20 ng/mL oncostatin M (OsM; Sigma-Aldrich) and 10^{-6} M Dex [5] during a 6-day culture, with a change into fresh medium performed every other day. On D6, the cultured small cells were overlaid with the mixture of Matrigel (Corning) and the hepatic induction medium at a 1:7 ratio. The cell culture was continued for another 3 days, or 5 days with one replacement of the Matrigel mixture, to end the induction. Before various assays, the covered Matrigel had to be removed via gentle aspiration. Sorted small

cells that were cultured with YAC alone throughout the corresponding culture period served as the negative control (Hep-i (–) cells).

Cholangiocytic Induction of Small Cells

The cholangiocytic induction referred to a previous procedure [4]. Mitomycin C-treated mouse embryonic fibroblasts (MEFs; Cosmo Bio) were inoculated on collagen type 1-coated 6-well plates at 1×10^5 cells/well and cultured with MEF culture medium (Cosmo Bio). On the following day, cultured YAC (+) Sort small cells were harvested using trypsin/EDTA and cultured on pre-seeded MEFs at 1×10^6 cells/well with YAC and 5% FBS-supplemented medium for 24 h. Subsequently, cholangiocytic induction (BEC-i (+) cells) was initiated by replacing the medium with mTeSR™1 complete medium (STEMCELL Technologies) containing YAC. This process of induction lasted 6 days, and the medium was renewed every 2 days. Matrigel (2%) was added to the induction medium on D6, to complete the cholangiocytic induction, and the cells were cultured for an additional 6 days, with the medium being replaced every other day. After this 12-day induction, the cells were analyzed in various ways. As a negative control (BEC-i (–) cells), sorted small cells were cultured on MEFs only with YAC throughout the corresponding culture

period.

RNA Isolation and qRT–PCR

RNA of the isolated or cultured cells was extracted using an RNeasy Mini Kit (Qiagen) according to the manufacturer's instructions. Total RNA was reverse transcribed into cDNA using a PrimeScript RT Reagent Kit with gDNA Eraser (Takara Bio) in a Veriti 96-well Thermal Cycler (Applied Biosystems) using the following conditions: incubation at 37°C for 15 min and at 85°C for 15 s. qPCR was carried out in a total reaction volume of 25 μL containing 5 μL of template cDNA mixture, 12.5 μL of a Platinum SYBR Green PCR Mix (Invitrogen), and 2 μL of a 10 μM corresponding primer mixture. The PCR conditions on a Step One Plus Real Time PCR System (Applied Biosystems) included pre-denaturation at 95°C for 20 s, followed by 40 cycles of 95°C for 3 s and 60°C for 7 s. The relative expression was determined using the relative standard curve method and *β-actin* was used as an endogenous control. Table S1 shows the sequences of the primers used in this experiment.

Flow Cytometry Analysis

We harvested the cultured Hep-i (+) cells using 0.5% trypsin/EDTA and fixed them

with precooled (–30°C) methanol (Wako) for 10 min at room temperature (RT). The cells were then centrifuged and washed with phosphate-buffered saline (PBS; Gibco) at 400×*g* for 2 min. PBS containing 1% (w/v) BSA was used to block the cells for 30 min at RT. Next, the cells were incubated with primary antibodies against ALB (1:300; GeneTex), CK18 (1:250; Abcam), HNF4A (1:200; Bioss), and MRP2 (1:200; Abcam) for 1 h at RT, respectively. After washing twice with PBS at 400×*g* for 2 min each time, the cells were incubated with the following secondary antibodies, anti-rabbit Alexa Fluor 488 (1:800; Cell Signaling Technology) or anti-mouse APC (1:400; BioLegend), depending on the species in which the primary antibody was raised, for 30 min at RT in the dark, followed by a final wash in PBS at 400×*g* for 2 min. All prepared cells were resuspended in HBSS (–), filtered using a 40-μm strainer, and analyzed using a FACS Canto II Flow Cytometer (BD Biosciences).

Immunofluorescence Staining

The cultured cells were washed three times using HBSS (–) and then fixed in precooled (–30°C) methanol for 10 min at RT. After washing with HBSS (–), blocking was performed with PBS containing 1% (w/v) BSA for 30 min at RT.

Subsequently, the cells were incubated with primary antibodies for 1 h at RT, followed by incubation with secondary antibodies for 30 min at RT in the dark. All antibodies were prepared in PBS and were diluted as follows: AFP, CD90, CK18, CK19, CFTR, EPCAM, HNF4A, MRP2, and ZO-1, 1:100; ALB, AXIN2, AQP1, and SOX9, 1:200; anti-rabbit Alexa Fluor 488, and anti-mouse Alexa Fluor 647, 1:500. Finally, the nuclei were stained with Hoechst 33342 (1:800; Thermo Fisher Scientific) for 3 min at RT and washed with HBSS (–) for observation. A FluoView FV10i Confocal Laser Scanning Microscope (Olympus) was used to complete the image acquisition and analysis.

Cell Cycle Analysis

A part of YAC (–) Sort cells were fixed using precooled (–30°C) 70% ethanol and stored at –30°C till analyzing. The rest YAC (–) Sort cells were cultured with YAC and collected when achieving 30% confluence. Cultured cells were also fixed by 70% ethanol (–30°C). All cells were washed with PBS and then stained with FxCycle^{TM} PI/RNase Staining Solution (Invitrogen) for 30 min. Stained cells were analyzed using a FACS Canto II Flow Cytometer and the data was analyzed using the FlowJo software.

Oil Red O Staining

Lipid was detected in the cells by oil red O staining. The oil red O stock solution was prepared by dissolving 0.5 g of oil red O powder (Sigma-Aldrich) in 100 mL of isopropanol (Wako), and the oil red O working solution was formulated by mixing the oil red O stock solution with ddH_2O at a ratio of 3:2. Cultured YAC (+) sort small cells were washed with PBS and fixed with 4% paraformaldehyde (PFA) for 5 min at RT. Next, the cells were washed three times using PBS and rinsed with 60% isopropanol for 2 min. The freshly prepared oil red O working solution was filtered with filter paper (Advantec) and used to stain the cells for 15 min at RT, followed by rinsing with 60% isopropanol for 30 s. Nuclei were stained with hematoxylin (Sigma-Aldrich) for 90 s, and the cells were prepared for observation after washing with PBS. A BZ-9000 All-In-One Fluorescence Microscope (Keyence) was used to scan the samples.

PAS Staining

PAS staining was performed to determine glycogen using a PAS Staining System (Sigma-Aldrich). The cell preparation and staining strategy was according to the standard procedures provided by the manufacturer. Image acquisition was

performed using a BZ-9000 All-In-One Fluorescence Microscope.

BODIPY 493/503 Staining

BODIPY 493/503 was added to the culture medium at a final concentration of 2 μg/mL. After incubating for 30 min at 37°C, small cells were rinsed twice with Hank's Balanced Salt Solution (HBSS (+); Gibco) and then overlaid with a live-cell imaging solution (Invitrogen) for scanning. A FluoView FV10i Confocal Laser Scanning Microscope was used to acquire the images.

EdU Assay

Proliferating cells were identified by EdU assay using a Click-iT® Plus EdU Imaging Kit (Invitrogen). Before detection, the cells were incubated with the corresponding medium containing 12.5 μM EdU for 24 h, followed by treatment according to the manufacturer's experimental protocols. A FluoView FV10i Confocal Laser Scanning Microscope was used to acquire images.

ALB Secretion Assay

The secreted ALB concentration was measured using a Rat Albumin ELISA Kit (Bethyl), according to the manufacturer's instructions. Culture supernatants collected from Hep-i (+) cells and Hep-i (–) cells on D9 or D11 were used as test

samples; the Matrigel overlaying the Hep-i (+) cells was collected after being converted into a liquid at 4°C. The mean cell number counted on D9 and D11 was used for normalizing the ALB secretion from D6–D9 and D9–D11, respectively. Cell-free YAC-containing SHM and cell-free Matrigel-mixed hepatic induction media were incubated for the corresponding periods and were regarded as blank controls to establish the baseline. A SpectraMax Paradigm Multi-Mode Detection System (Molecular Devices) was used to measure the absorbance and calculate the results.

Urea Synthesis Assay

A Urea Assay Kit (Abcam) was used to determine the secreted urea concentration. The test samples and blank controls prepared for the ALB secretion assay were also applied to the urea synthesis assay. The urea secretion from D6–D9 and D9–D11 was quantified according to the manufacturer's instructions and normalized to the mean cell number counted on D9 or D11. A SpectraMax Paradigm Multi-Mode Detection System performed the output measurement and calculation of the results.

FDA Hydrolysis Assay

An FDA assay was used to evaluate the secretory function of Hep-i (+) cells, and to indicate the synthesis capacity of different cells. Cells were incubated with a corresponding medium containing 2.5 μg/mL of FDA (Sigma-Aldrich) in an incubator for 15 min, and then the medium was replaced with HBSS (+) for observation. The hydrolysates of FDA emitted green fluorescence, and their distribution was detected by a FluoView FV10i Confocal Laser Scanning Microscope or a BZ-9000 All-In-One Fluorescence Microscope.

Secretin Assay

The secretory capacity of BEC-i (+) cells was determined by a secretin assay. After washing with PBS 3 times, cells were cultured with HBSS (+) containing 2×10^{-7} M rat secretin (Tocris Bioscience) for 1 h. Image acquisition was performed by a FluoView FV10i Confocal Laser Scanning Microscope.

Proliferation Assay

The proliferation of cells cultured under diverse conditions was measured using a Cell Counting Kit-8 (CCK-8; Dojindo Laboratories). Cells were seeded onto a collagen type I-coated 96-well plate at an initial density of 1×10^4 cells/well, and then cultured under the corresponding conditions and periods with replacement

with fresh medium every 2 days. Cell-free medium cultured during the same period was used as a blank control. For each test, 10 μL/well of CCK-8 solution was added to the medium (including the blank controls) and the cells were cultured for 90 min in an incubator. A GloMax-Multi+ Detection System (Promega) was used to determine absorbance.

Statistical Analysis

All data are presented as the mean±standard deviation (SD). Intergroup differences were identified using one-way analysis of variance, followed by Tukey's multiple comparisons test. Multiple *t*-tests, corrected using the Holm–Sidak method, were used to analyze longitudinal data. Unpaired *t*-tests or Welch's test were applied to determine pairwise differences, depending on whether variance was equal. Differences at P (or adjusted P)<0.05 were considered statistically significant, and Graph Pad Prism 7.0 (GraphPad Software) was used to perform the statistical analysis.

Supplemental References

1. Fu Q, Ohnishi S, Sakamoto N. Conditioned Medium from Human Amnion-Derived Mesenchymal Stem Cells Regulates Activation of Primary Hepatic Stellate Cells. Stem Cells Int. 2018;2018:4898152.
2. Seglen PO. Preparation of isolated rat liver cells. Methods Cell Biol. 1976;13:29-83.
3. Chen Q, Kon J, Ooe H, Sasaki K, Mitaka T. Selective proliferation of rat hepatocyte progenitor cells in serum-free culture. Nat Protoc. 2007;2(5):1197-205.
4. Katsuda T, Kawamata M, Hagiwara K, Takahashi RU, Yamamoto Y, Camargo FD, et al. Conversion of Terminally Committed Hepatocytes to Culturable Bipotent Progenitor Cells with Regenerative Capacity. Cell Stem Cell. 2017;20(1):41-55.
5. Kamiya A, Kojima N, Kinoshita T, Sakai Y, Miyaijma A. Maturation of fetal hepatocytes in vitro by extracellular matrices and oncostatin M: induction of tryptophan oxygenase. Hepatology. 2002;35(6):1351-9.
